# Identification of specific DNA methylation sites on the Y-chromosome as biomarker in prostate cancer

**DOI:** 10.18632/oncotarget.6141

**Published:** 2015-10-16

**Authors:** Lushuai Yao, Shancheng Ren, Minjie Zhang, Fengxia Du, Yasheng Zhu, Hui Yu, Chenyu Zhang, Xiaohua Li, Caiyun Yang, Huixian Liu, Dong Wang, Hao Meng, Shuang Chang, Xiao Han, Yinghao Sun, Yingli Sun

**Affiliations:** ^1^ Key Laboratory of Genomic and Precision Medicine, Beijing Institute of Genomics, Chinese Academy of Sciences, Beijing, China; ^2^ University of Chinese Academy of Sciences, Beijing, China; ^3^ Department of Urology, Shanghai Changhai Hospital, Second Military Medical University, Shanghai, China

**Keywords:** DNA methylation, prostate cancer, Y-chromosome, biomarker

## Abstract

As a diagnostic biomarker, prostate special antigen (PSA) tests always generate false positive results and lead to unnecessary and/or repeat biopsies. Therefore, there is an urgent need for developing more sensitive, specific diagnostic biomarkers. We epigenotyped methylated sites in cancer tissues and adjacent normal tissues from 66 patients. In comparation with normal adjacent tissues, we observed that there were 6 aberrant methylation sites in prostate cancer tissues on the Y-chromosome. We further performed pyrosequencing using urine of PCa patients and we identified one methylated site (cg05163709) as a potential biomarker. We evaluated the predictive capacity of the aberrant methylated sites using the area under receiver operating characteristic (ROC) curve (AUC). The ROC analysis showed a higher AUC for cg05163709 (0.915) than prostate-specific antigen (PSA, 0.769). These results indicated that aberrant DNA methylation of cg05163709 on the Y-chromosome could serve as a potential diagnostic biomarker with high sensitivity and specificity.

## INTRODUCTION

Prostate cancer (PCa) is the second cancer-related killer for men in the Western world [1]. Early diagnosis is crucial for PCa diagnosis and treatment. Prostate-specific antigen (PSA) test, which has been used in the early diagnosis, lacks specificity and sensitivity, which lead to unnecessary biopsies [2–4]. Therefore, there is an urgent need to develop sensitive, non-invasive and cost-effective early detection biomarkers.

Alteration of DNA methylation is often identified in cancer and is associated with cancer initiation and progression [5, 6]. A number of studies have identified some novel specific DNA methylation sites as biomarkers of PCa. For example, hypermethylation of some important genes, such as *GSTP1*, *PITX2* and the *GABRE~miR-452~miR-224*, has shown promise as biomarkers of prostate cancer [7, 8].

The Y-chromosome is the sex-determining chromosome in many species [9, 10]. Loss of it can result in some diseases such as hematologic disease, acute myelogenous leukemia and myelodysplastic syndrome [11–14]. Therefore the Y-chromosome plays a critical role in tumor suppression both in vivo and in vitro [15, 16]. In PCa, it is often detected that recurrent copy number loss at Yp11.2 was involved with the *TSPY* gene cluster [17]. However, whether the epigenetic modification of the Y-chromosome is associated with PCa progression is currently unclear.

In this study, we demonstrated the correlation between the DNA methylation of the Y-chromosome and PCa. Comparing PCa with adjacent normal tissues, we screened 6 methylated sites which were conservative in adjacent normal tissues but varied remarkably in cancer tissues. Two of the six aberrant methylation sites were also detected in the urine of PCa patients using pyrosequencing. In addition, the receiver operating characteristic (ROC) analysis showed that the cg05163709 site was a promising diagnostic biomarker with high sensitivity and specificity.

## RESULTS

### Characterization of prostate cancer (PCa) tissues and adjacent normal (NA) tissues

**Figure 1:**
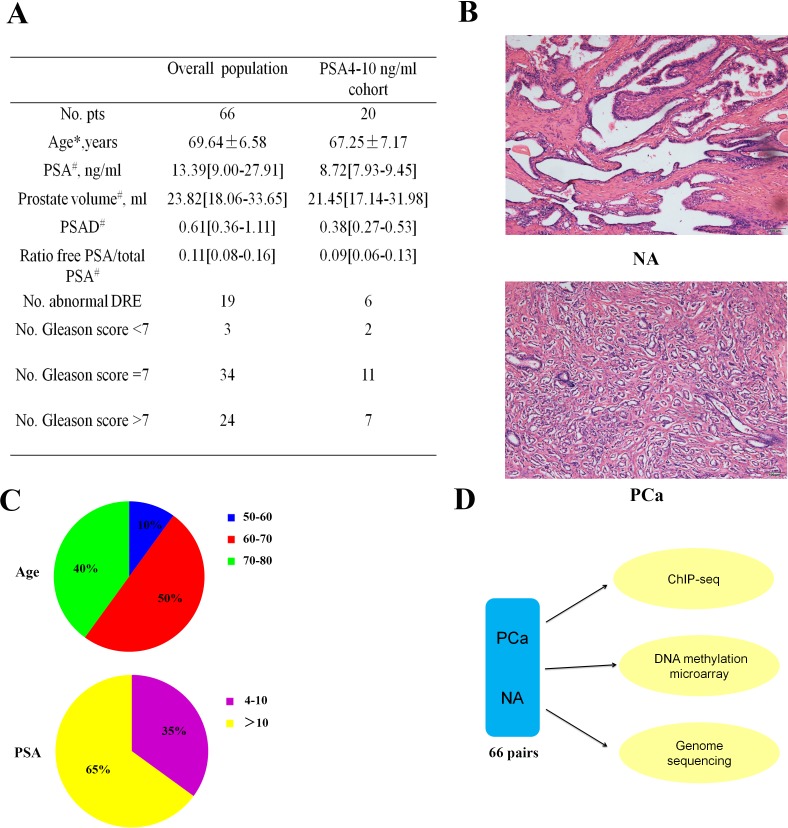
Characteristics of the prostate cancer (PCa) samples **A.** Clinical and pathological information on the study cohort. (*Age is reported as means ± SD. ^#^These data are reported as medians [interquartile ranges - IQRs]. #Clinically significant PCa according to the Epstein criteria: clinical stage ≥ T2, Gleason score ≥ 7, PSAD > 0.15, and > 33% positive cores on biopsy. Abbreviations: DRE = digital rectal examination; PCa = prostate cancer; PSA = prostate-specific antigen; PSAD = PSA density (serum PSA/prostate volume). **B.** Representative normal adjacent (NA, upper) and PCa tissues (PCa, lower) H&E sections from one patient. Scale bar: 100 μm. **C.** Distributions of the age (years) and PSA concentrations (ng/ml). **D.** Workflow for determining the relationship between the Y-chromosome and PCa.

We took 24 samples of blood (12 from PCa patients and 12 from healthy individuals) and 66 pairs of PCa and NA (Table S1). Clinical and pathological information of the study cohort was outlined in Figure 1A. Two pathologist assessed all 66 pairs of PCa adjacent normal tissues using hematoxylin and eosin (H&E)-stained sections (Figure 1B) independently. Most patients were aged 60 to 80 years, and more than half of the patients' PSA concentrations were greater than 10ng/mL (Figure 1C). Chromatin immunoprecipitation (ChIP)-seq, genome sequencing, and DNA methylation microarray were performed to determine the correlation between the DNA methylation pattern of Y-chromosome and PCa (Figure 1D).

### H3K4me3 modification on Y-chromosome showed no significant change between PCa and NA

**Figure 2:**
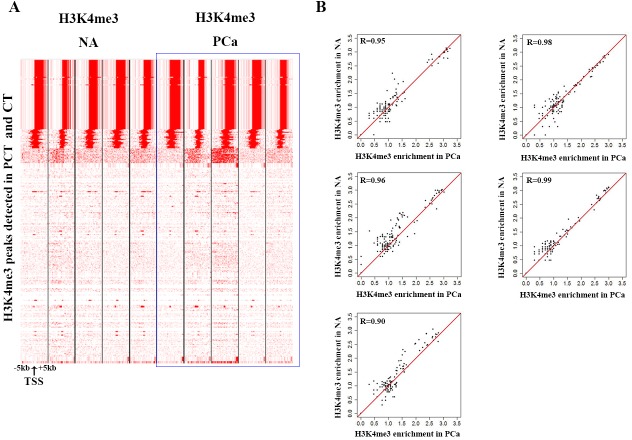
H3K4me3 modification is stable on the Y-chromosome **A.** Heatmap displaying the density of aligned ChIP-seq reads for H3K4me3 within a 10-kb window. **B.** Pearson correlation coefficients between adjacent normal tissues and PCa of five pairs of samples.

We performed ChIP-seq of H3K4me3 in cancer tissues and normal adjacent tissues of paired samples from 5 patients. We found no significant difference of H3K4me3 modification between PCa and NA (Figure 2A and 2B). The results indicated that H3K4me3 modification on Y-chromosome could not serve as a potential biomarker.

### The methylation levels of certain sites on the Y-chromosome changed remarkably in PCa

**Figure 3:**
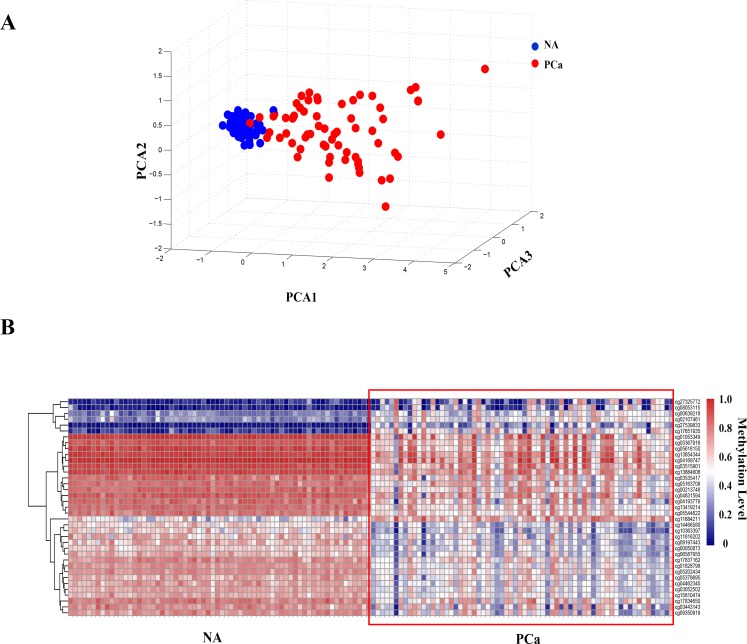
DNA methylation level in prostate cancer (PCa) is clearly different from adjacent normal (NA) tissues **A.** The principal component analysis of the methylation pattern on the Y-chromosome between PCa and adjacent normal tissues. **B.** Heat map of the DNA methylation levels of the 37 methylated sites that changed markedly in PCa (|Δβ-value| ≥ 0.2, *p* < 0.01, FDR-adjusted *p* < 0.01).

Then we did DNA methylation array using the Illumina 450K methylation microarray platform to detect the methylation level of the Y-chromosome. In order to find out whether aberrant methylation occured on the Y-chromosome in PCa tissues, we compared the DNA methylation level between cancer tissues and adjacent normal tissues of paired samples from 66 patients. We found that the methylation levels of a number of sites were obviously different in PCa (Figure S1). Through principal component analysis of the methylation levels of all of the tested sites on the Y-chromosome, we found that adjacent normal tissues clustered into a class, indicating that their methylation levels were similar with each other, whereas the PCa tissues were heterogeneous (Figure 3A). Taken together, these results demonstrated that the methylation levels of the Y-chromosome changed remarkably in PCa. Ultimately, we identified 37 differentially methylated sites (Wilcoxon rank-sum-test, *p* < 0.01; false-discovery rate [FDR]-adjusted *p* < 0.01; |Δβ-value | ≥ 0.2,) using the Illumina Methylation Analyzer (IMA) package in the R statistical language (Figure 3B, Table S2).

### 6 of these 37 differential methylation sites were conservative in adjacent normal tissues

**Figure 4:**
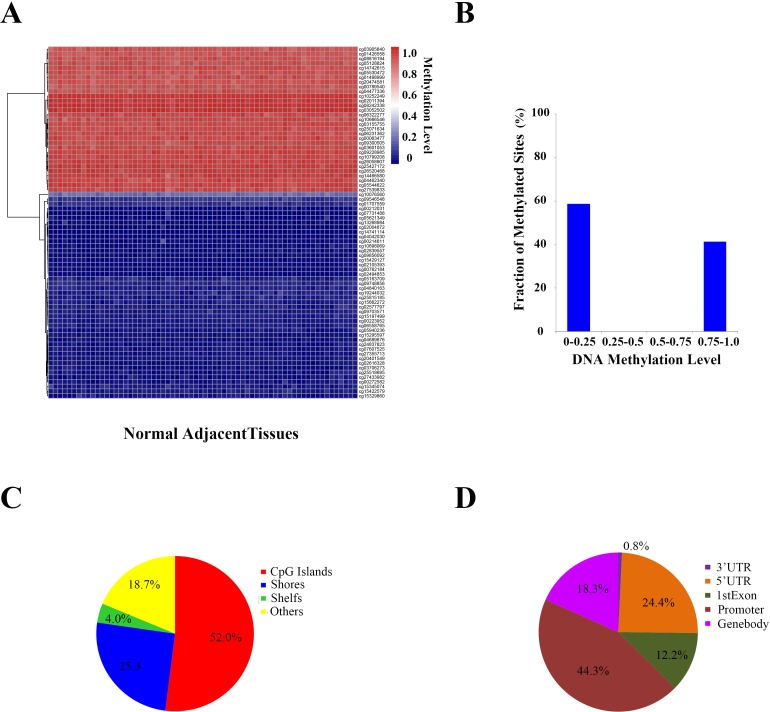
DNA methylation patterns on the Y-chromosome adjacent normal (NA) tissues **A.** DNA methylation level of the conservative methylated sites on the Y-chromosome in each prostate and adjacent normal tissue. **B.** Ratios of the conservative methylated sites (β-value < 0.25 means low methylation values, β-value > 0.75 means heavy methylation values, and 0.25 ≤ β-value ≤ 0.75 means intermediate methylation values). **C.** Characteristics of the conservative methylated sites. **D.** Location distribution of the conservative methylated sites.

To determine whether the methylation level of these differential methylation sites were only presented variation in cancer tissues while conservative in adjacent normal tissues, we analyzed the DNA methylation level of the Y-chromosome of the 66 samples of adjacent normal tissues for contrast. After filtering 35 sites containing missing values in NA, 381 sites were remained. Finally, we identified 75 conservative sites (SD < 0.25) (Figure 4A). Among them, 58.67% were hypomethylated (β-value ≤ 0.25), and 44.33% were hypermethylated (β-value ≥ 0.75) (Figure 4B). Notably, we also found that 52.0% of the sites were located in CpG islands (Figure 4C) and 44.3% in promoter regions (Figure 4D).

**Figure 5:**
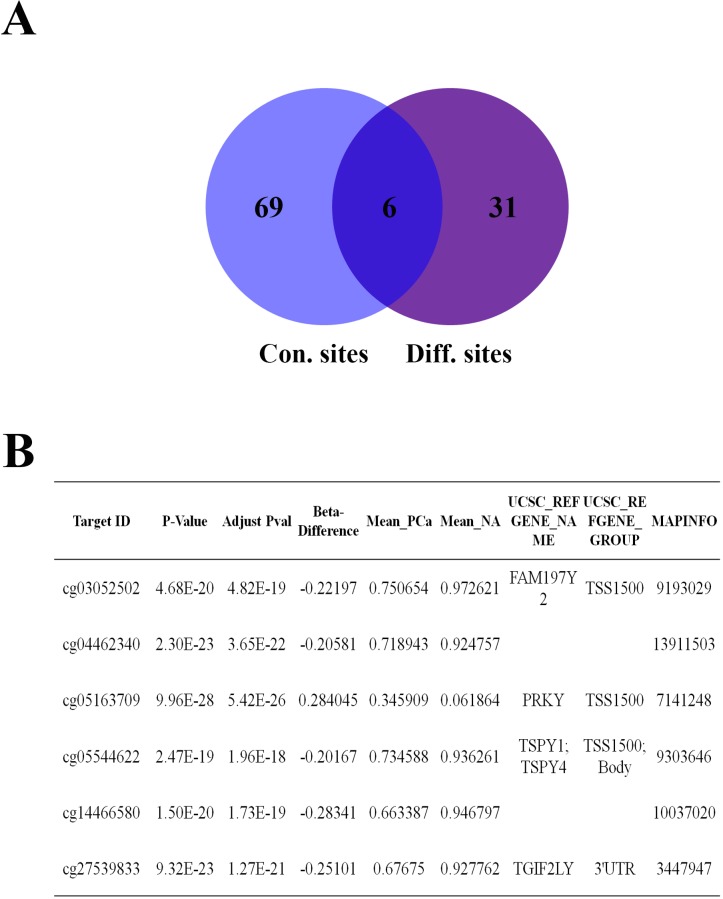
6 aberrant methylated sites were conservative in prostate tissues **A.** The Venn diagram of conservative sites and different sites. **B.** Detailed information on the six methylated sites.

In addition, we compared the 37 differential methylation sites in cancer tissues to these 75 conservative sites. Eventually, we found that 6 sites (Target ID: cg03052502, cg04462340, cg05163709, cg05544622, cg14466580 and cg27539893) were in the intersection of them (Figure 5A). Interestingly, these sites were highly similar in NA but very different in PCa tissues (Figure S2). cg03052502, cg05163709 and cg05544622 were located in the promoters of *FAM197Y2, PRKY* and *TSPY4*, respectively. cg27539893 was located in the 3′ untranslated region (UTR) of *TGIF2LY*. cg04462340 and cg14466580 were located in intergenic regions (Figure 5B).

**Figure 6:**
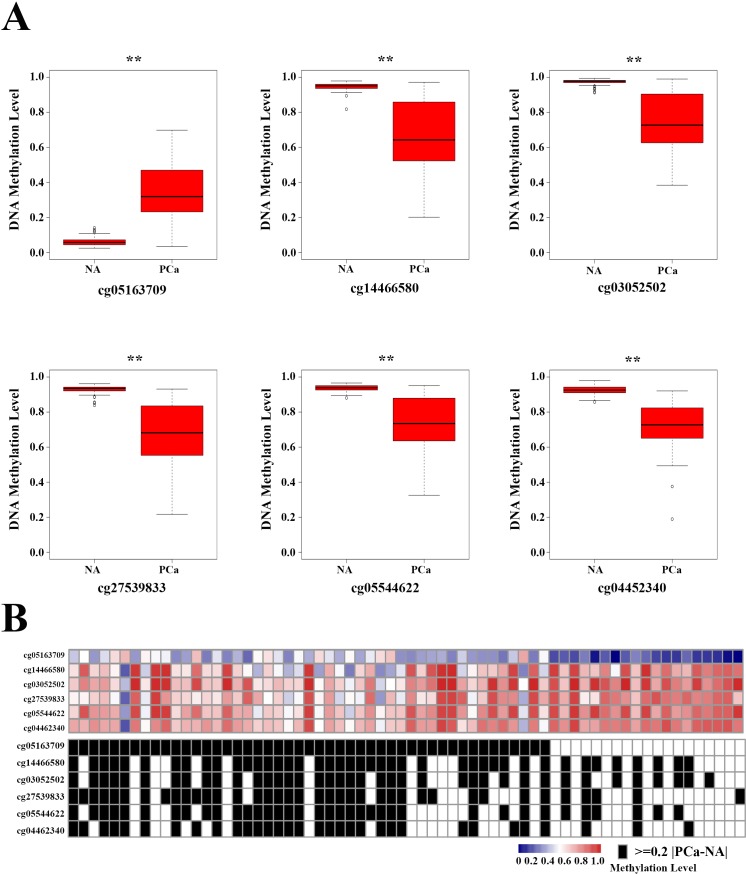
DNA methylated site panel in 66 prostate cancer (PCa) patients **A.** The methylation levels of the six methylated sites in adjacent normal tissues and PCa. **p < 0.01. **B.** Heat map of the selected six methylated sites in PCa. Black boxes in the bottom heatmap indicate PCa patients with obvious differences in the methylated sites (|Δβ-value| ≥ 0.2, *p* < 0.01, FDR-adjusted *p* < 0.01 *vs* controls).

The mean β-value of cg05163709 was higher in PCa than in NA, whereas the mean β-value of other 5 sites were lower in PCa tissues (Figure 6A). All 6 sites had marked differences in most of the cancer tissues compared with their corresponding NA (Figure S3). One or more of the methylated sites in the panel changed remarkably (|Δβ-value | ≥ 0.2) in 57 (86.36%) PCas (Figure 6B). Additionally, the changes in the methylation levels of all 6 sites were not associated with the patients' clinical characteristics (Table 1). These findings strongly suggested that the aberrant methylation levels of these 6 selected sites on the Y-chromosome could be the potential candidate biomarker of PCa.

**Table 1:** Association of DNA methylation level change status and clinical characteristics of 66 prostate cancer patients

Clinical characteristics		All		Cg05163709		Cg14466580		Cg03052502		Cg27539833		Cg05544622		Cg04462340	
	Total	Yes	No	Yes	No	Yes	No	Yes	No	Yes	No	Yes	No	Yes	No
Age, y															
＞65	45	39	6	31	14	28	17	25	20	27	18	24	21	24	21
≤65	15	14	1	13	2	10	5	10	5	9	6	9	6	8	7
PSA, ng/ml															
4-10	21	19	2	15	6	13	8	13	8	16	5	11	10	8	13
＞10	39	33	6	29	10	25	14	22	17	23	16	22	17	22	17
PCa volume,ml															
≤30	36	31	5	28	8	24	12	23	13	24	12	21	15	17	19
30-50	17	15	2	12	5	10	7	8	9	9	8	10	7	10	7
≥50	7	5	2	4	3	4	3	4	3	3	4	2	5	2	5
Gleason score															
<7	3	2	1	0	3	2	1	2	1	1	2	2	1	1	2
=7	34	29	5	25	9	22	12	20	14	20	14	18	16	15	19
＞7	24	22	2	20	4	15	9	14	10	16	8	14	10	15	9
Clinical stage															
I	42	35	7	27	15	28	14	26	16	26	16	26	16	22	20
II	19	18	1	16	3	12	7	11	8	9	10	8	11	7	12
IIII	5	5	0	4	1	2	3	2	3	3	2	2	3	3	2

*Yes means the difference value of the DNA methylation level between prostate cancer tissues and normal adjacent tissues be equal or great than 0.2.

*Staging information was not available for some patients.

### Aberrant methylation of the 6 selected sites is not caused by single-nucleotide polymorphisms (SNPs)

DNA sequence mutations can influence the methylation levels around mutant sites [18, 19]. To investigate whether the changes in the DNA methylation levels of the 6 sites were caused by base pair mutations, we performed whole-genome sequencing of all 66 pairs of PCa tissues and adjacent normal tissues. We found no obvious mutations on the Y-chromosome in PCa tissues, consistent with previous work [20]. We found only 30 pair base mutations on the Y-chromosome in total, and each mutation occurred in only one sample (Table S3). None of the 6 selected methylated sites was mutated in any tissue sample. This result indicated that the genome sequence of the Y-chromosome was stable in PCa and that the aberrant methylation of the 6 sites was not caused by base mutations.

### Aberrant methylation of certain sites could be detected in urine of PCa patients with high specificity and sensitivity

In order to develop non-invasive biomarker for prostate cancer, we next investigated whether the changes in DNA methylation could be detected in urine sample. We selected 3 sites (cg05163709, cg27539833 and cg14466580) to perform this analysis.

We prospectively collected 135 urine sediment samples; the basic clinical and pathological information from these patients was summarized in Table S4. We measured the DNA methylation levels of cg05163709 CpG site through pyrosequencing from 135 (76+59) samples, and cg27539833 CpG site from 76 samples. The methylation level of cg14466580 was not detected.

**Figure 7:**
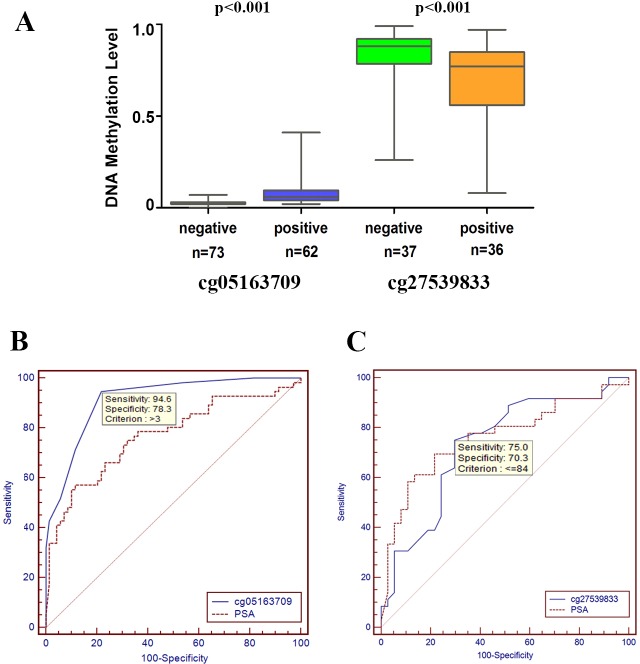
Receiver operating characteristic (ROC) curve analysis for evaluating the diagnostic performance of the methylation levels of cg05163709 and cg27539833 **A.** Comparison of DNA methylation levels of cg05163709 and cg27539833 between positive and negative biopsies. **B.**-**C.** Comparison of the diagnostic performance of the methylation level of cg05163709 (B), the methylation level of cg27539833 (C) and PSA.

As shown in Figure 7A, the methylation levels of cg05163709 and cg27539833 were significantly higher and lower, respectively, in samples with positive biopsy outcomes than in samples with negative results. The areas under the curve (AUCs) were 0.915 for cg05163709 and 0.769 for PSA. A cg05163709 methylation-level cut-off of 3 provided the optimal balance between sensitivity (94.6%) and specificity (78.3%). Additionally, the diagnostic performance of cg05163709 was statistically superior to PSA (Figure 7B). Although the AUC for cg27539833 also reached 0.729, it was not superior to serum PSA alone (Figure 7C). Taken together, these results indicated that the methylation level of cg05163709 could serve as a potential diagnostic biomarker.

## DISCUSSION

Currently, PSA is the most comprehensively used biomarker for PCa. However, the lack of sensitivity and specificity can lead to unnecessary and/or repeat biopsies [21]. Therefore, there is an urgent need for developing more sensitive, specific biomarkers of PCa.

Aberrant DNA methylation could be served as promising cancer biomarker [22–24]. Studies about DNA-based urine biomarkers mostly focused on hypermethylation of gene panels, including GSTP1, RARB and APC, with AUCs of approximately 0.72 [25, 26]. The Y-chromosome is critical for sex determination and sex organ development, including the prostate. Prostate cancer is often accompanied with the loss of Y-chromosome [17]. However, there is no report that links DNA methylation on the Y chromosome to potential biomarkers of PCa, and whether the DNA methylation on the Y-chromosome contributes to PCa development and progression is unclear.

In our current work, we aimed to identify the correlation between PCa and epigenetic modification of the Y-chromosome. First, we performed H3K4me3 ChIP-seq, but found no significant change between cancer tissues and adjacent normal tissues. Then, we focused on the variations in DNA methylation of the Y-chromosome. Compared with adjacent normal tissues, we found 6 methylated sites (Target ID: cg03052502, cg04462340, cg05163709, cg05544622, cg14466580 cg27539893) that were conservative in adjacent normal tissues but changed markedly in tumor tissues. These changes were highly PCa-specific, and were not age or geographically related. Two sites (cg05163709, cg27539893) were selected for further testing by pyrosequencing using PCa patient urine DNA samples. AUC-ROC analysis showed that the methylation level of cg05163709 generated an AUC of 0.915, with high sensitivity and specificity. One advantage of this method is non-invasive because prostate cells can be detected in the urine of men after a digital rectal examination (DRE). The major drawback of our study is the relatively small sample size. Taken together, our results show that the methylation level of urine cg05163709 is a promising diagnostic biomarker, and our work provides the basis for the validation of this novel biomarker in large cohort-based prospective clinical studies.

The incidence of PCa has considerable racial diversity. It would be interesting to test this biomarker in other ethnic populations. In addition, we would investigate the mechanism of these variations of DNA methylation in our future studies. Our work also provides the possibility of identifying and developing DNA methylation-related biomarkers in other types of cancer.

## MATERIALS AND METHODS

### Sample collection and characterization

Sixty-six pairs of cancer and adjacent normal tissues from Chinese patients with PCa were supplied by Shanghai Changhai Hospital. DNA was extracted from the samples using a QIAamp DNA Mini Kit (Cat.No.51306, QIAGEN, Hilden, Germany). All of the urine samples were collected from patients who were scheduled for prostate biopsy because of elevated serum PSA (≥ 4 ng/ml) and/or suspicious DRE in Shanghai Changhai Hospital from August 2014 to December 2014. We also collected their clinical and pathological information. The urine sediment samples were collected as previously reported [27]. DNA was extracted from the urine sediment samples using a QIAamp DNA Mini Kit (Cat.No.51306). We obtained written informed consent from all of the patients. The research protocol was approved by the Ethics Committees of Shanghai Changhai Hospital.

### Data generation

DNA samples were bisulfate converted using a Zymo EZ DNA Methylation Kit (Zymo Research). After the DNA was amplified and enzymatically fragmented, the fragments were purified with ethanol precipitation and hybridized to the Infinium Human Methylation 450 BeadChip array (Illumina Inc., San Diego, CA, USA). After hybridization overnight, extension, staining and washing were performed successively. Eventually, the BeadChips were imaged using an iScan system (Illumina, Inc.).

### Data processing

The raw files were processed for quality control and normalization. Then, the data were analyzed using the official Illumina Methylation Analysis Algorithms. Further we corrected and normalized the deviation caused by different fluorescent markers and probes. Finally, we obtained the methylation level of each tested site as a β-value. β-values ranged from 0 to 1, representing completely unmethylated to fully methylated sites, respectively.

### Statistical analysis of methylation levels of functional regions

The IMA (Illumina Methylation Analyzer) package in R language was used to calculate the methylation level of each methylated site. The methylation level of each site is represented by its ratio with the mean methylation level of the tested sites.

### Statistical analysis of the diagnostic performance of cg05163709 and cg27539833

Wilcoxon rank-sum test or the Mann-Whitney U test was used to compare the continuous variables that were or were not normally distributed, respectively. The Kruskal-Wallis test was applied to compare more than two groups of variables that were not normally distributed. The categorical variables were compared using Pearson's chi-square test. We used the AUC-ROC to evaluate the diagnostic performance of each methylation site. The comparison of the AUCs for different sites was performed using the z-test method [28, 29]. Statistical analysis was performed with MedCalc statistical software version 10.4.7.0 (MedCalc Software bvba, Mariakerke, Belgium).

### Software for data visualization

The ‘heatmap’ package of R was used for hierarchical clustering. The methylation pattern of the Y-chromosome was loaded into IGV for visualization.

### ChIP and ChIP sequencing

ChIP experiments were performed according to the procedure described previously with minimal modification [30]. DNA fragments were purified with the QIAquick PCR purification kit (Qiagen 28106). Sequencing was performed on the Illumina HiSeq 2000 platform. Peaks were called using MACS v1.4.2 [31]. Heatmap counts were generated using seqMINER [32].

### Genome sequencing

Paired-end reads were produced using the Hiseq2000 system. Using the Burrows-Wheeler Alignment tool algorithm, the uniquely alignable reads on NCBI37/hg19 were retained for downstream analysis [33]. SNPs were collected by “mpileup” command line in SAMTools as well as UnifiedGenotyper in GATK [34, 35]. Quality recalibration and local realignment were performed in the GATK pipeline before variation calling.

### Pyrosequencing

Templates used for pyrosequencing were prepared by bisulfite modified DNA. Each PCR was performed in a 40ul volume containing 0.6ul of each primer, 4ul 10XPCR buffer, 0.5ul QIAGEN hotstart Taq, 31.5ul distilled water, and 2.0ul template DNA (treated by bisulfite, EpiTect Bisulfite Kit, Qiagen). Reactions were incubated at 95°C for 3 min, followed by 50 amplification cycles (95°C for 15s, 54°C for 30s, 72°C for 30s), and then a final elongation step at 72°C for 5 min, with the temperature then held at 4°C. Amplicons were confirmed by agarose gel electrophoresis and purified using a QIAquick Gel Extraction Kit (Qiagen ). After mixed with 40ul sequencing buffer (contained 0.5uM sequencing primer), degeneration was performed at 80°C for 2 min. Pyrosequencing was performed using PyroMark ID sequencer.

Primer used in prosequencing.

Cg05163709 Forward: GGAAAGGGGTGATTAAATATTTAGTTA

Reverse: 5′-BIOTIN-CAACCTAATAAAAAACTATACAAACACAT

Sequencing primer: ATAAGTATGTTTAATTATTGTTTAAG

Cg27539833 Forward: GGAATAGTTTAGTTAAAGAAAAAGGTTAAGAT

Reverse: 5′-BIOTIN-AATTTACCACAATACACAAAAAACTAACTACTTA

Sequencing primer: AGATTTTAGTAGTTTTTTGTCGTTA

## SUPPLEMENTARY MATERIAL FIGURES AND TABLES














