# Supplementary material for: Identification of specific DNA methylation sites on the Y-chromosome as biomarker in prostate cancer
Source: Oncotarget. 2015 Oct 16;6(38):40611–21. doi: 10.18632/oncotarget.6141 (PMC4747356; doi:10.18632/oncotarget.6141)
Supplement: Supplementary file 1 [file oncotarget-06-40611-s001.pdf]

Identification of specific DNA methylation sites on the Y-chromosome as biomarker in prostate cancer

Supplementary Material

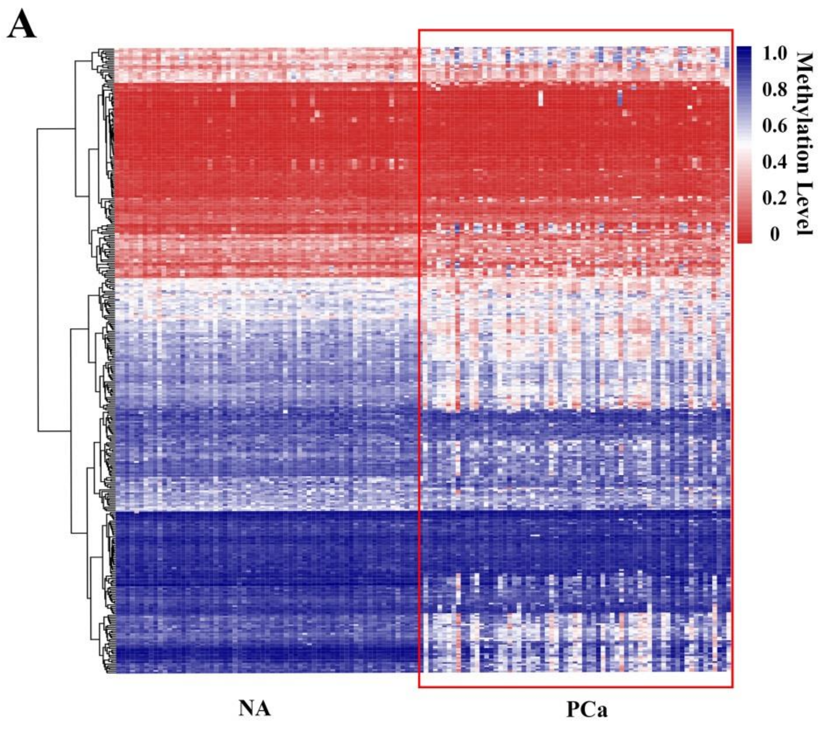

Figure S1. Heat map of the DNA methylation levels of the Y-chromosome in PCa and normal adjacent tissues.

A

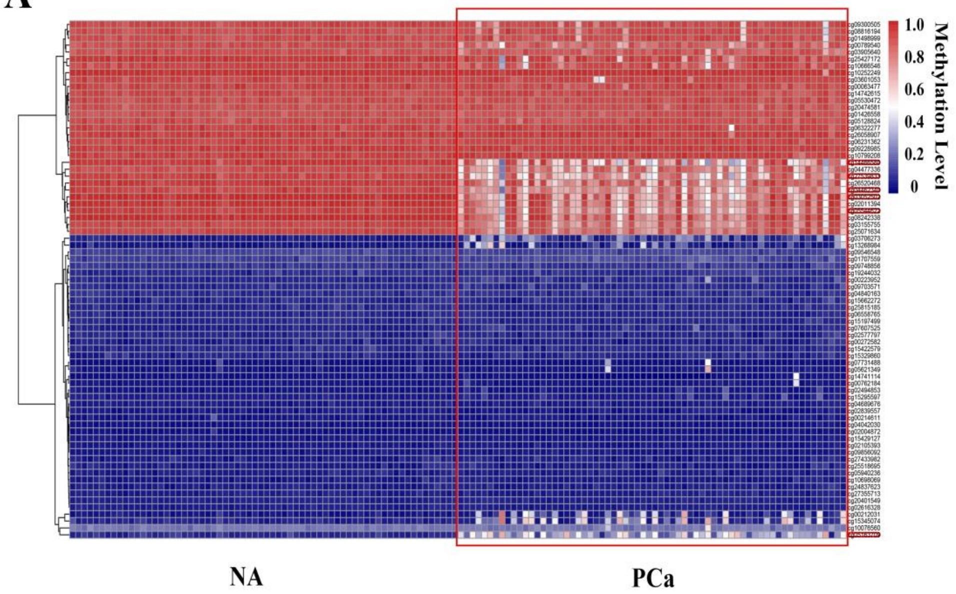

Figure S2. Heat map of DNA methylation levels of the 75 conservative methylated sites on the Y-chromosome in adjacent normal tissues and PCa tissues. Six sites that differed markedly in PCa (|△β-value | ≥ 0.2, $p < 0.01$, FDR-adjusted $p < 0.01$) are marked by red boxes.

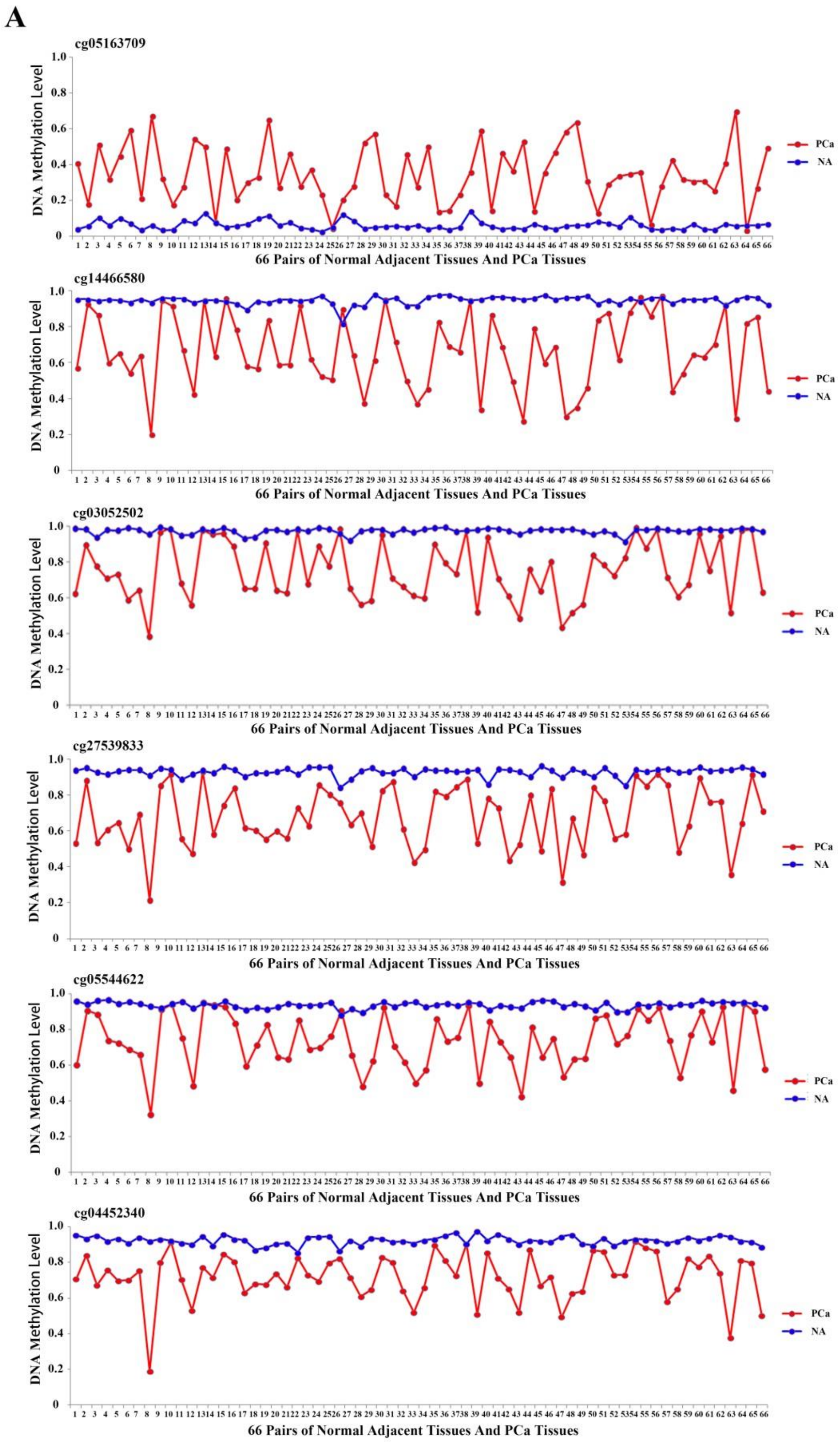

Figure S3. The methylation patterns at cg05163709, cg14466580, cg03052502, cg27539833, cg05544622 and cg04452340 within the 66 pairs of PCa and adjacent normal tissues.

Table S1. Sample Information

Table S2. Detail information of the 37 clear differentiation methylated sites

Table S3. Detective mutation on Y-chromosome

Table S4. Clinical characteristics of the current study cohort
