# Supplementary material for: Identification of specific DNA methylation sites on the Y-chromosome as biomarker in prostate cancer
Source: Oncotarget. 2015 Oct 16;6(38):40611–21. doi: 10.18632/oncotarget.6141 (PMC4747356; doi:10.18632/oncotarget.6141)
Supplement: Supplementary file 3 [file oncotarget-06-40611-s003.pdf]

Table S2: Detail information of the 37 clear differentiation methylated sites

Target ID	P-Value	Adjust Pval	Beta-Difference	Mean_PCa	Mean_NA
cg00050873	1.81E-20	1.29E-18	-0.25099	0.588091	0.839082
cg00213748	5.73E-13	2.96E-12	-0.25861	0.495787	0.754396
cg00639218	3.58E-14	2.09E-13	-0.21337	0.433489	0.646864
cg01053349	2.18E-17	3.02E-16	-0.21901	0.48418	0.703186
cg01828798	7.73E-13	3.77E-12	-0.20812	0.369871	0.577989
cg02107461	2.17E-16	2.08E-15	-0.25113	0.413306	0.664438
cg03052502	3.59E-15	3.21E-14	-0.22197	0.750654	0.972621
cg03443143	3.02E-20	1.53E-18	-0.24604	0.6381	0.88414
cg03515901	1.65E-14	1.03E-13	-0.21875	0.576753	0.795507
cg03535417	1.51E-17	1.92E-16	-0.21705	0.537956	0.755008
cg04169747	3.28E-16	2.92E-15	0.207478	0.471101	0.263622
cg04193779	7.15E-17	7.71E-16	-0.24958	0.582323	0.831906
cg04462340	1.23E-21	2.37E-19	-0.20581	0.718943	0.924757
cg04831594	1.15E-15	8.33E-15	-0.26474	0.486659	0.751395
cg05163709	2.66E-20	1.53E-18	0.284045	0.345909	0.061864
cg05202434	4.27E-16	3.38E-15	-0.20136	0.399125	0.60048
cg05367916	4.03E-19	1.02E-17	-0.20044	0.48617	0.686613
cg05378695	1.05E-15	7.75E-15	-0.20119	0.485708	0.686894
cg05544622	6.31E-18	9.36E-17	-0.20167	0.734588	0.936261
cg05618150	8.13E-20	3.62E-18	0.398152	0.522938	0.124787
cg06587955	1.55E-19	5.03E-18	-0.22887	0.469923	0.698798
cg08053115	7.70E-18	1.05E-16	-0.24167	0.421897	0.663564
cg09197443	2.17E-19	6.43E-18	-0.26902	0.581374	0.850395
cg09350919	2.14E-18	3.81E-17	-0.28899	0.638478	0.927467
cg10363397	3.51E-15	2.32E-14	0.221628	0.697724	0.476096
cg11684211	9.97E-10	3.38E-09	0.252769	0.337722	0.084954
cg11816202	6.83E-06	1.53E-05	0.219845	0.322149	0.102305

cg13419214	3.17E-17	3.53E-16	-0.22683	0.536446	0.763279
cg13654344	8.42E-19	1.67E-17	0.219623	0.515471	0.295847
cg13884608	7.02E-16	2.67E-15	-0.24281	0.584861	0.82767
cg14466580	2.23E-17	2.65E-16	-0.28341	0.663387	0.946797
cg15810474	1.41E-21	2.37E-19	-0.25502	0.488716	0.743734
cg17651935	3.54E-16	3.04E-15	-0.21674	0.656364	0.873103
cg17834650	5.60E-18	8.67E-17	-0.22405	0.591995	0.816048
cg17837162	1.91E-17	2.34E-16	-0.26921	0.565639	0.83485
cg27325772	1.26E-19	4.68E-18	-0.21646	0.412031	0.628489
cg27539833	5.21E-21	4.63E-19	-0.25101	0.67675	0.927762
