# Supplementary material for: Identification of specific DNA methylation sites on the Y-chromosome as biomarker in prostate cancer
Source: Oncotarget. 2015 Oct 16;6(38):40611–21. doi: 10.18632/oncotarget.6141 (PMC4747356; doi:10.18632/oncotarget.6141)
Supplement: Supplementary file 4 [file oncotarget-06-40611-s004.pdf]

Table S3: Detective mutation on Y-chromosome

sample	Chrom	Pos	Ref	Alt	Func	Gene_Symbol	Gene_ID	Strand	ExonicFunc	AAChange
CH17	chrY	16866272	A	G	intron	NLGN4Y	NM_001206850	+		
CH23	chrY	5015786	G	A	intron	PCDH11Y	NM_032973	+		
CH24	chrY	16755955	T	C	intron	NLGN4Y	NM_001164238	+		
CH24	chrY	21897200	G	A	intron	KDM5D	NM_001146706	-		
CH25	chrY	22919727	A	G	intron	RPS4Y2	NM_001039567	+		
CH29	chrY	5491017	C	T	intron	PCDH11Y	NM_032973	+		
CH31	chrY	2816927	T	G	intron	ZFY	NM_003411	+		
CH31	chrY	15027029	T	A	CDS	DDX3Y	NM_004660	+	missense	c.909T>A
CH31	chrY	15027047	G	A	CDS	DDX3Y	NM_004660	+	synonymous	c.927G>A
CH32	chrY	5272877	T	A	intron	PCDH11Y	NM_032973	+		
CH32	chrY	6904106	C	T	intron	TBL1Y	NM_134259	+		
CH34	chrY	14924654	A	T	intron	USP9Y	NM_004654	+		
CH39	chrY	5352819	T	C	intron	PCDH11Y	NM_032973	+		
CH41	chrY	15408022	G	A	intron	UTY	NM_001258267	-		
CH42	chrY	24495863	C	A	intron	RBMY1F	NM_152585	+		
CH49	chrY	5569499	C	G	intron	PCDH11Y	NM_032973	+		
CH50	chrY	15366272	T	C	intron	UTY	NM_001258267	-		
CH50	chrY	16699249	A	G	intron	NLGN4Y	NM_001206850	+		
CH51	chrY	22929411	C	A	intron	RPS4Y2	NM_001039567	+		
CH54	chrY	4913665	C	T	intron	PCDH11Y	NM_032971	+		
CH54	chrY	5350184	G	T	intron	PCDH11Y	NM_032973	+		
CH54	chrY	16863783	C	A	intron	NLGN4Y	NM_001206850	+		
CH57	chrY	16761549	A	G	intron	NLGN4Y	NM_001164238	+		
CH59	chrY	5286262	C	T	intron	PCDH11Y	NM_032973	+		
CH64	chrY	5291896	A	G	intron	PCDH11Y	NM_032973	+		

sample	Chrom	Pos	Ref	Alt	Func	Gene_Symbol	Gene_ID	Strand	ExonicFunc	AAChange
CH72	chrY	5425632	A	T	intron	PCDH11Y	NM_032973	+		
CH73	chrY	5240341	C	A	intron	PCDH11Y	NM_032973	+		
CH77	chrY	5457519	G	C	intron	PCDH11Y	NM_032973	+		
CH78	chrY	15402893	C	G	intron	UTY	NM_001258267	-		
CH78	chrY	16696272	T	C	intron	NLGN4Y	NM_001206850	+		
