# Supplementary material for: Identification of specific DNA methylation sites on the Y-chromosome as biomarker in prostate cancer
Source: Oncotarget. 2015 Oct 16;6(38):40611–21. doi: 10.18632/oncotarget.6141 (PMC4747356; doi:10.18632/oncotarget.6141)
Supplement: Supplementary file 5 [file oncotarget-06-40611-s005.pdf]

Table S4: Clinical characteristics of the current study cohort

Parameter	Entire cohort			
	Negative n=73	Positive n=62	p value	Overall n=135
Age, yr			<0.083*	
No. pts (%)	73(54.1)	62(45.9)		135 (100.0)
Mean±SD	64.6±7.8	66.8±6.8		65.6±7.4
tPSA, ng/ml			<0.001#	
No. pts (%)	73(54.1)	62(45.9)		135 (100.0)
Median[IQR]	9.2[6.6-12.7]	18.1[10.-48.9]		11.3[7.5 - 20.6]
Volume, ml			<0.013#	
No. pts (%)	73(54.1)	62(45.9)		135 (100.0)
Median[IQR]	46.3[31.2-60.2]	37.3[30.1-44.2]		40.6[30.8 - 56.0]
PSAD, ng/ml/cc			<0.001#	
No. pts (%)	73(54.1)	62(45.9)		135 (100.0)
Median[IQR]	0.20[0.13-0.34]	0.62[0.24-1.2]		0.28[0.16 - 0.62]
%fPSA			<0.119#	
No. pts (%)	61(55.5)	49(44.5)		110(100%)
Median[IQR]	0.15[0.11-0.22]	0.12[0.08-0.24]		0.14[0.09--0.22]
Suspicious DRE			0.014§	
No. pts	73(54.1)	62(45.9)		135 (100.0)
No. %	19(26.0)	21(33.9)		40(22.2)
cg 05163709			<0.001#	
No. pts (%)	69(55.2)	56(44.8)		125(100.0)
Median[IQR]	3[2.0-3.0]	6[4.0-9.0]		4[2-6]
cg27539833			<0.001#	
No. pts (%)	37(48.7)	36(33.2)		73(100.0)
Median[IQR]	88[80.3-92.0]	77[56.0-84.5]		84[71.0-90.3]
Biopsy cores				
No. pts (%)	73(54.1)	62(45.9)		135(100.0)
Median[IQR]	12[12-12]	12[12-12]		12[12-12]
Positive cores, no.				
Median[IQR]		4.5[2.0-8.0]		
Biopsy Gleason score, no. (%)				
≤ 6		19(30.65)		
7		19(30.65)		
≥ 8		24(38.7)		

Abbreviations: PSA: prostate-specific antigen; SD: standard deviation; IQR: interquartile range; tPSA: total PSA; PSAD: PSA density (serum PSA/prostate volume); %fPSA: percent free PSA; DRE: digital rectal examination.

*Wilcoxon rank-sum Test. #Mann-Whitney U test. §Pearson's Chi-square test
