# Supplementary material for: Identification of specific DNA methylation sites on the Y-chromosome as biomarker in prostate cancer
Source: Oncotarget. 2015 Oct 16;6(38):40611–21. doi: 10.18632/oncotarget.6141 (PMC4747356; doi:10.18632/oncotarget.6141)

| Sample ID. | Sample | Pathology | Sample ID. | Sample | Pathology |
|---|---|---|---|---|---|
| 5 | 841544-5 | BPH | 48 | 911148 | PCa |
| 6 | 914889-1 | BPH | 49 | 906518 | PCa |
| 7 | 915438-4 | BPH | 50 | 913192 | PCa |
| 8 | 915085-2 | BPH | 51 | 907942 | PCa |
| 9 | 652150-6 | BPH | 52 | 589342 | PCa |
| 11 | 915418-3 | BPH | 53 | 905407 | PCa |
| 12 | 914884-7 | BPH | 54 | 709085 | PCa |
| 13 | 914733-8 | BPH | 55 | 908370 | PCa |
| 17 | 909973 | BPH | 103 | 903823 | PCa |
| 18 | 914356 | BPH | 111 | 897442 | PCa |
| 19 | 913694 | BPH | 112 | 901045 | PCa |
| 20 | 903268 | BPH | 113 | 901043 | PCa |
| 21 | 908210 | BPH | 114 | 902018 | PCa |
| 22 | 910531 | BPH | 136 | 791724 | PCa |
| 23 | 908682 | BPH | 150 | 887515 | PCa |
| 24 | 907033 | BPH | 151 | 885709 | PCa |
| 56 | 912473 | BPH | 152 | 889652 | PCa |
| 57 | 910231 | BPH | 154 | 888371 | PCa |
| 58 | 912391 | BPH | 161 | 883873 | PCa |
| 59 | 910991 | BPH | 165 | 883888 | PCa |
| 60 | 908362 | BPH | 166 | 880629 | PCa |
| 61 | 914729 | BPH | 167 | 883874 | PCa |
| 62 | 757418 | BPH | 178 | 887518 | PCa |
| 63 | 909223 | BPH | 183 | 916479 | PCa |
| 64 | 912354 | BPH | 185 | 916137 | PCa |
| 65 | 907679 | BPH | 189 | 916480 | PCa |
| 66 | 908168 | BPH | 191 | 916826 | PCa |
| 67 | 907031 | BPH | 194 | 917004 | PCa |
| 68 | 776982 | BPH | 195 | 917172 | PCa |
| 69 | 905091 | BPH | 204 | 917725 | PCa |
| 70 | K01556 | BPH | 208 | 917520 | PCa |
| 71 | 910227 | BPH | 101 | 903813 | BPH |
| 72 | 476366 | BPH | 102 | 903826 | BPH |
| 73 | 613622 | BPH | 116 | 462720 | BPH |
| 74 | 908502 | BPH | 118 | 906867 | BPH |
| 75 | 911632 | BPH | 120 | 905955 | BPH |
| 76 | 911642 | BPH | 127 | 905247 | BPH |
| 77 | 908384 | BPH | 128 | 904835 | BPH |
| 1 | 914012-16 | PCa | 129 | 904040 | BPH |
| 2 | 914352-14 | PCa | 130 | 904035 | BPH |
| 3 | 915077-11 | PCa | 135 | 897251 | BPH |
| 4 | 914886-12 | PCa | 139 | 895260 | BPH |
| 10 | 912058-10 | PCa | 147 | 885889 | BPH |
| 14 | 915421-9 | PCa | 148 | 887357 | BPH |
| 15 | 914727-13 | PCa | 155 | 889442 | BPH |
| 16 | 913196-15 | PCa | 156 | 889796 | BPH |
| 25 | 904034 | PCa | 158 | 888377 | BPH |
| 26 | 910527 | PCa | 159 | 889068 | BPH |
| 27 | 906349 | PCa | 160 | 867509 | BPH |
| 28 | 912589 | PCa | 162 | 803101 | BPH |
| 29 | 913504 | PCa | 164 | 887506 | BPH |
| 30 | 911847 | PCa | 168 | 882798 | BPH |
| 31 | 447800 | PCa | 169 | 881699 | BPH |
| 32 | 913844 | PCa | 172 | 884431 | BPH |

| | | | | | |
|---|---|---|---|---|---|
| 34 | 907486 | PCa | 173 | 885527 | BPH |
| 35 | 903164 | PCa | 177 | 880068 | BPH |
| 36 | 908515 | PCa | 181 | 881507 | BPH |
| 37 | 906318 | PCa | 182 | 916416 | BPH |
| 38 | 903322 | PCa | 184 | 916135 | BPH |
| 39 | 908161 | PCa | 186 | 915632 | BPH |
| 40 | 904834 | PCa | 190 | 916477 | BPH |
| 41 | 905716 | PCa | 192 | 916378 | BPH |
| 42 | 912730 | PCa | 196 | 916682 | BPH |
| 43 | 913288 | PCa | 197 | 726393 | BPH |
| 44 | 907035 | PCa | 198 | 917176 | BPH |
| 45 | 908033 | PCa | 199 | 917739 | BPH |
| 46 | 904211 | PCa | 201 | 917542 | BPH |
| 47 | 913289 | PCa | | | |

# Cg05163709

Assay Name: CZ-1
Sample ID: 5
Note:

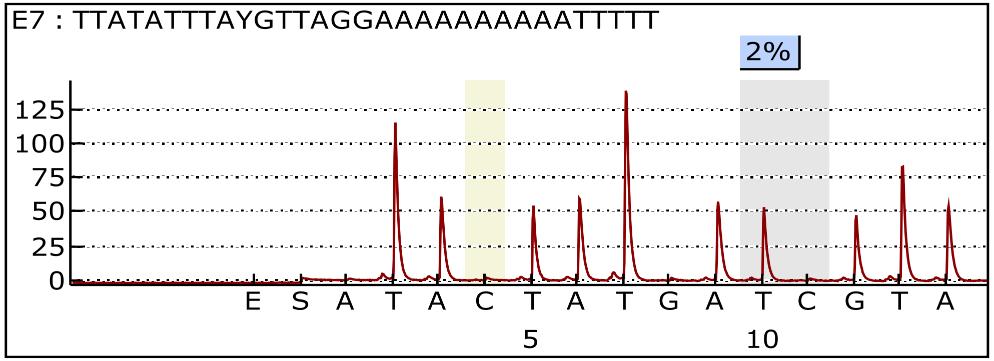


Assay Name: CZ-1
Sample ID: 6
Note:

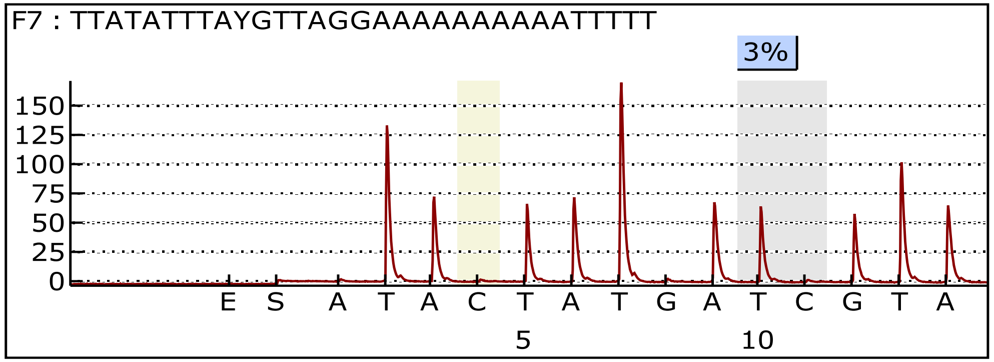


Assay Name: CZ-1
Sample ID: 7
Note:

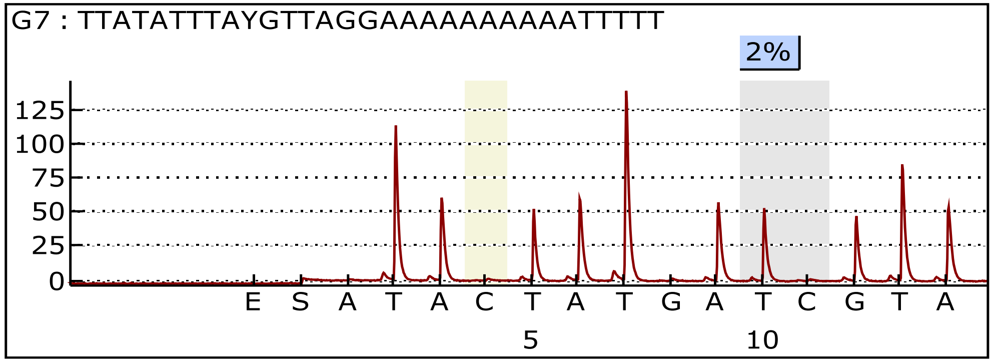


Assay Name: CZ-1
Sample ID: 8
Note:

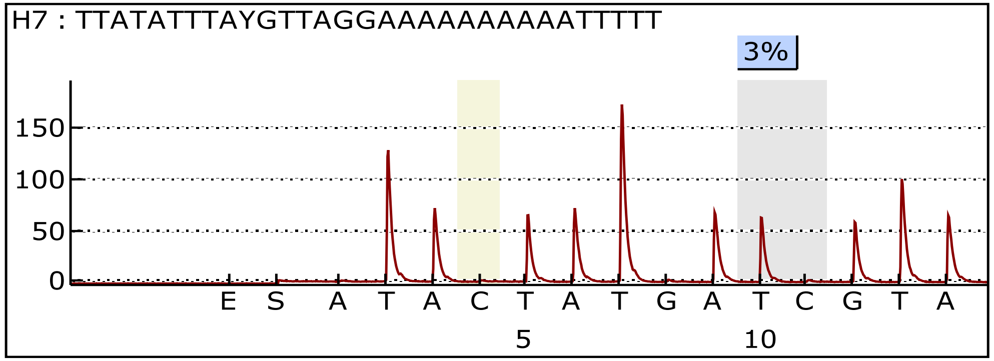


Assay Name: CZ-1
Sample ID: 9
Note:

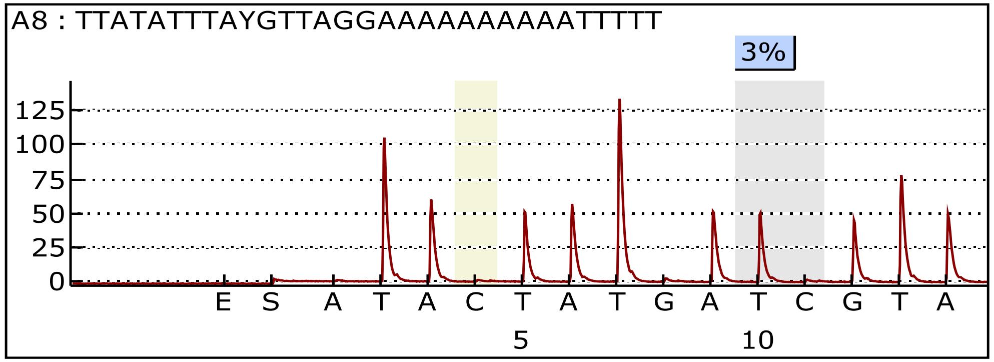


Assay Name: CZ-1
Sample ID: 11
Note:

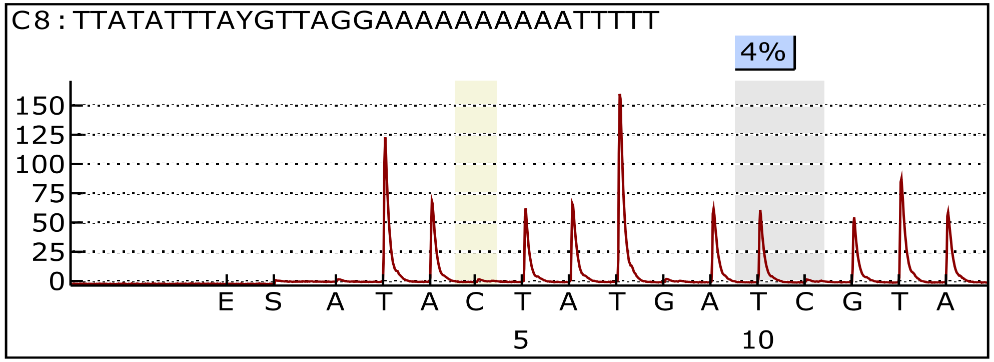

Assay Name: CZ-1
Sample ID: 12
Note:

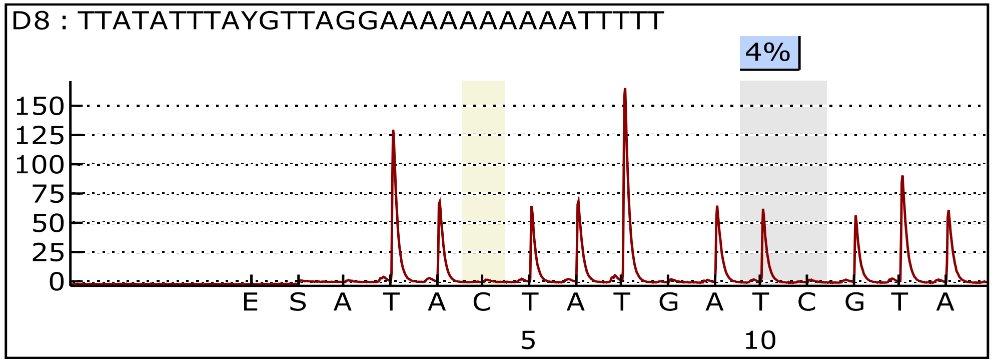


Assay Name: CZ-1
Sample ID: 18
Note:

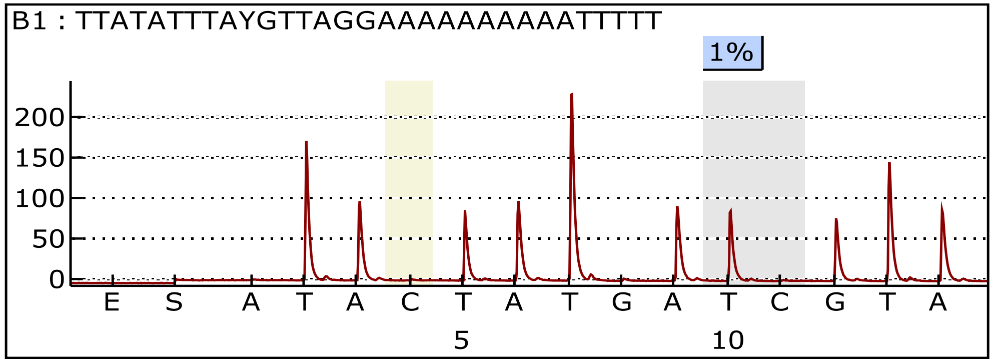


Assay Name: CZ-1
Sample ID: 13
Note:

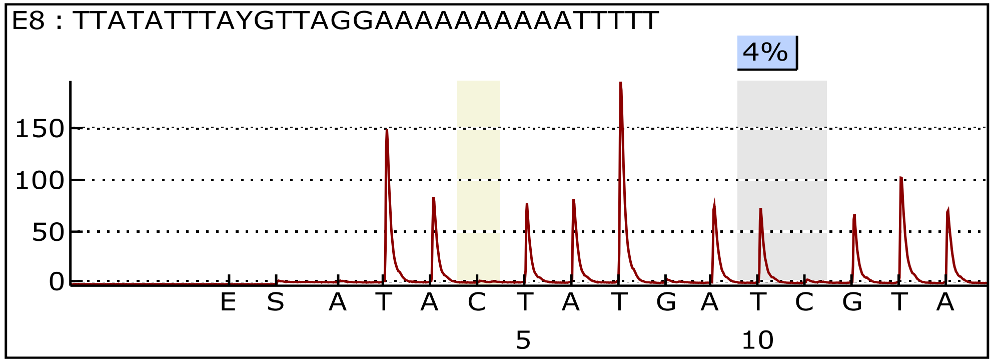


Assay Name: CZ-1
Sample ID: 19
Note:

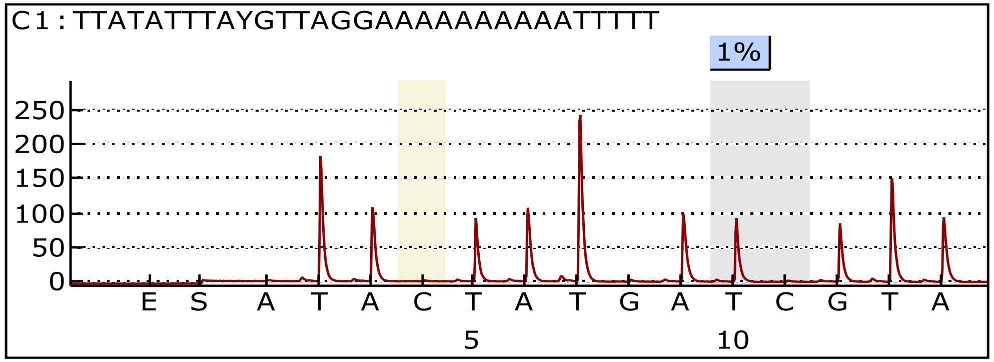


Assay Name: CZ-1
Sample ID: 17
Note:

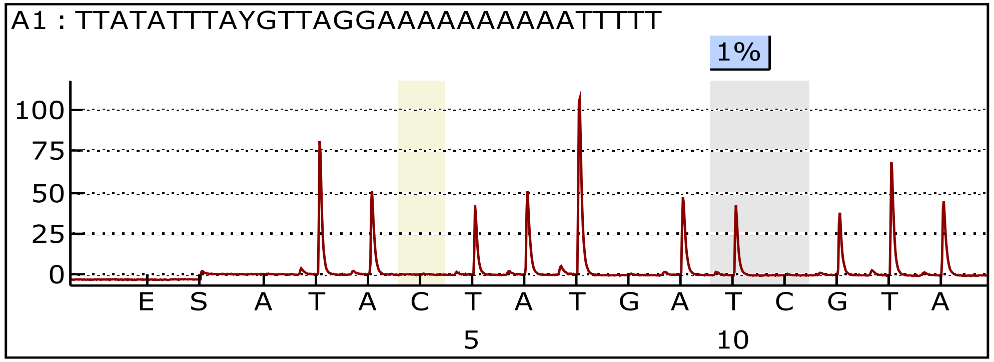


Assay Name: CZ-1
Sample ID: 20
Note:

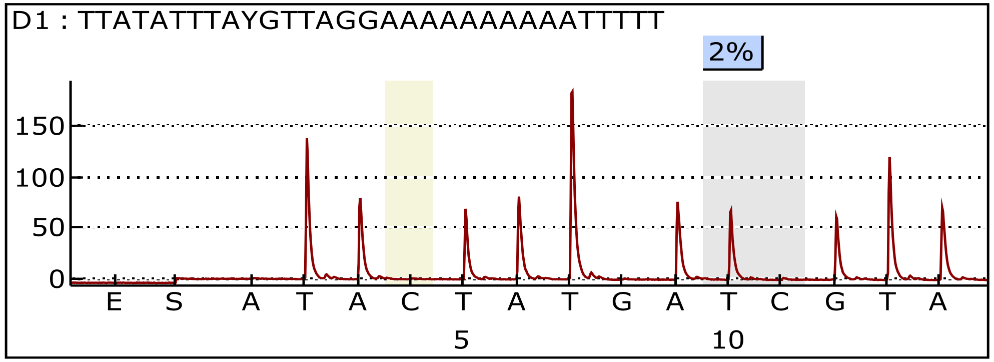

Assay Name: CZ-1
Sample ID: 21
Note:

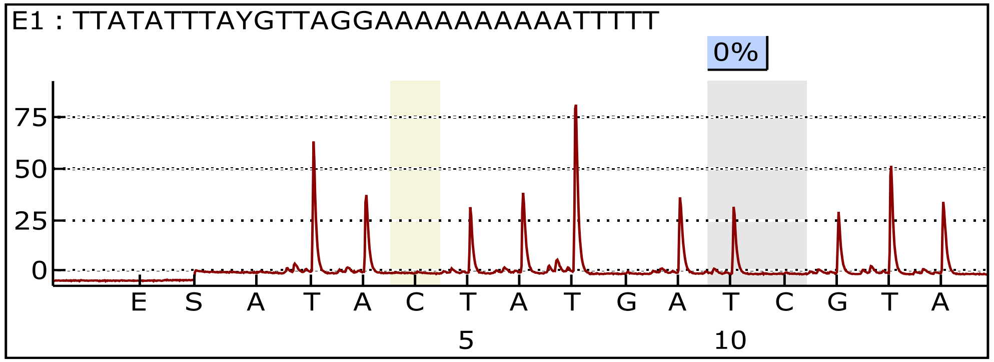


Assay Name: CZ-1
Sample ID: 22
Note:

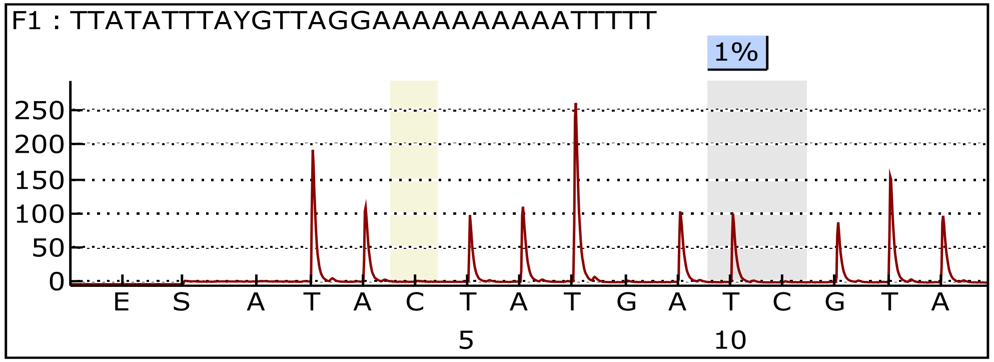


Assay Name: CZ-1
Sample ID: 23
Note:

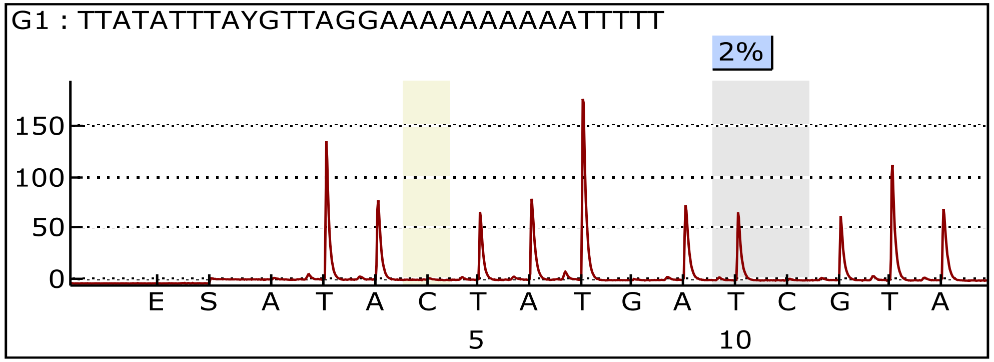


Assay Name: CZ-1
Sample ID: 24
Note:

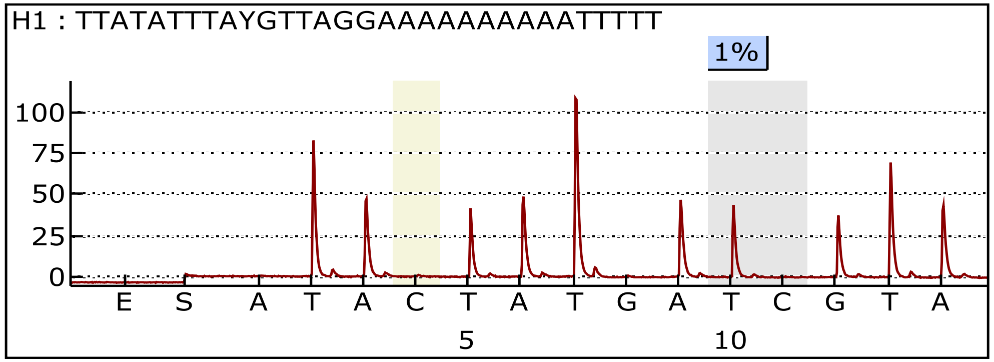


Assay Name: CZ-1
Sample ID: 56
Note:

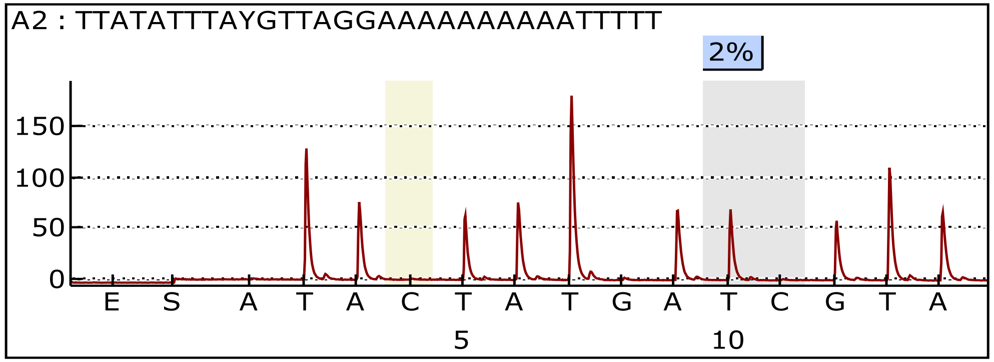


Assay Name: CZ-1
Sample ID: 57
Note:

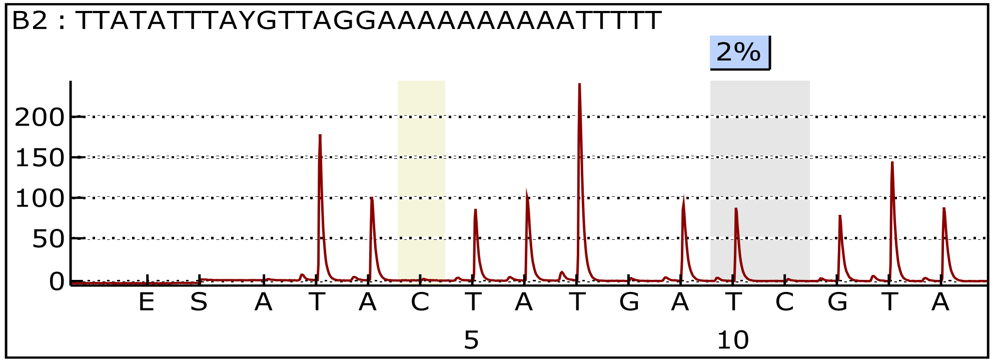

Assay Name: CZ-1
Sample ID: 58
Note:

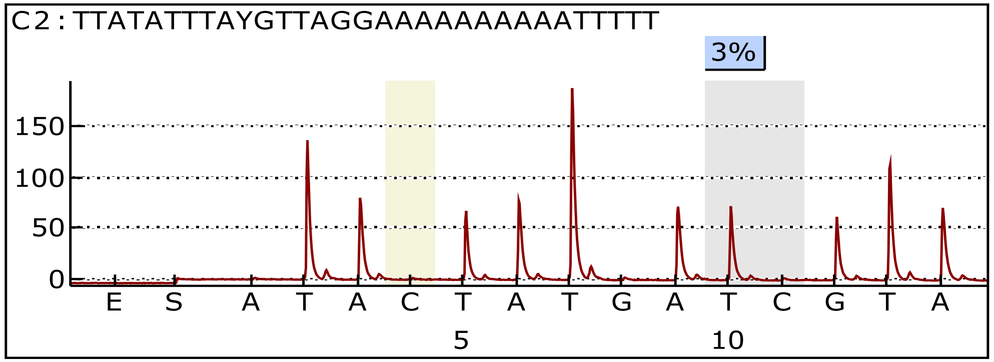


Assay Name: CZ-1
Sample ID: 61
Note:

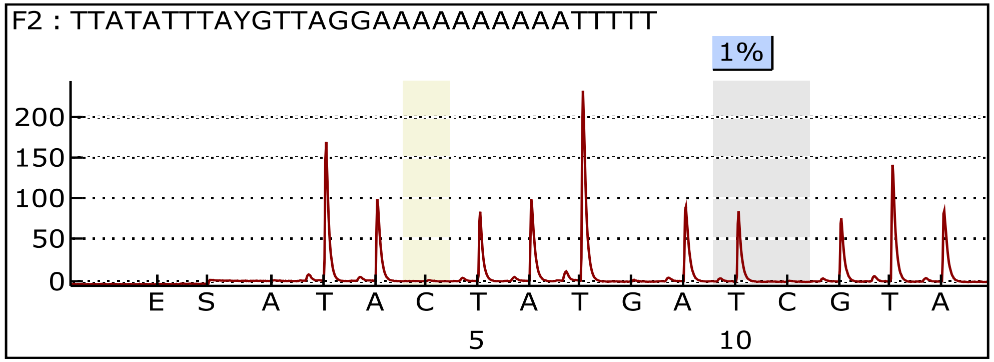


Assay Name: CZ-1
Sample ID: 59
Note:

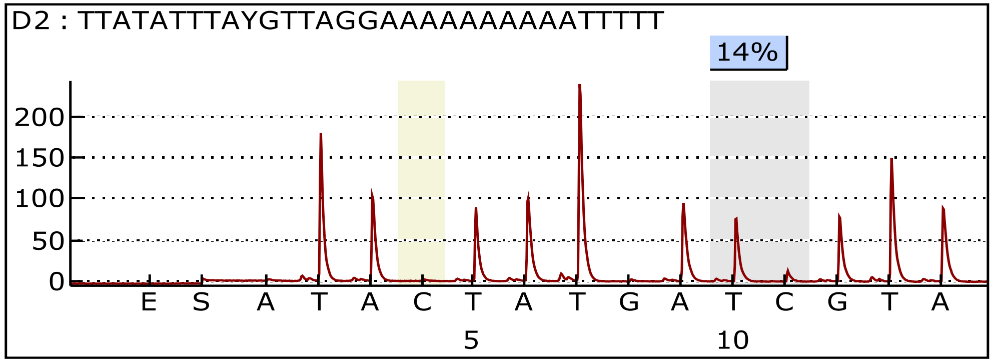


Assay Name: CZ-1
Sample ID: 62
Note:

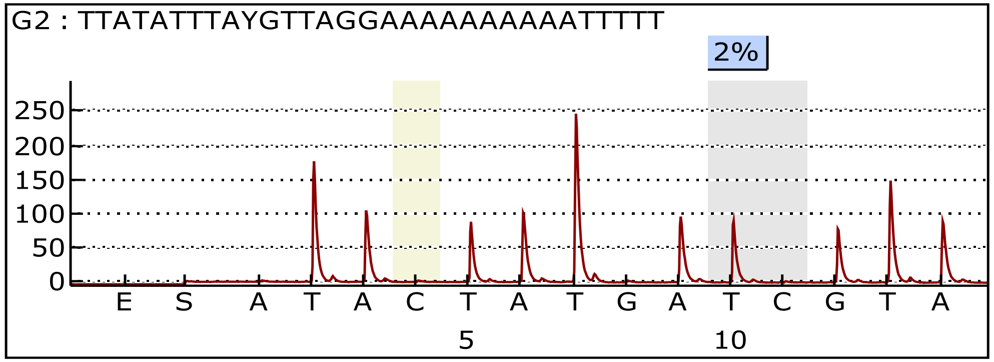


Assay Name: CZ-1
Sample ID: 60
Note:

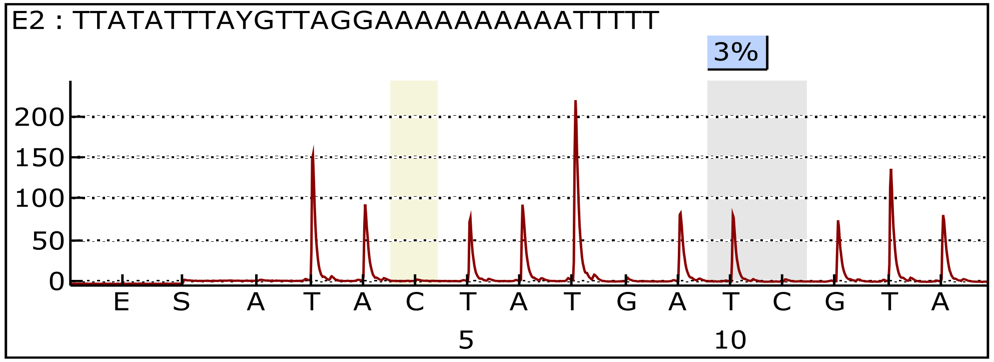


Assay Name: CZ-1
Sample ID: 63
Note:

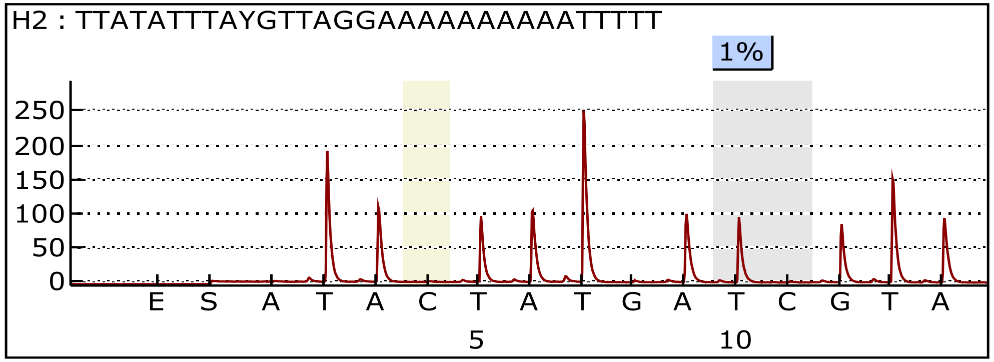

Assay Name: CZ-1
Sample ID: 64
Note:

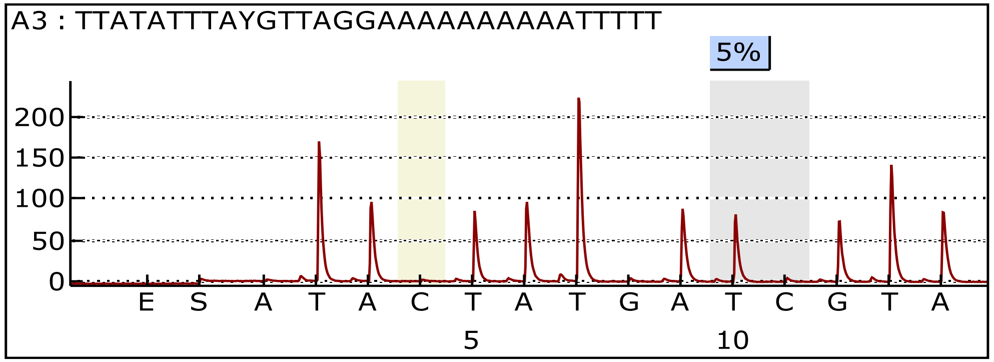


Assay Name: CZ-1
Sample ID: 65
Note:

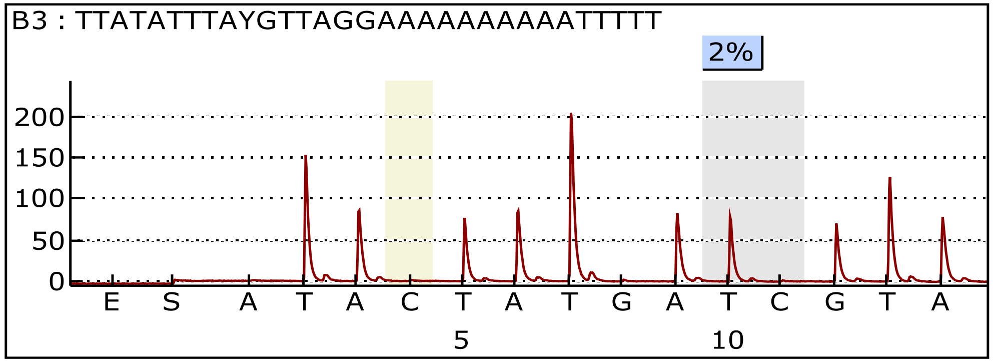


Assay Name: CZ-1
Sample ID: 66
Note:

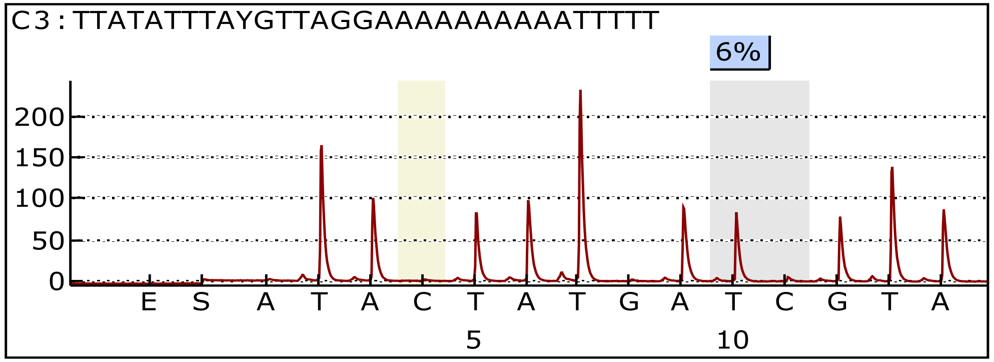


Assay Name: CZ-1
Sample ID: 67
Note:

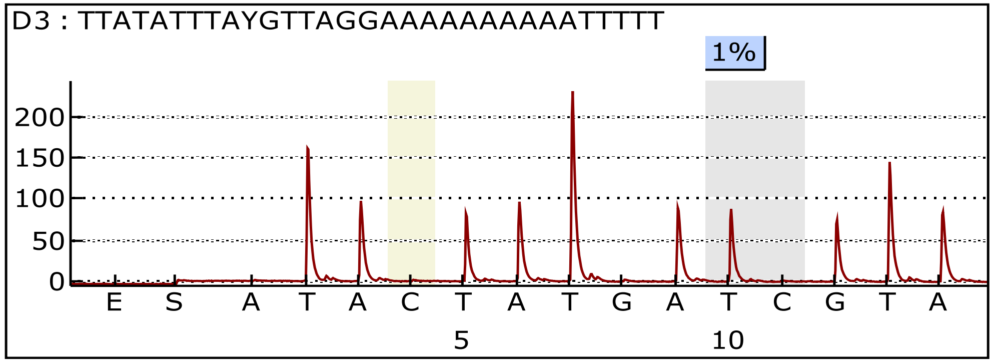


Assay Name: CZ-1
Sample ID: 68
Note:

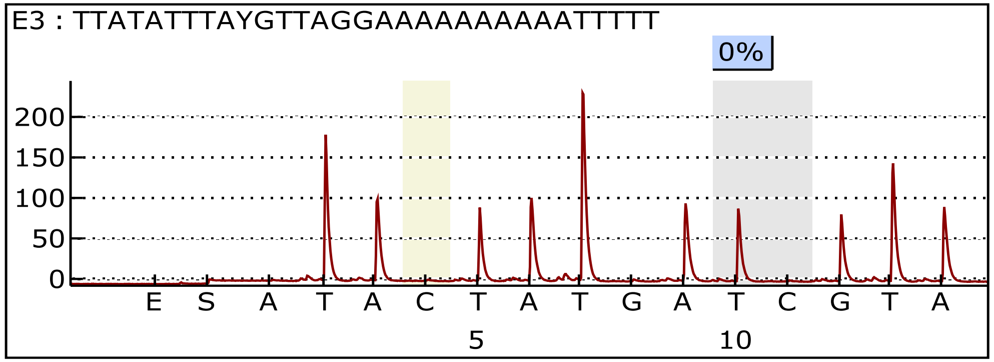


Assay Name: CZ-1
Sample ID: 69
Note:

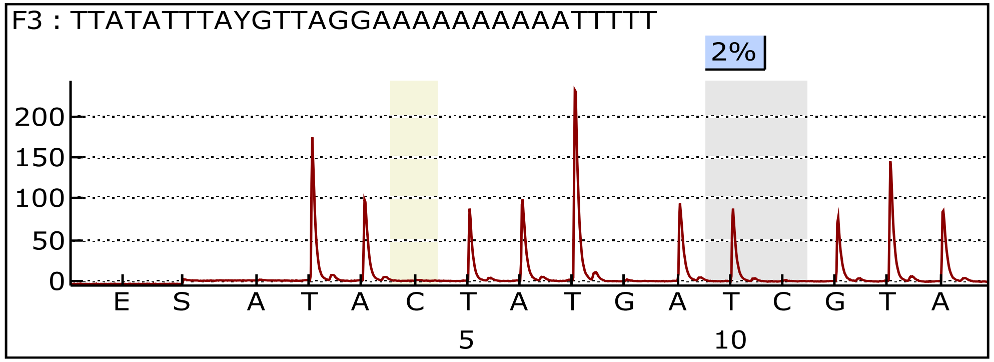

Assay Name: CZ-1
Sample ID: 70
Note:

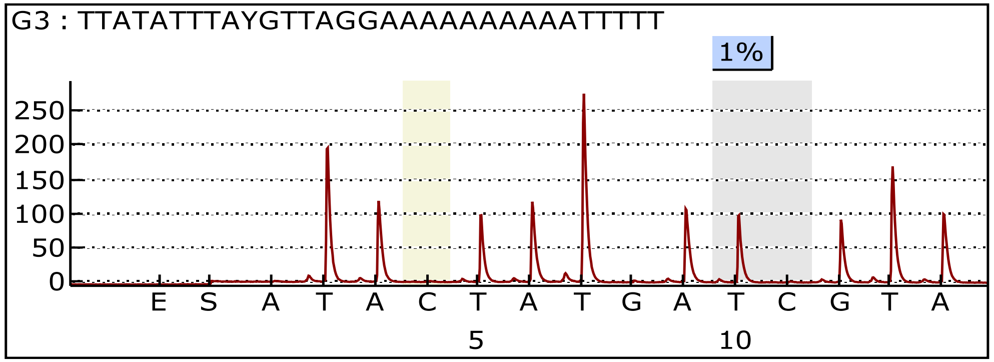


Assay Name: CZ-1
Sample ID: 71
Note:

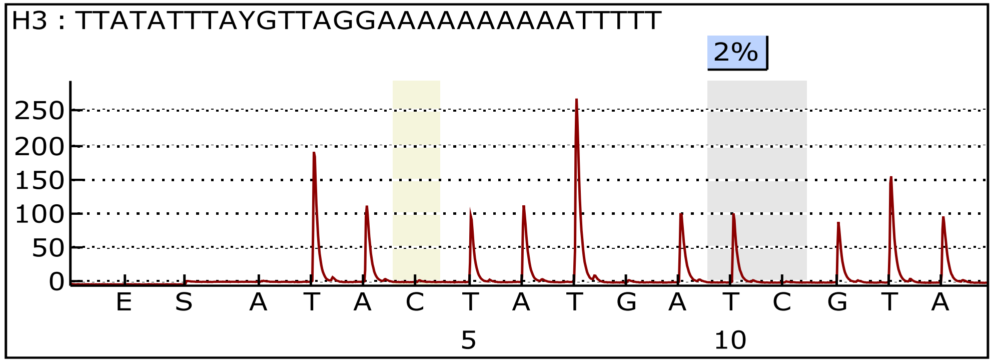


Assay Name: CZ-1
Sample ID: 72
Note:

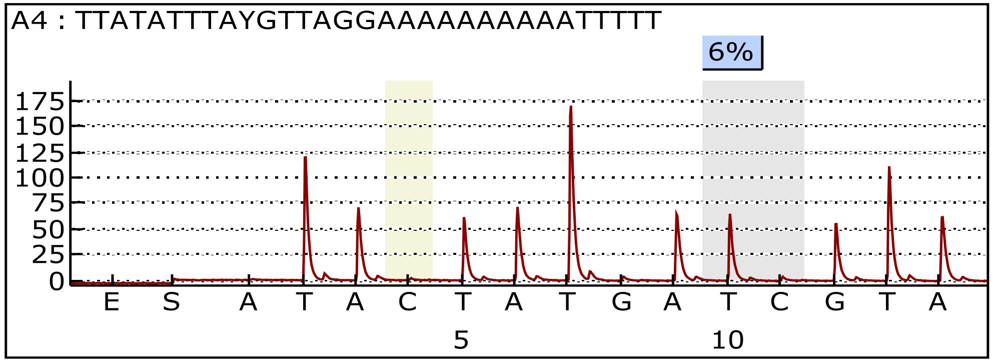


Assay Name: CZ-1
Sample ID: 73
Note:

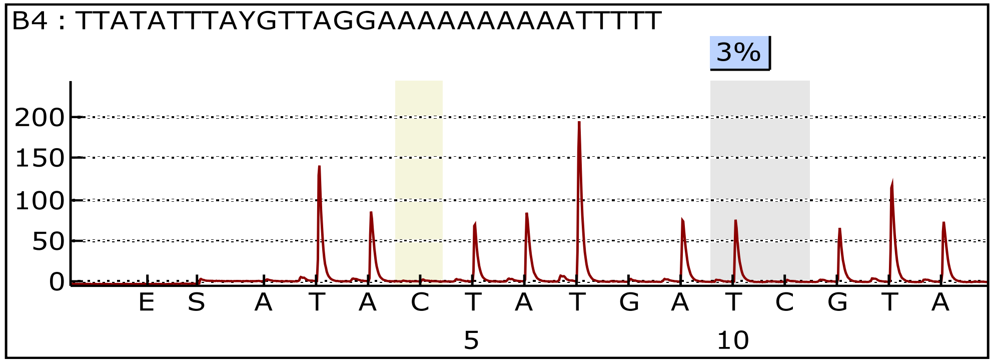


Assay Name: CZ-1
Sample ID: 74
Note:

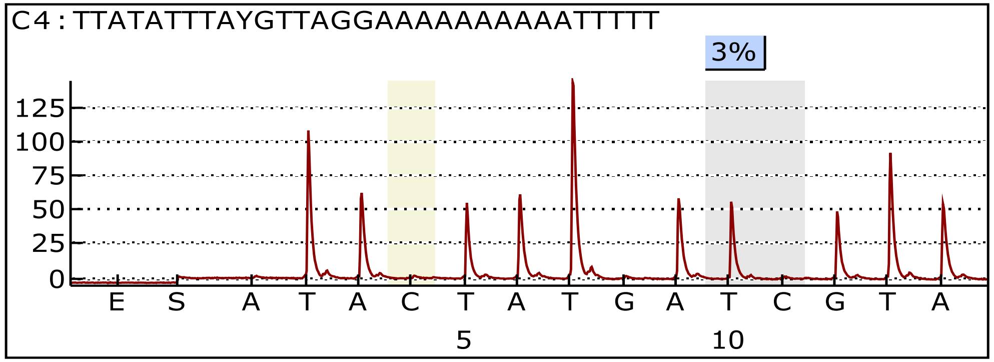


Assay Name: CZ-1
Sample ID: 75
Note:

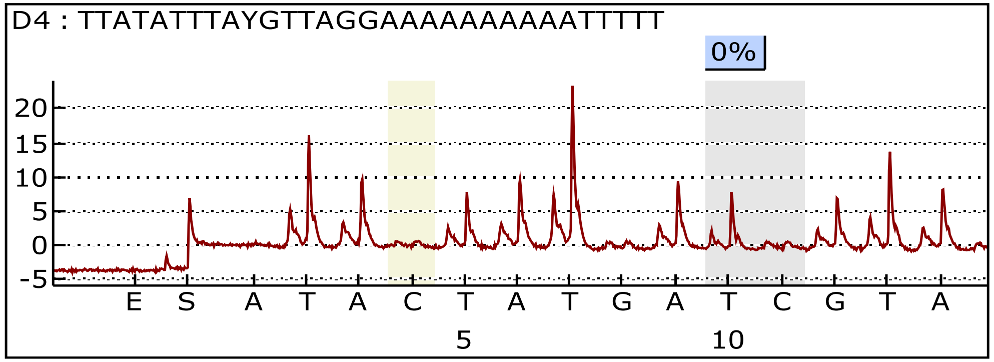

Assay Name: CZ-1
Sample ID: 76
Note:

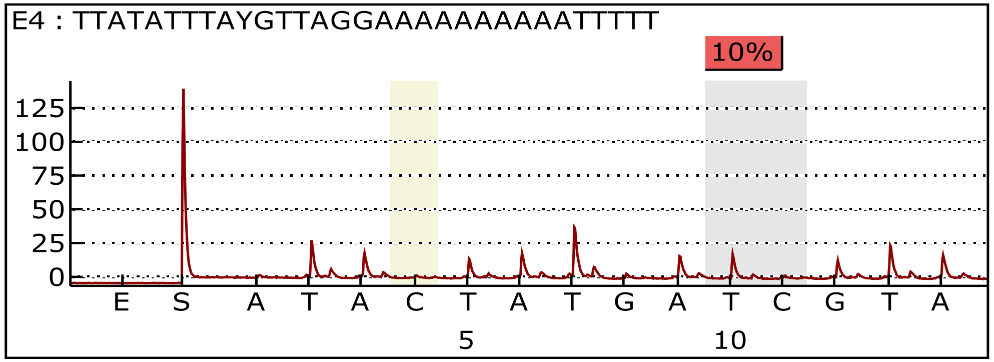


Assay Name: CZ-1
Sample ID: 2
Note:

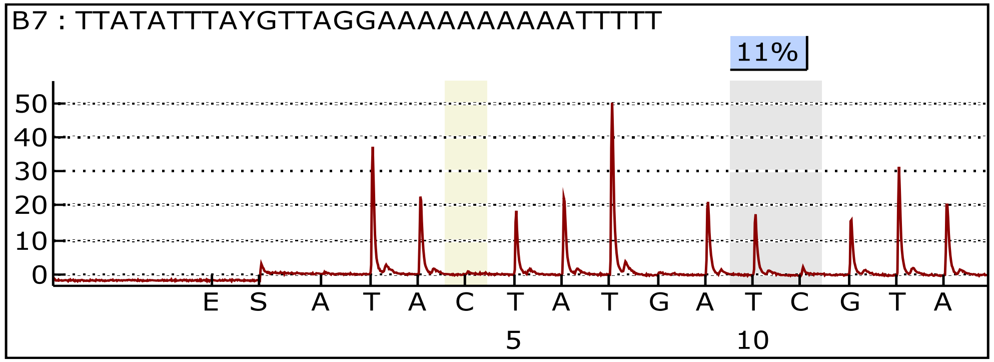


Assay Name: CZ-1
Sample ID: 77
Note:

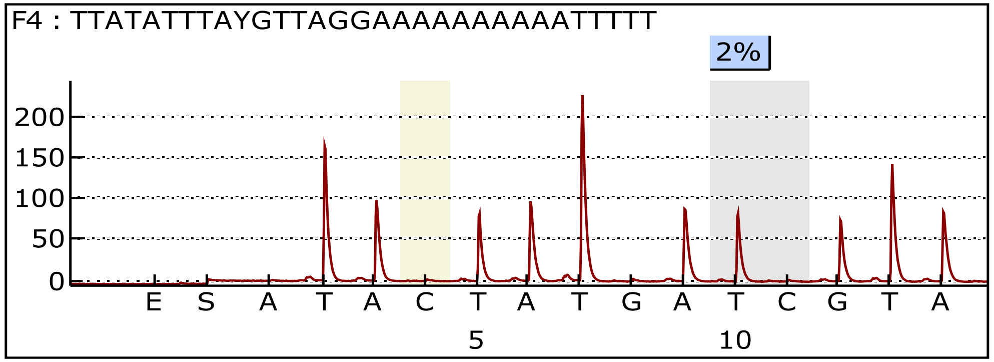


Assay Name: CZ-1
Sample ID: 3
Note:

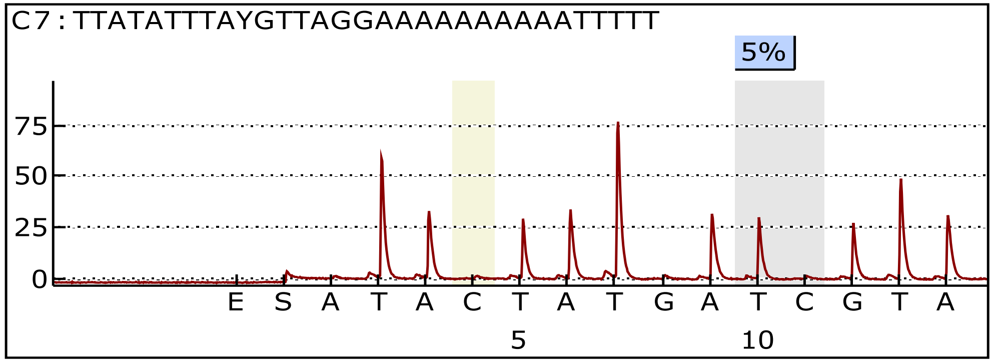


Assay Name: CZ-1
Sample ID: 1
Note:

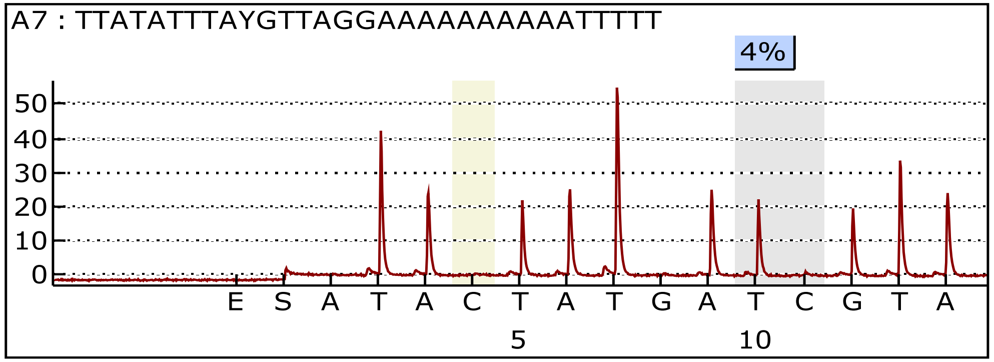


Assay Name: CZ-1
Sample ID: 4
Note:

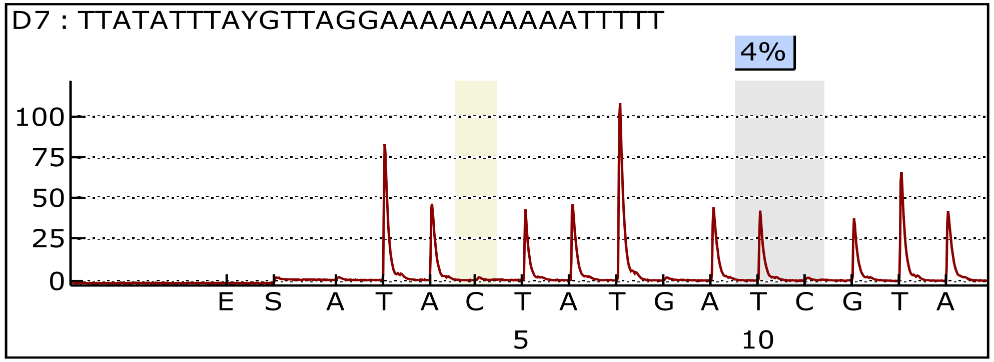

Assay Name: CZ-1
Sample ID: 10
Note:

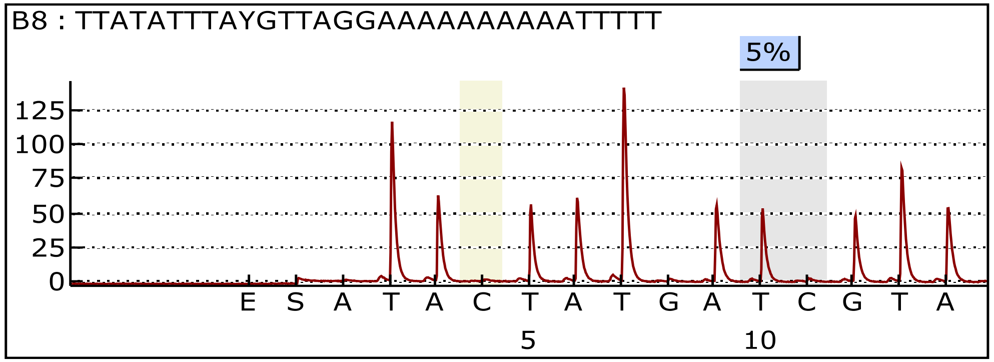


Assay Name: CZ-1
Sample ID: 16
Note:

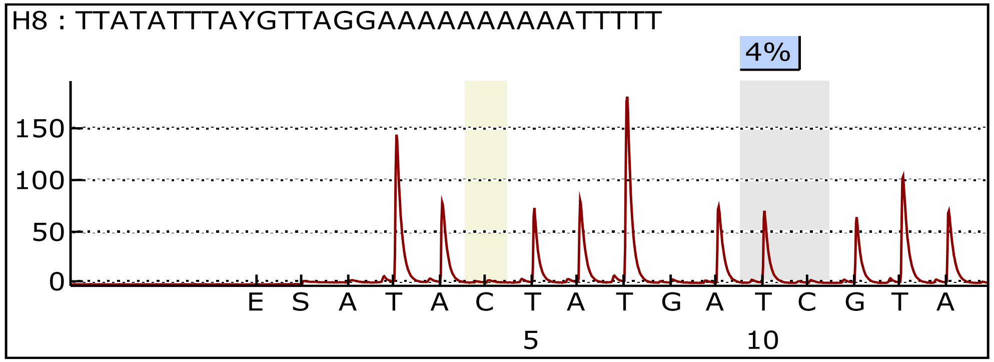


Assay Name: CZ-1
Sample ID: 14
Note:

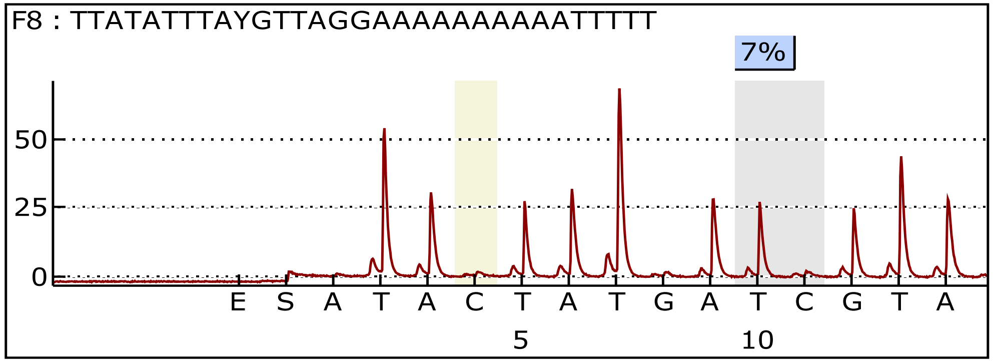


Assay Name: CZ-1
Sample ID: 25
Note:

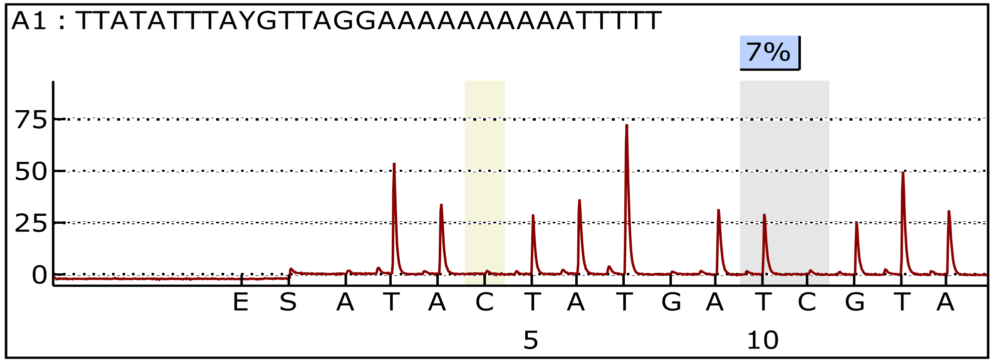


Assay Name: CZ-1
Sample ID: 15
Note:

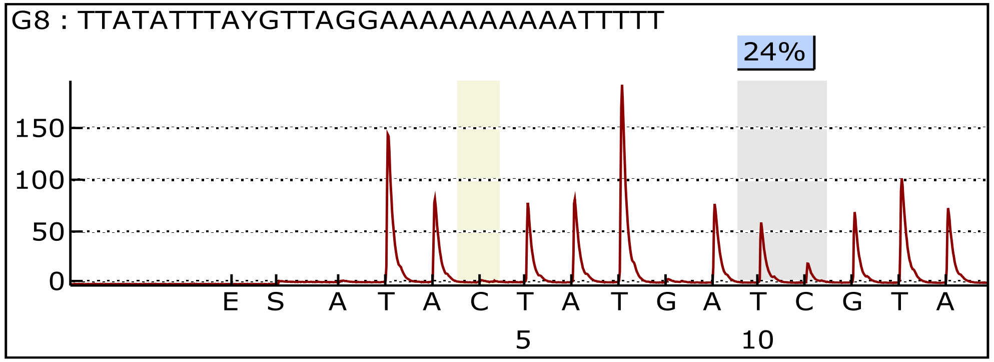


Assay Name: CZ-1
Sample ID: 26
Note:

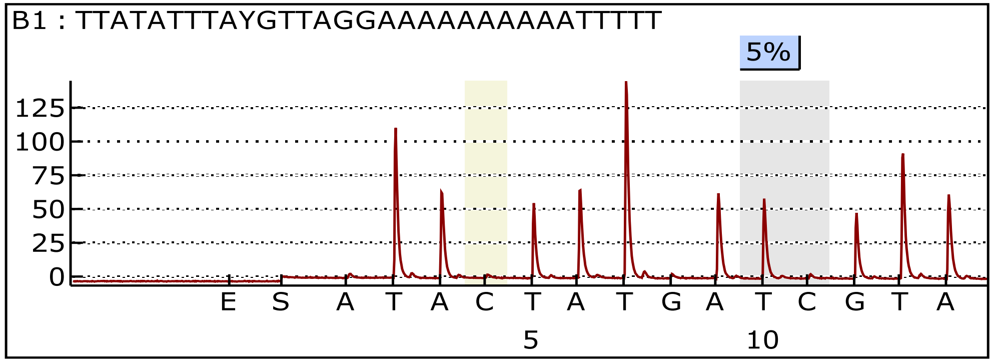

Assay Name: CZ-1
Sample ID: 27
Note:

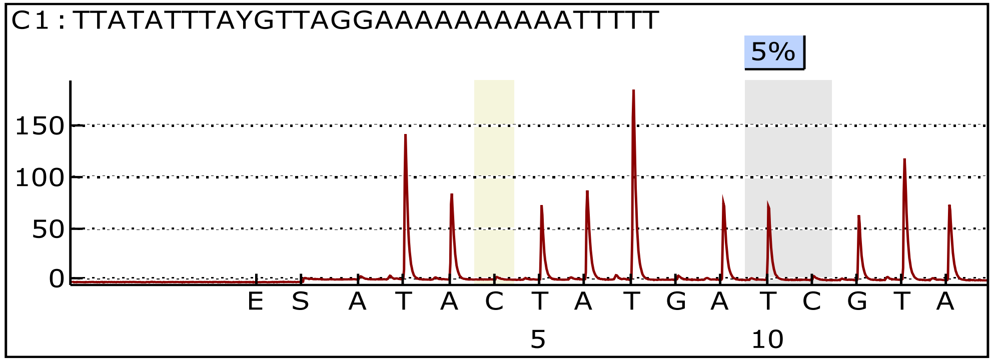


Assay Name: CZ-1
Sample ID: 28
Note:

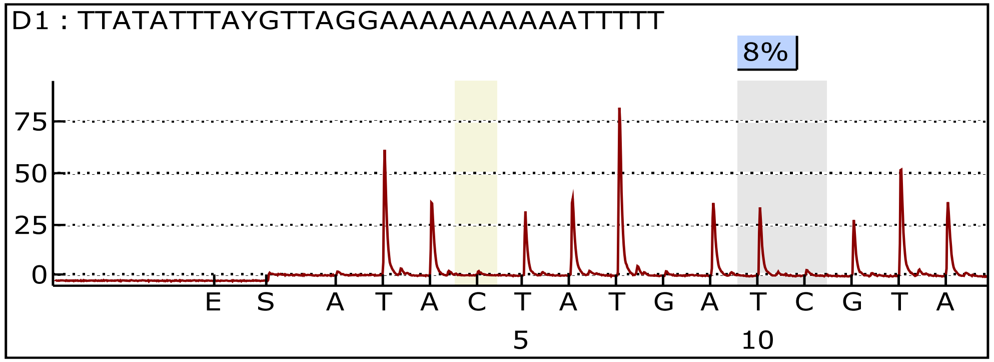


Assay Name: CZ-1
Sample ID: 29
Note:

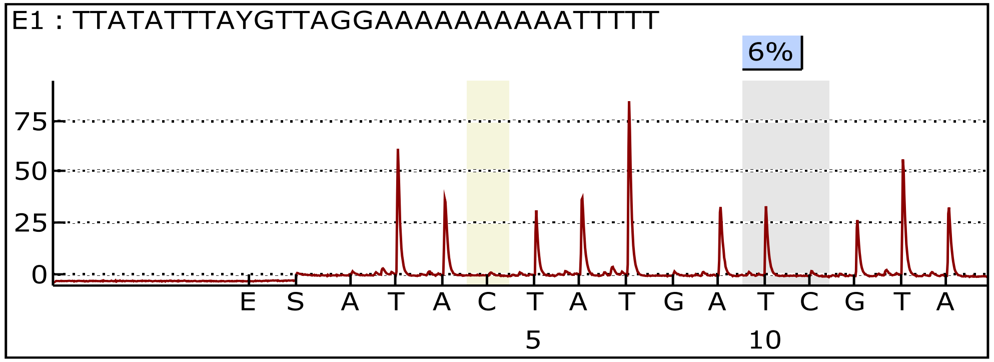


Assay Name: CZ-1
Sample ID: 30
Note:

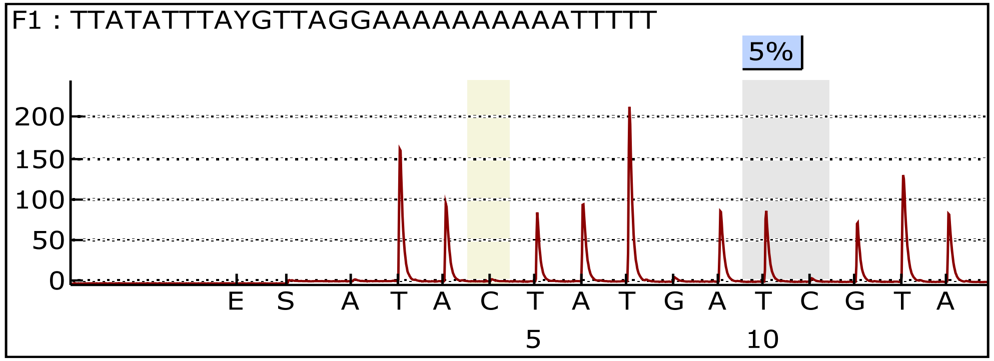


Assay Name: CZ-1
Sample ID: 31
Note:

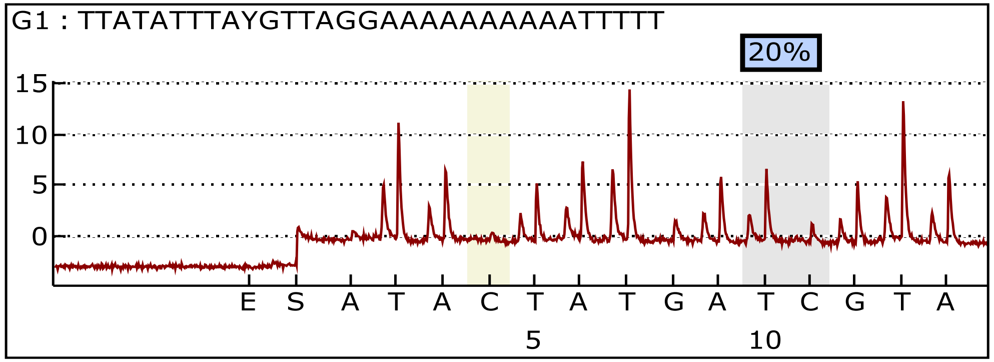


Assay Name: CZ-1
Sample ID: 32
Note:

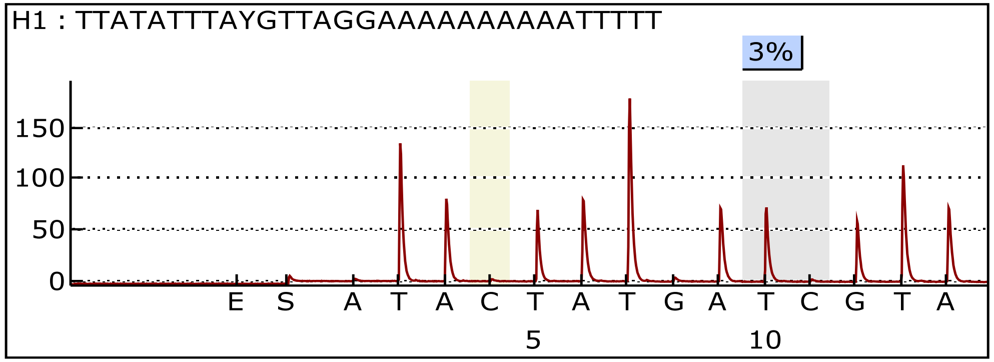

Assay Name: CZ-1
Sample ID: 34
Note:

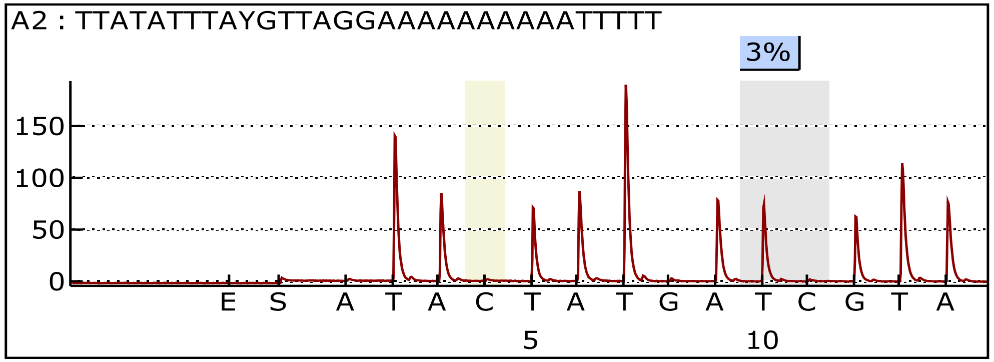


Assay Name: CZ-1
Sample ID: 35
Note:

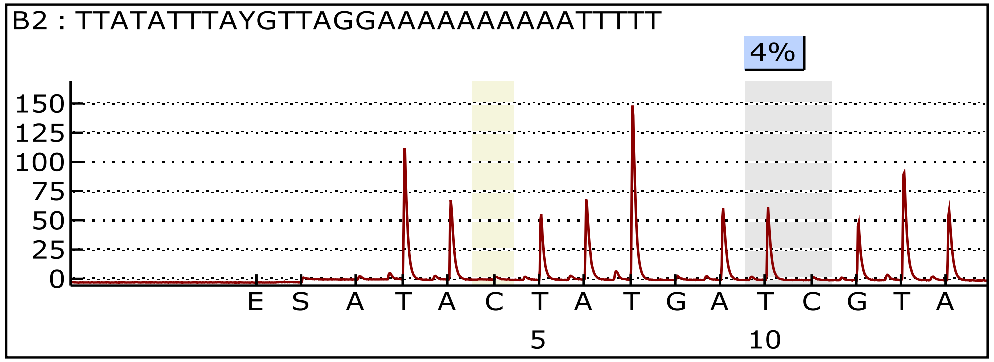


Assay Name: CZ-1
Sample ID: 36
Note:

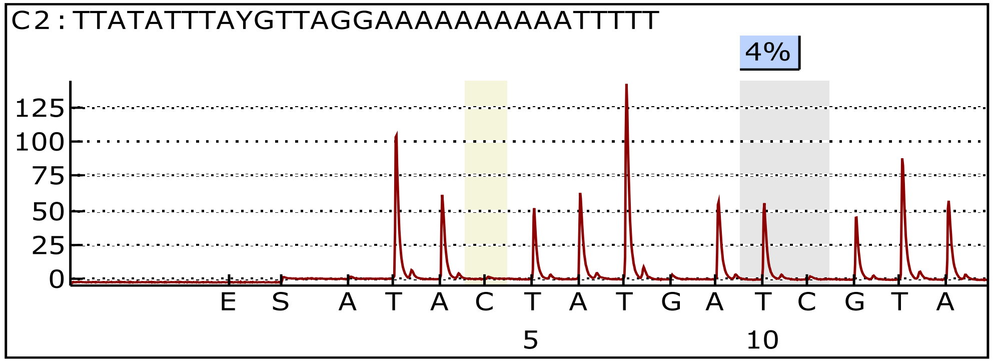


Assay Name: CZ-1
Sample ID: 37
Note:

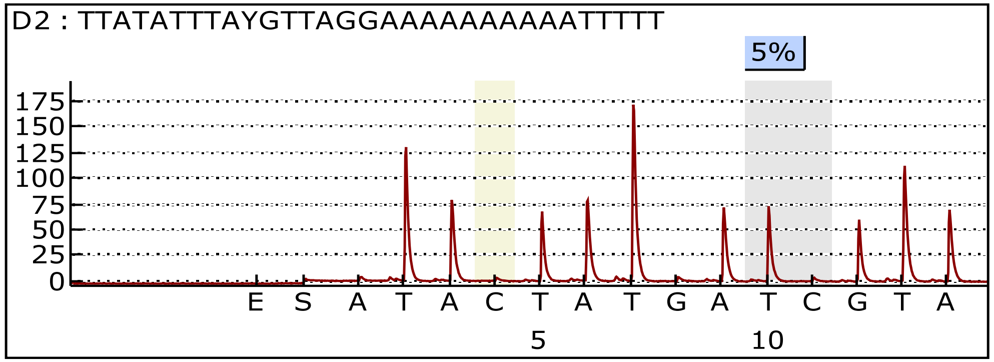


Assay Name: CZ-1
Sample ID: 38
Note:

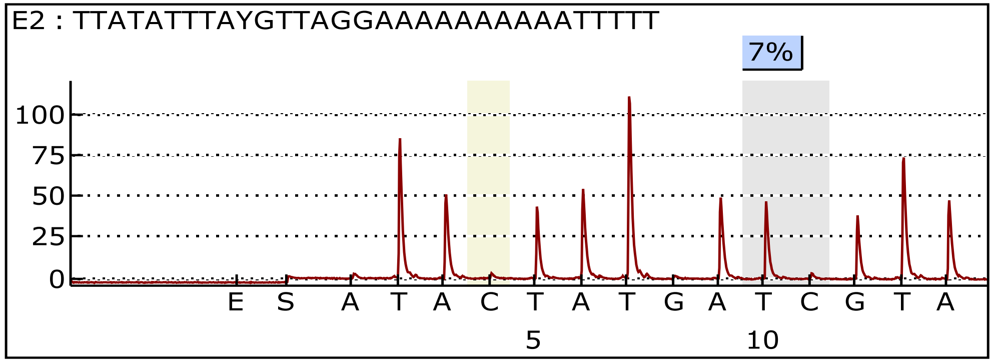


Assay Name: CZ-1
Sample ID: 39
Note:

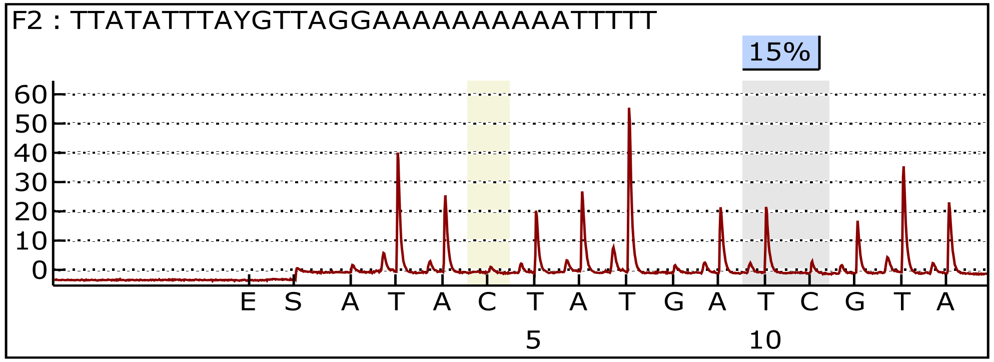

Assay Name: CZ-1
Sample ID: 40
Note:

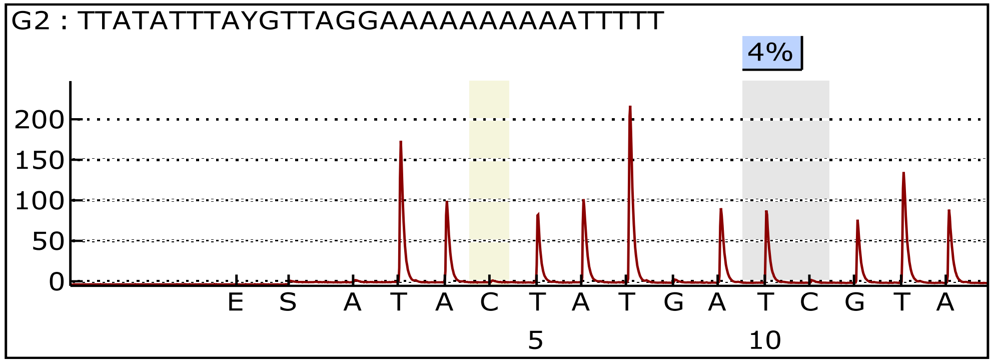


Assay Name: CZ-1
Sample ID: 41
Note:

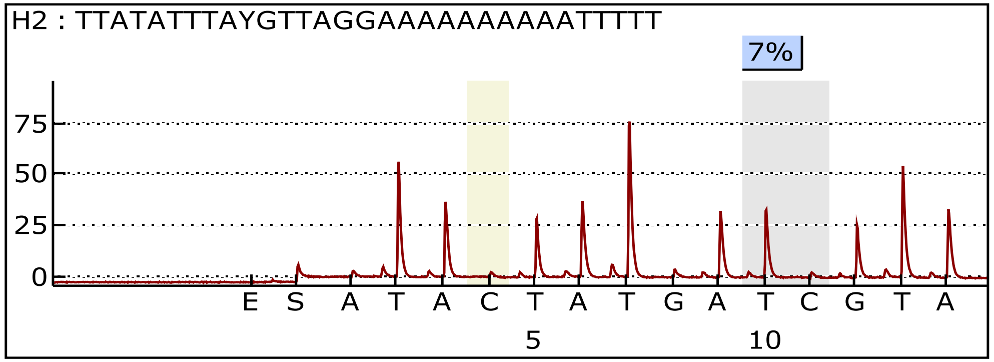


Assay Name: CZ-1
Sample ID: 42
Note:

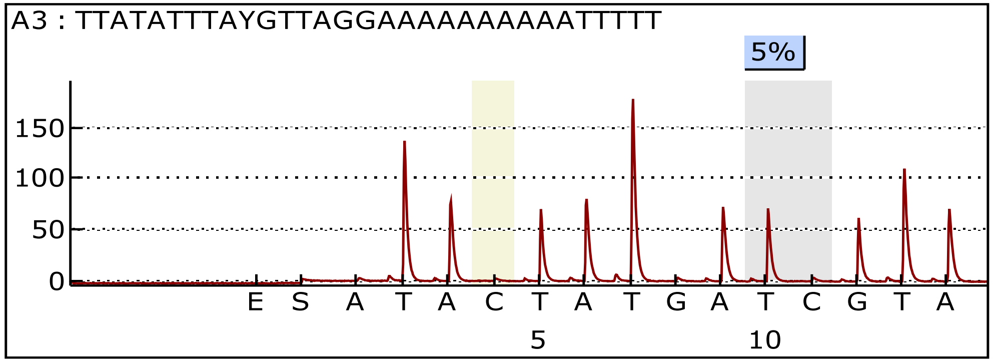


Assay Name: CZ-1
Sample ID: 43
Note:

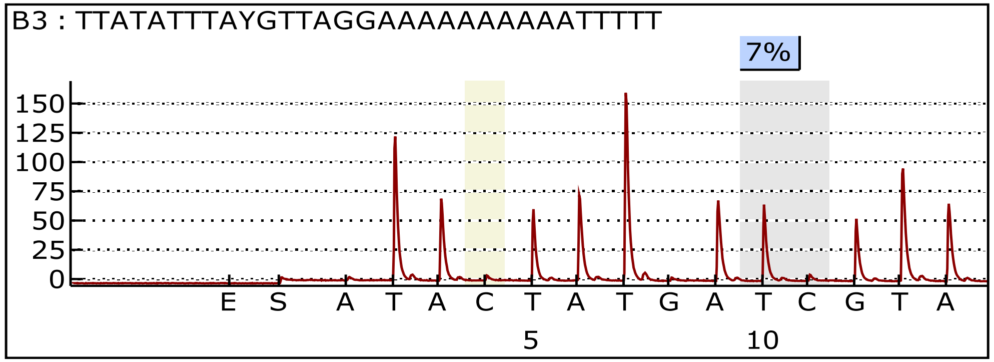


Assay Name: CZ-1
Sample ID: 44
Note:

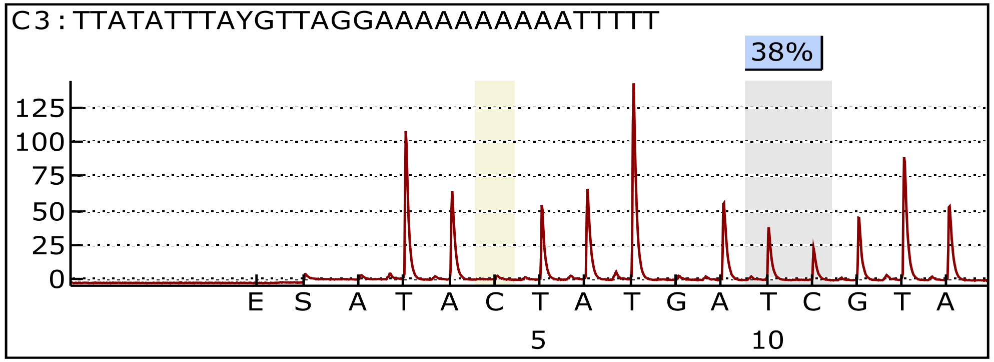


Assay Name: CZ-1
Sample ID: 45
Note:

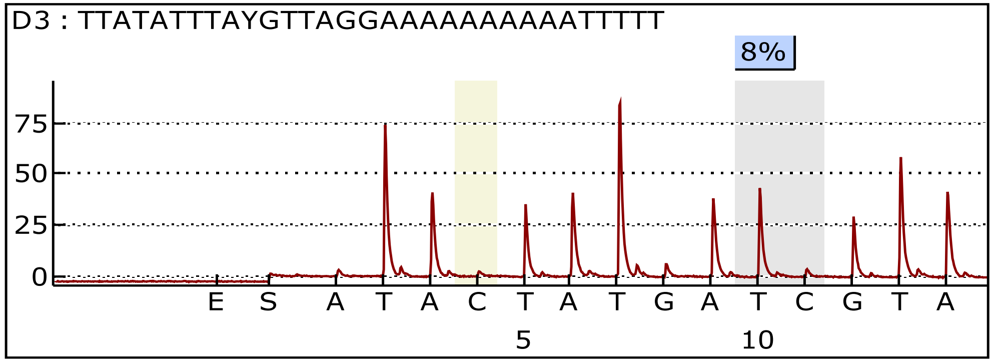

Assay Name: CZ-1
Sample ID: 46
Note:

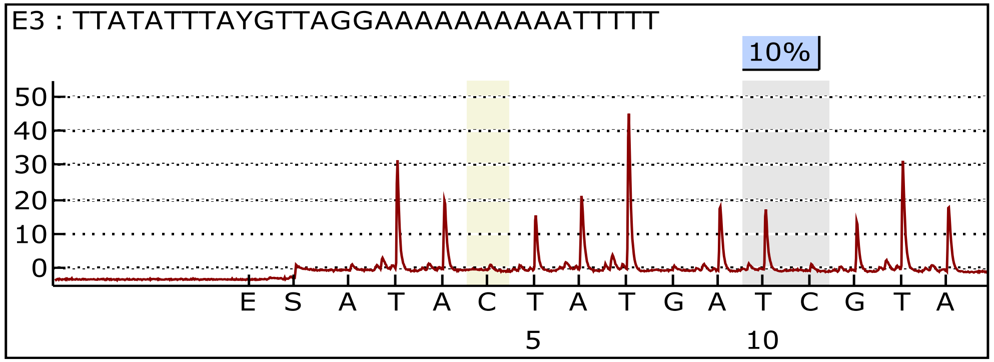


Assay Name: CZ-1
Sample ID: 47
Note:

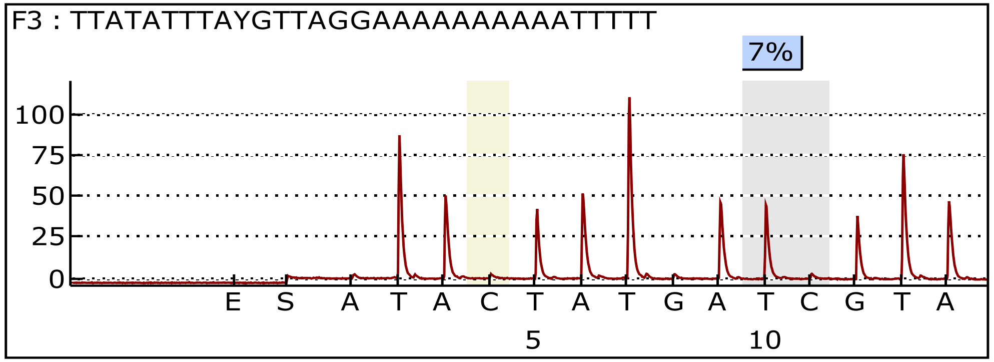


Assay Name: CZ-1
Sample ID: 48
Note:

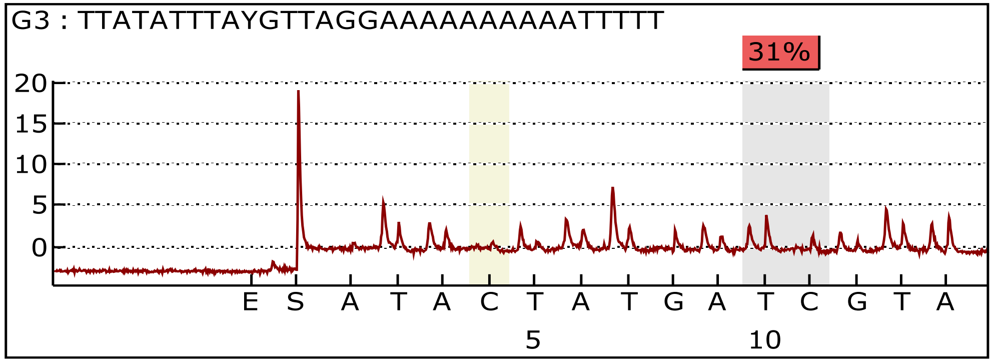


Assay Name: CZ-1
Sample ID: 49
Note:

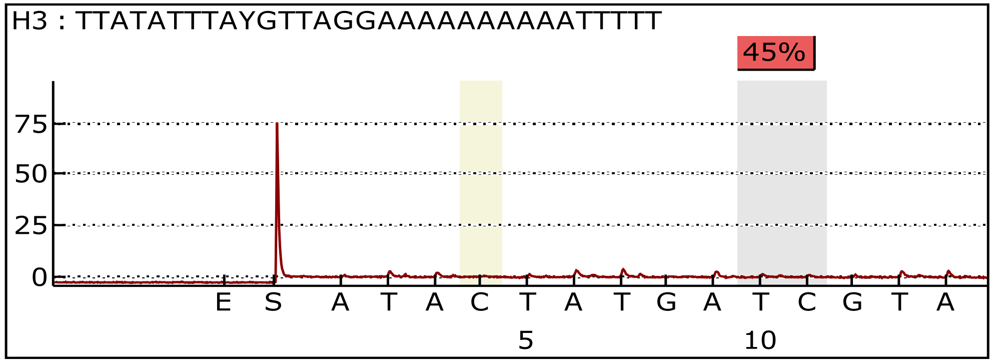


Assay Name: CZ-1
Sample ID: 50
Note:

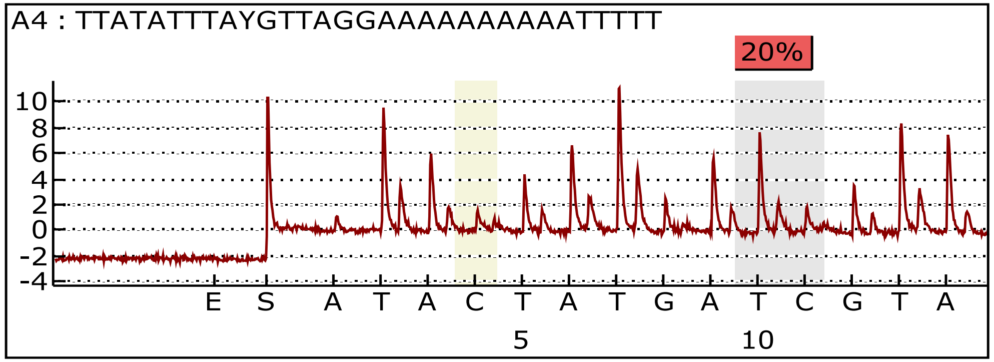


Assay Name: CZ-1
Sample ID: 51
Note:

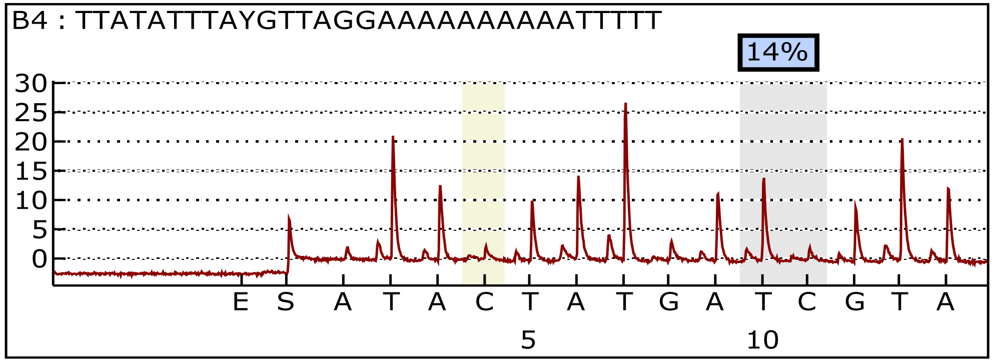

Assay Name: CZ-1
Sample ID: 52
Note:

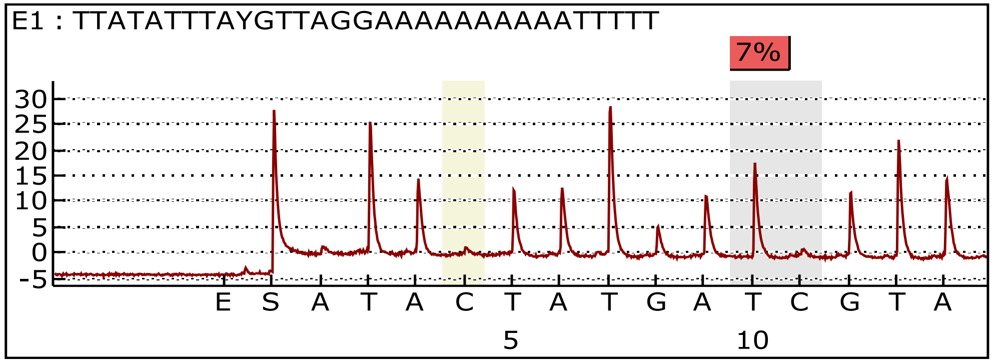


Assay Name: CZ-1
Sample ID: 53
Note:

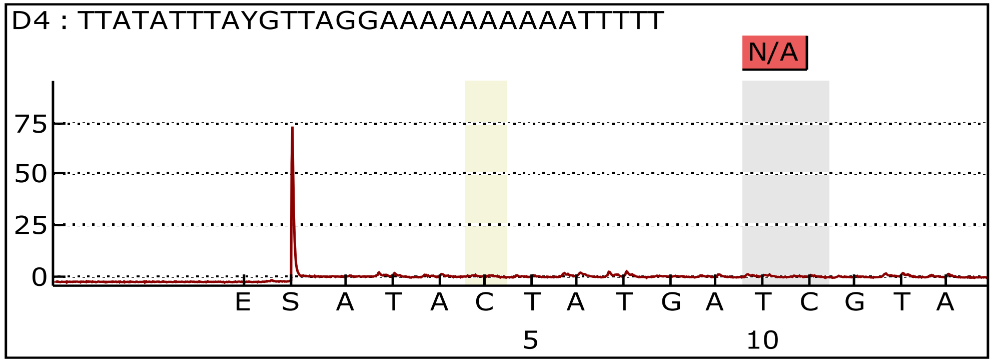


Assay Name: CZ-1
Sample ID: 54
Note:

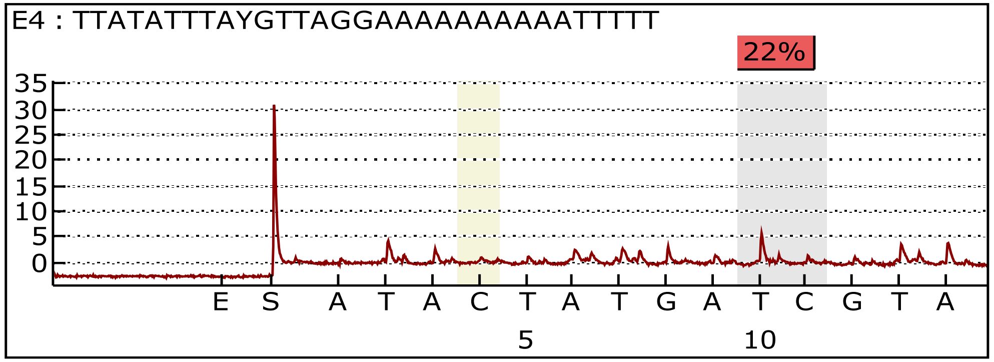


Assay Name: CZ-1
Sample ID: 55
Note:

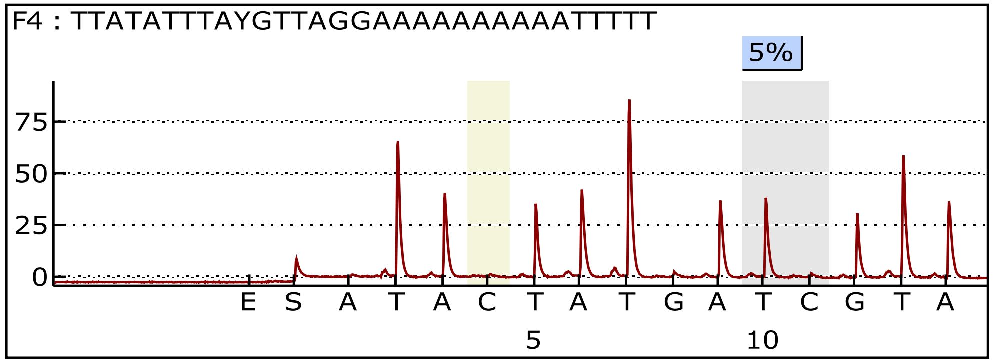

Assay Name: CZ-1
Sample ID: 103
Note:

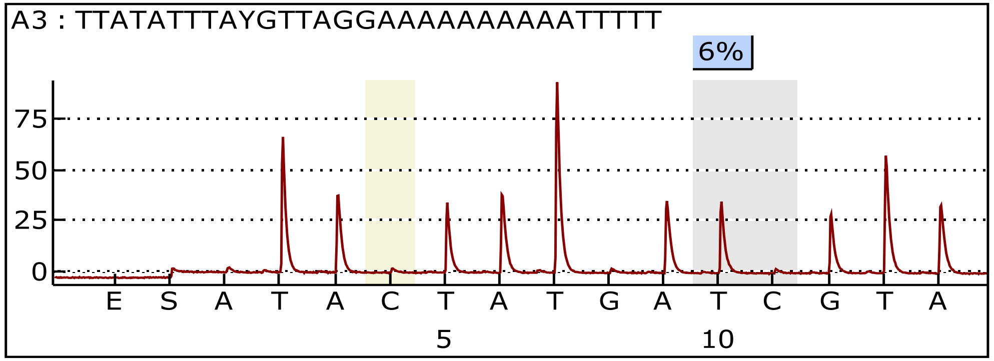


Assay Name: CZ-1
Sample ID: 111
Note:

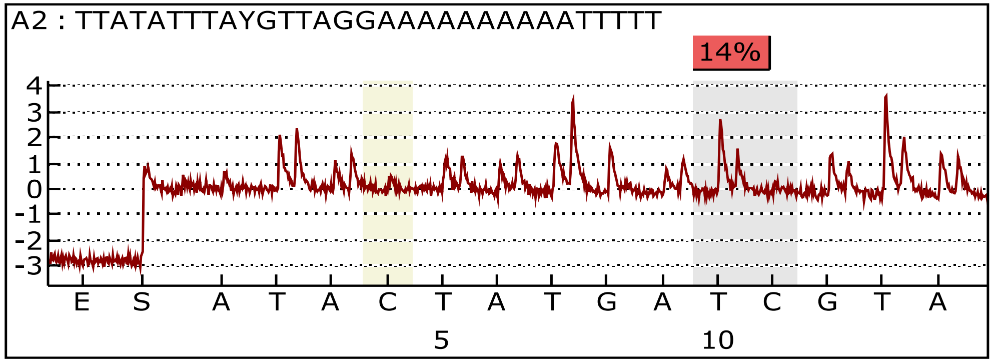


Assay Name: CZ-1
Sample ID: 112
Note:

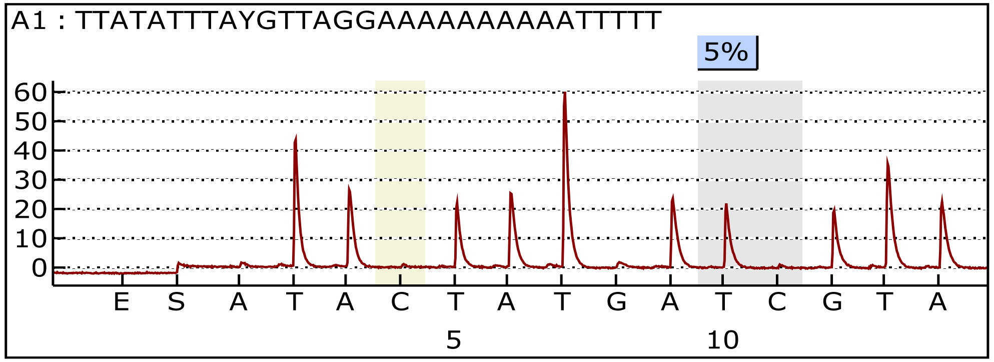


Assay Name: CZ-1
Sample ID: 113
Note:

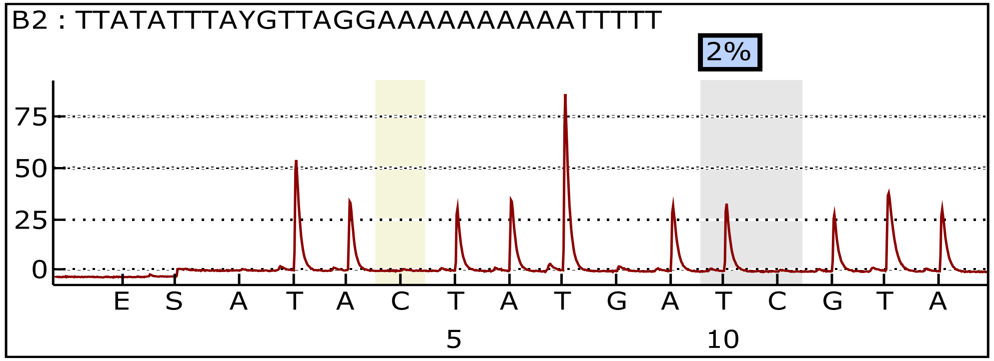


Assay Name: CZ-1
Sample ID: 114
Note:

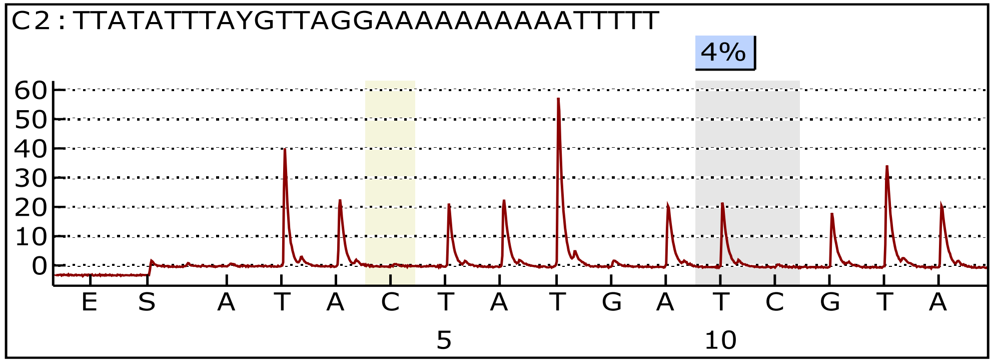


Assay Name: CZ-1
Sample ID: 136
Note:

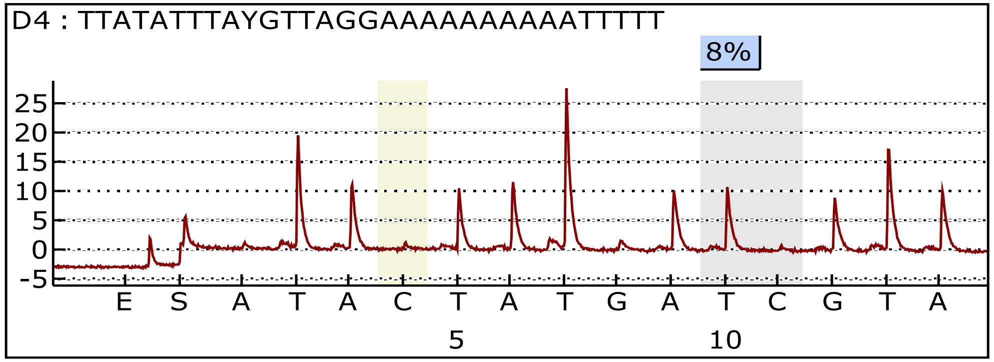

\

Assay Name: CZ-1
Sample ID: 150
Note:

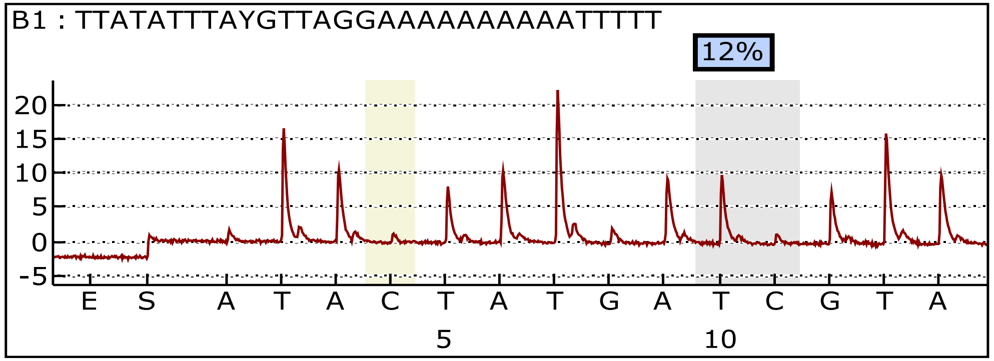


Assay Name: CZ-1
Sample ID: 154
Note:

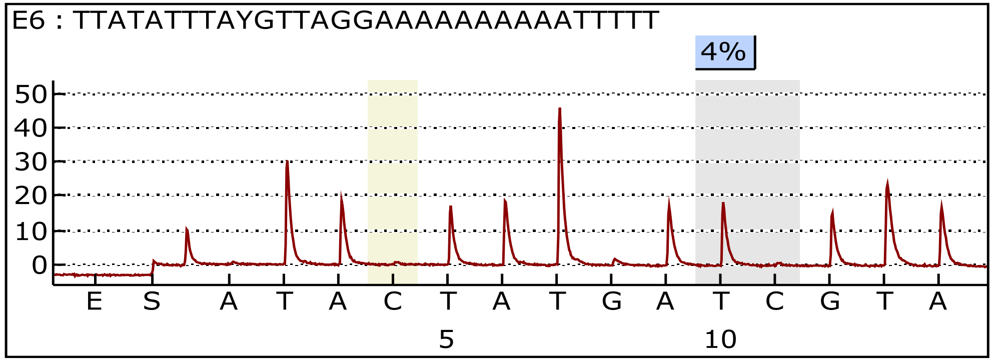


Assay Name: CZ-1
Sample ID: 151
Note:

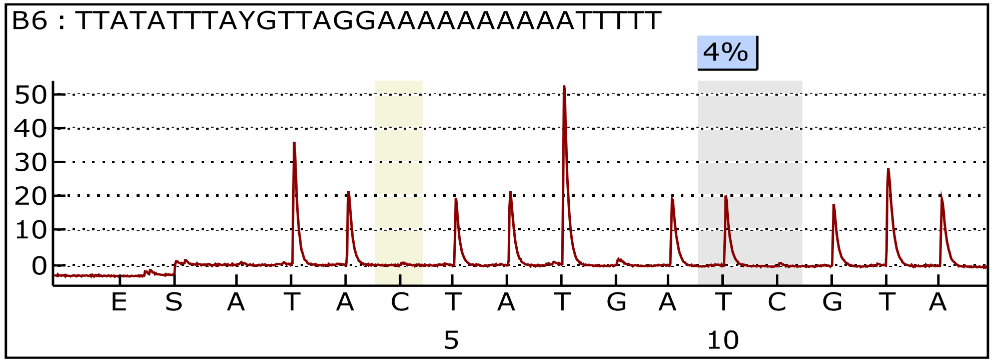


Assay Name: CZ-1
Sample ID: 161
Note:

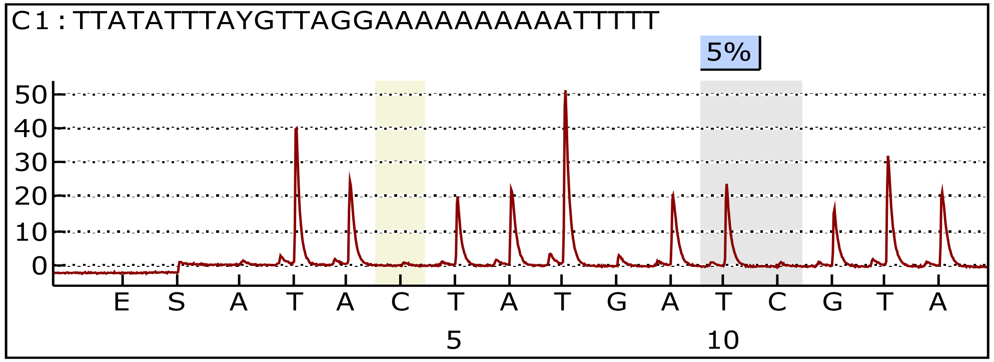


Assay Name: CZ-1
Sample ID: 152
Note:

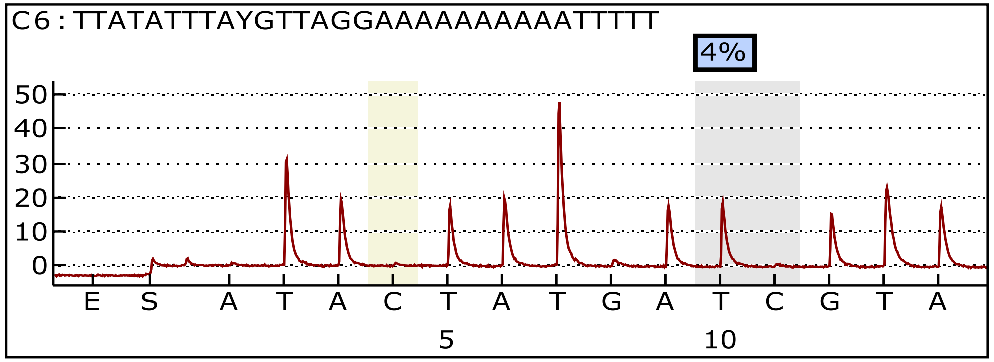


Assay Name: CZ-1
Sample ID: 165
Note:

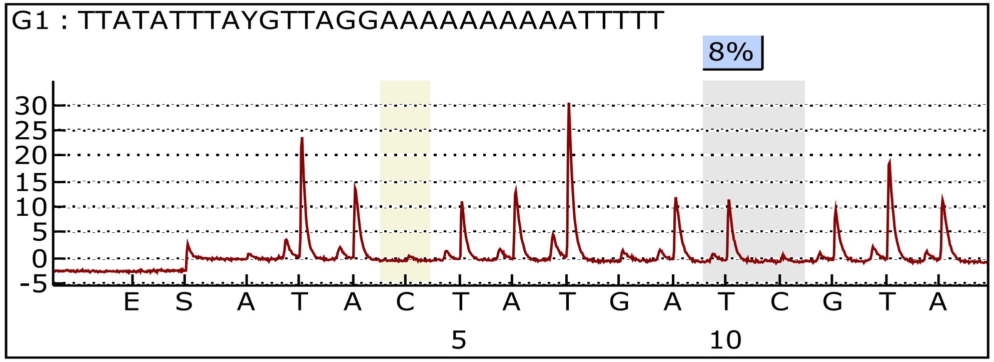

Assay Name: CZ-1
Sample ID: 166
Note:

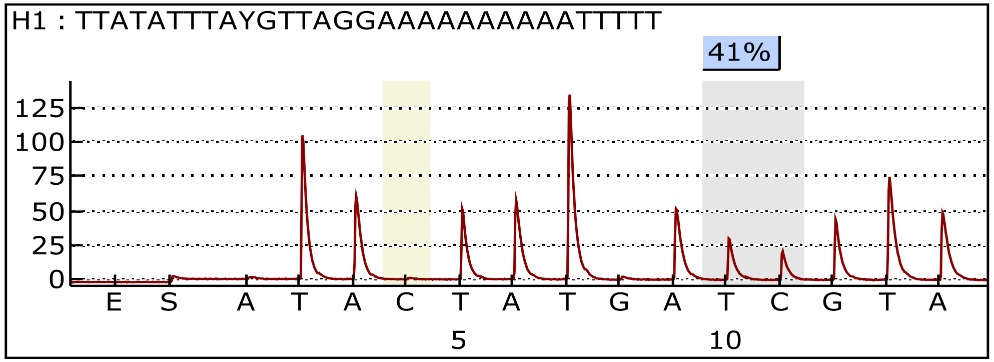


Assay Name: CZ-1
Sample ID: 182
Note:

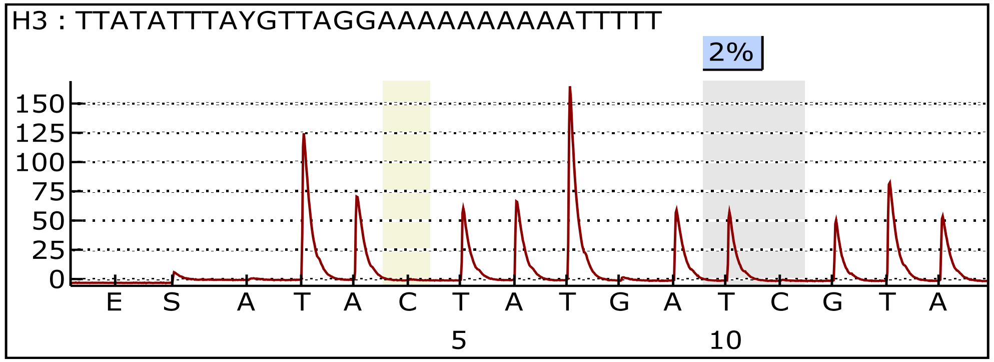


Assay Name: CZ-1
Sample ID: 167
Note:

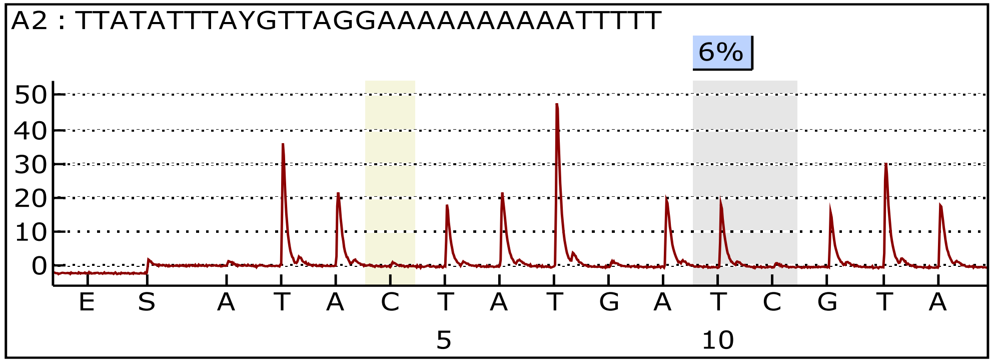


Assay Name: CZ-1
Sample ID: 183
Note:

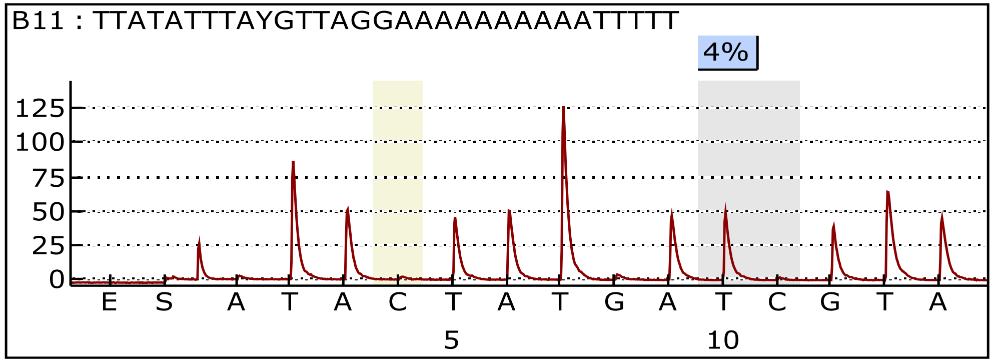


Assay Name: CZ-1
Sample ID: 178
Note:

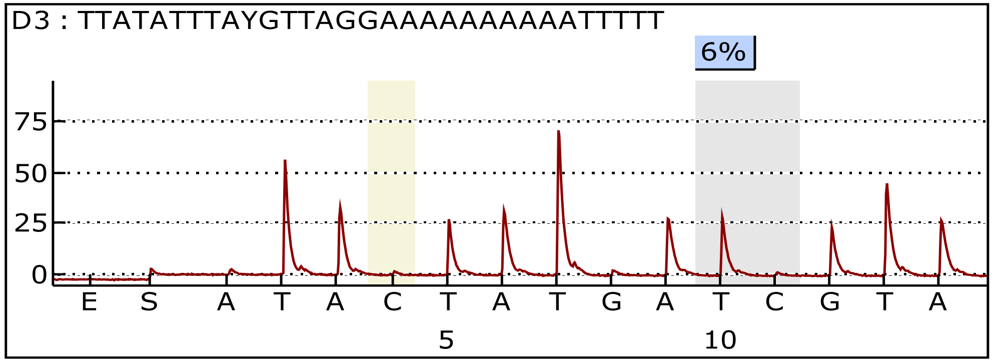


Assay Name: CZ-1
Sample ID: 185
Note:

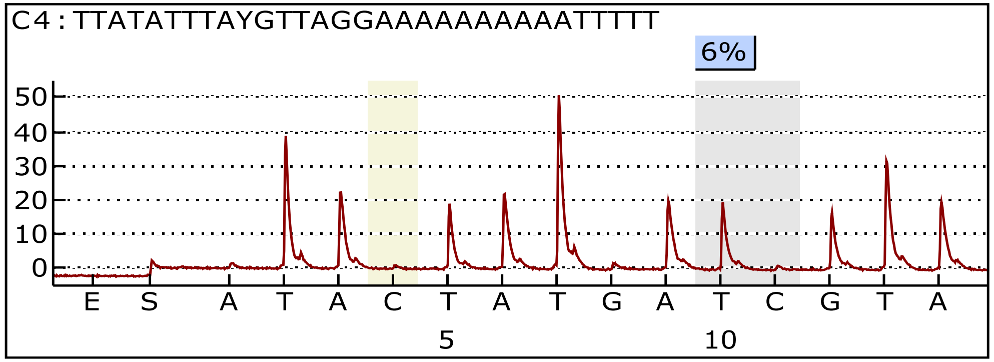

Assay Name: CZ-1
Sample ID: 189
Note:

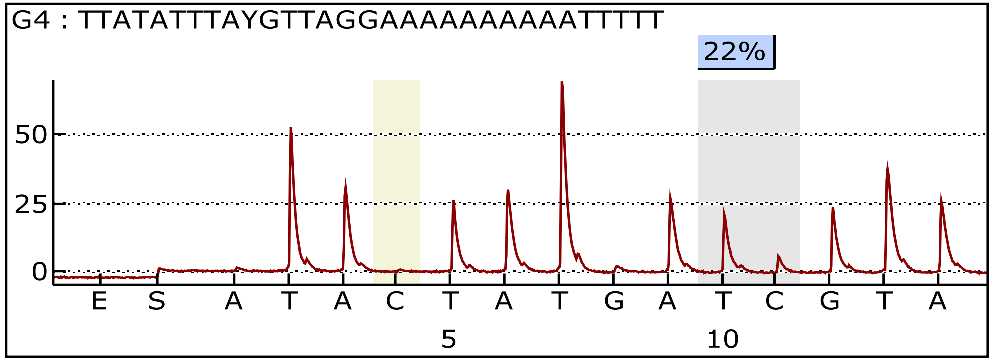


**Assay Name: CZ-1**
**Sample ID: 195**
**Note:**

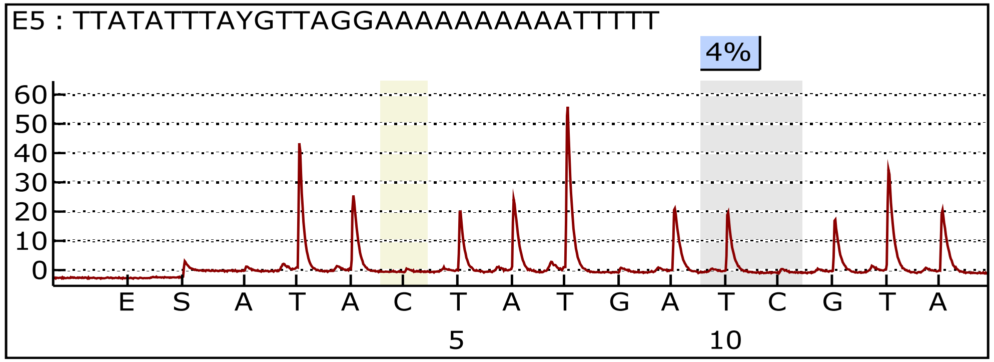


Assay Name: CZ-1
Sample ID: 191
Note:

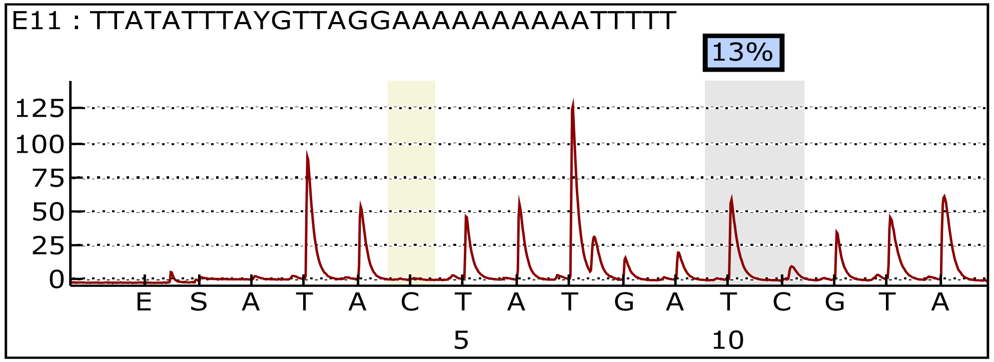


Assay Name: CZ-1
Sample ID: 204
Note:

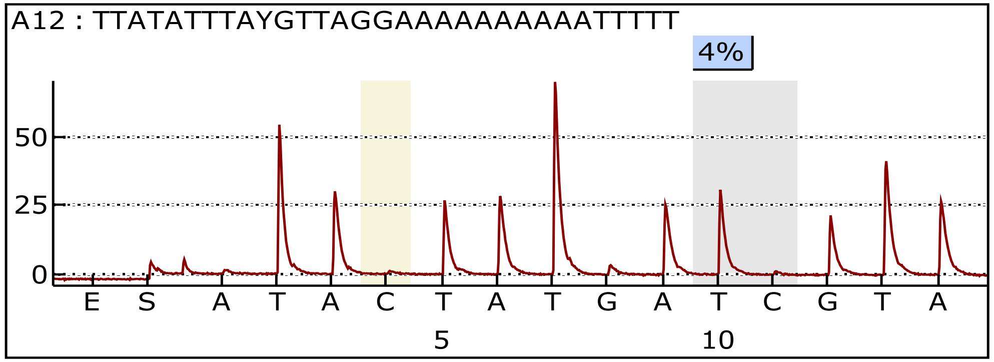


**Assay Name: CZ-1**
**Sample ID: 194**
**Note:**

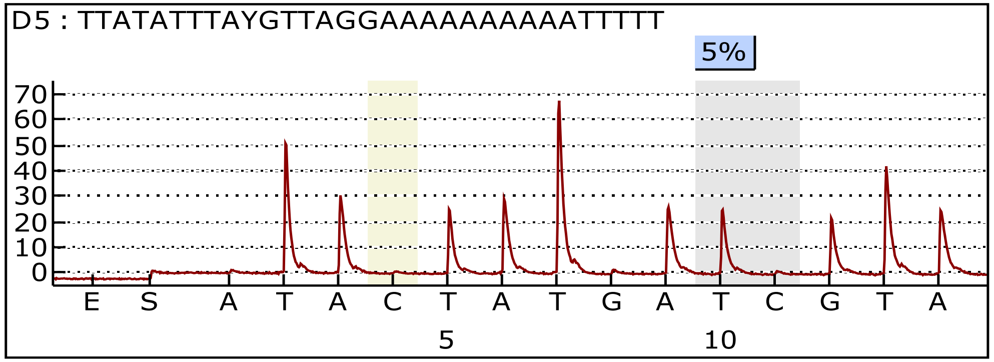


Assay Name: CZ-1
Sample ID: 208
Note:

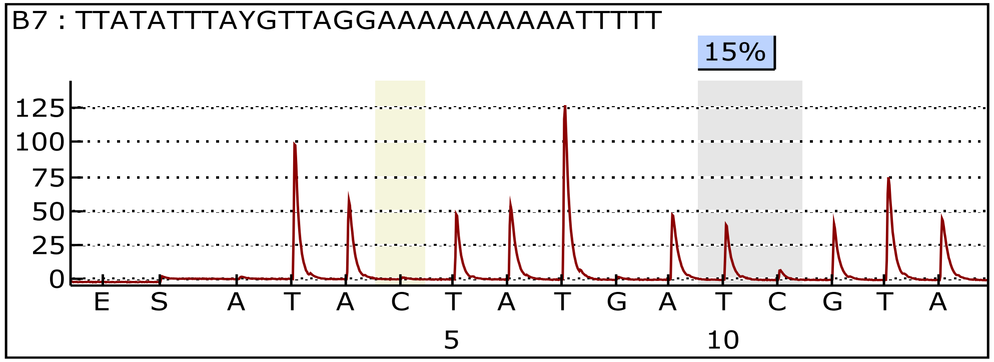

Assay Name: CZ-1
Sample ID: 101
Note:

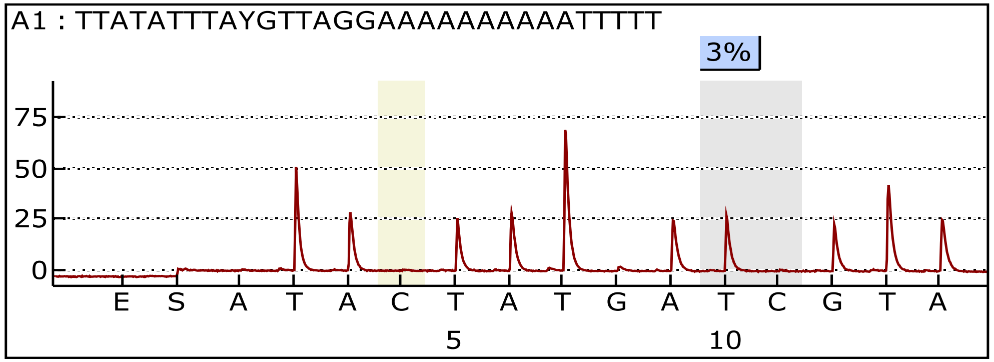


Assay Name: CZ-1
Sample ID: 102
Note:

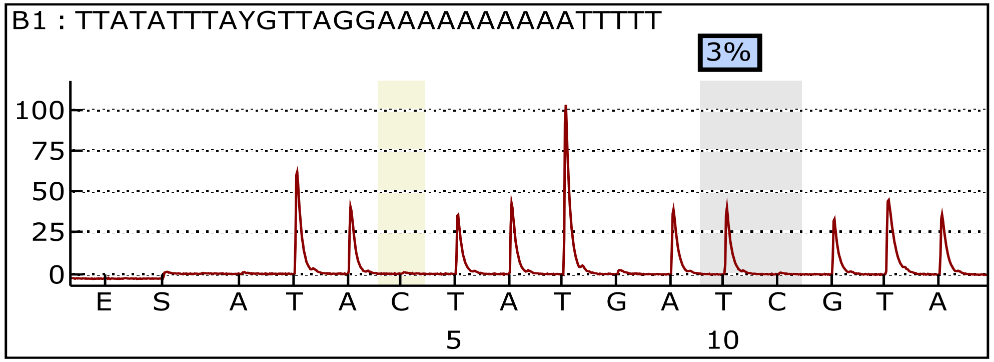


Assay Name: CZ-1
Sample ID: 116
Note:

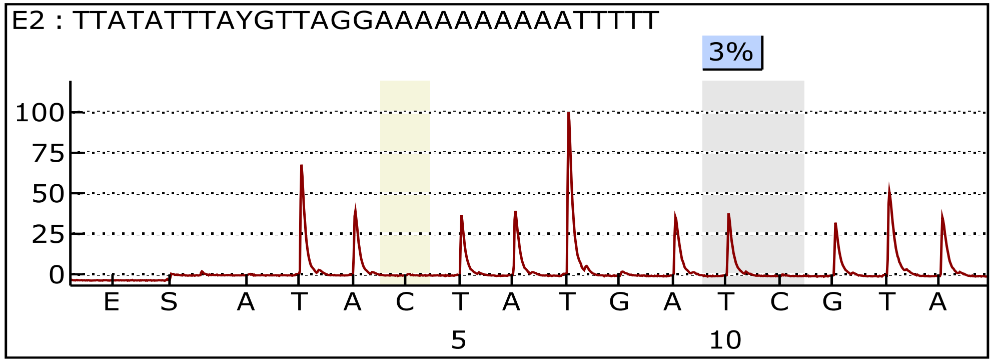


Assay Name: CZ-1
Sample ID: 118
Note:

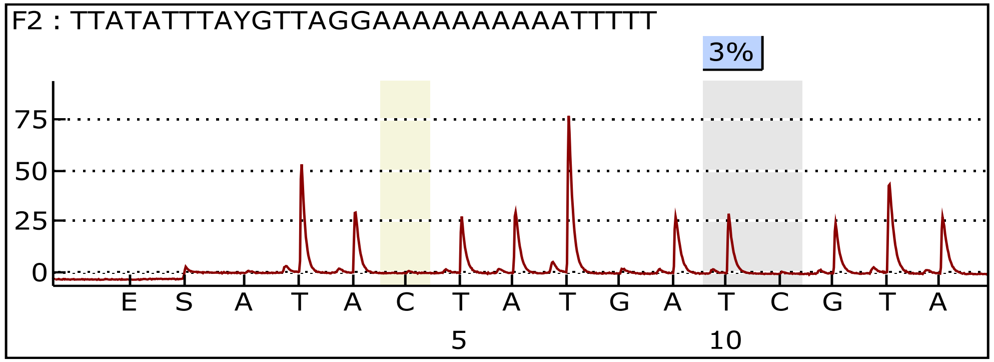


Assay Name: CZ-1
Sample ID: 120
Note:

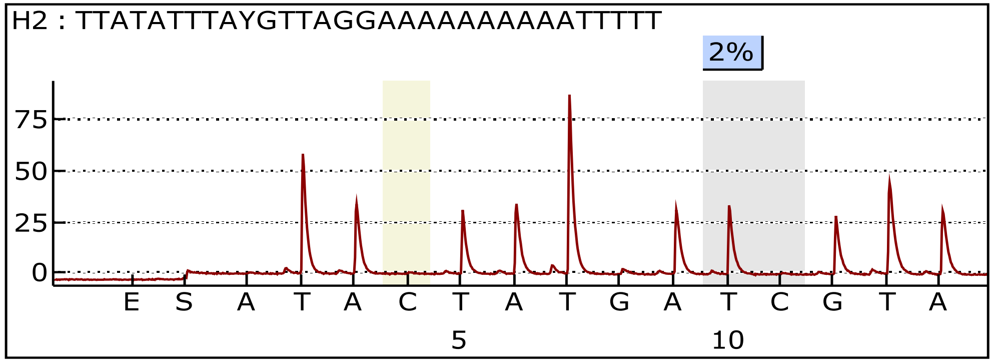


Assay Name: CZ-1
Sample ID: 127
Note:

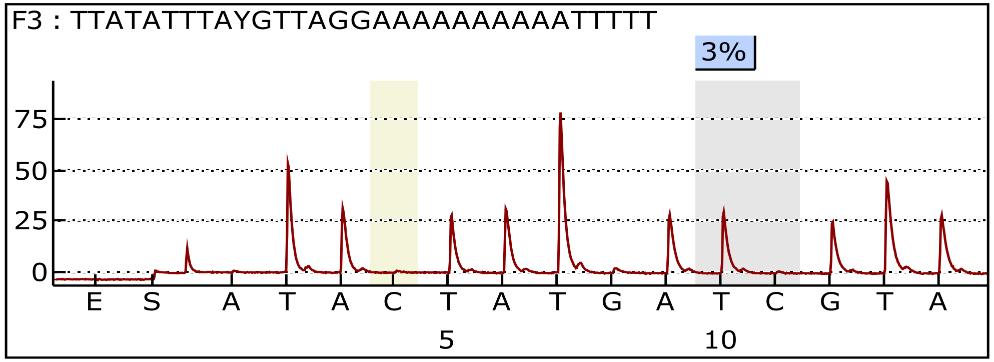

Assay Name: CZ-1
Sample ID: 128
Note:

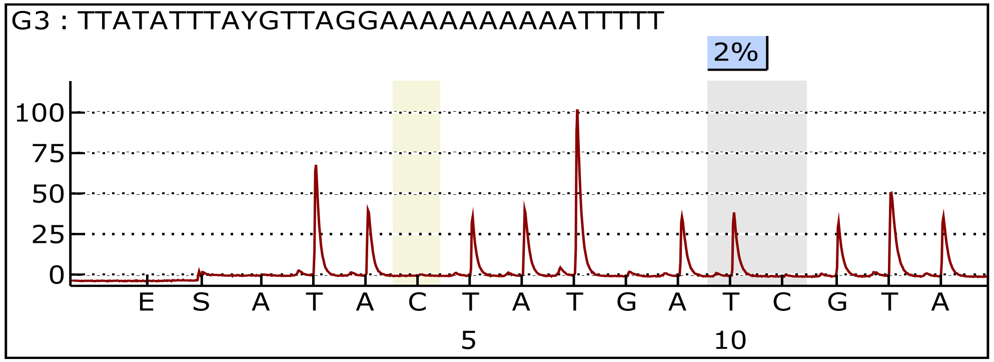


Assay Name: CZ-1
Sample ID: 129
Note:

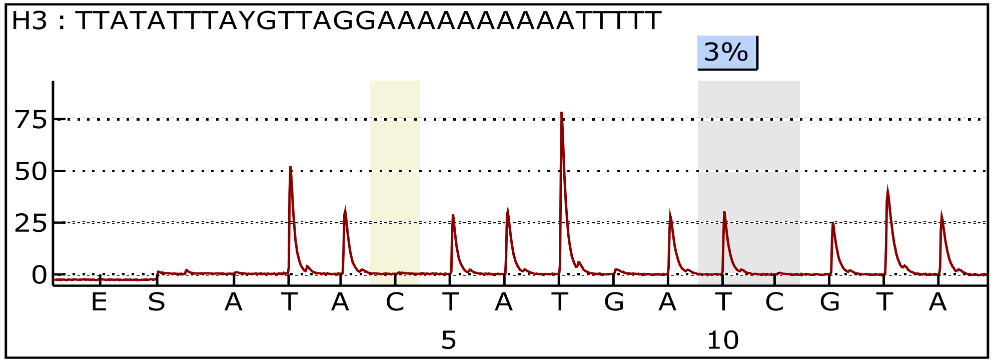


Assay Name: CZ-1
Sample ID: 130
Note:

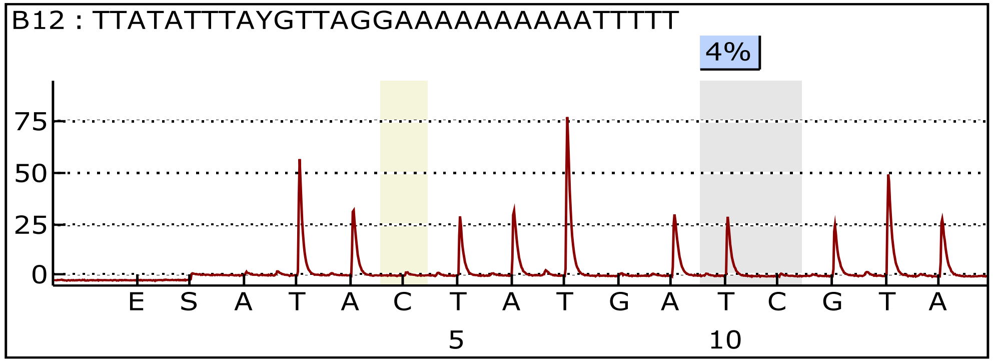


Assay Name: CZ-1
Sample ID: 135
Note:

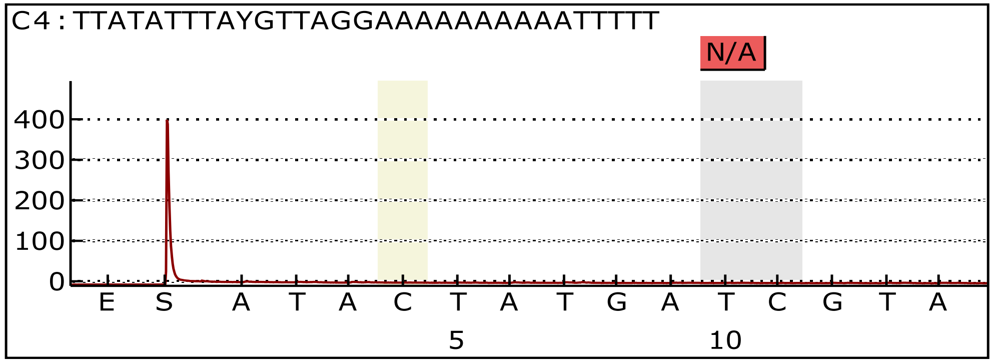


Assay Name: CZ-1
Sample ID: 139
Note:

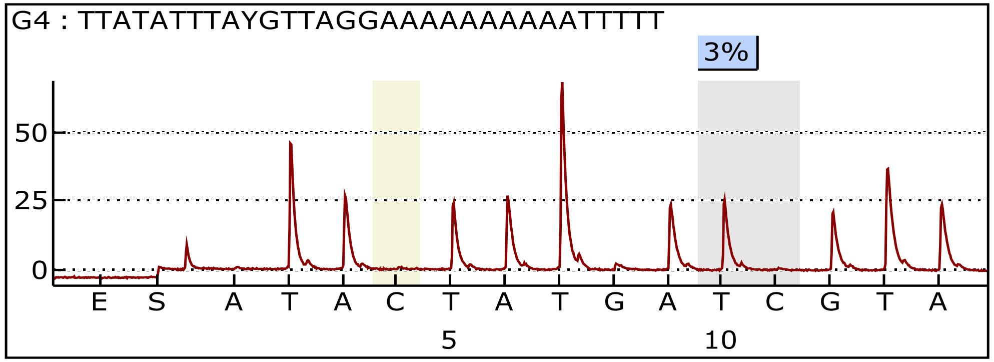


Assay Name: CZ-1
Sample ID: 147
Note:

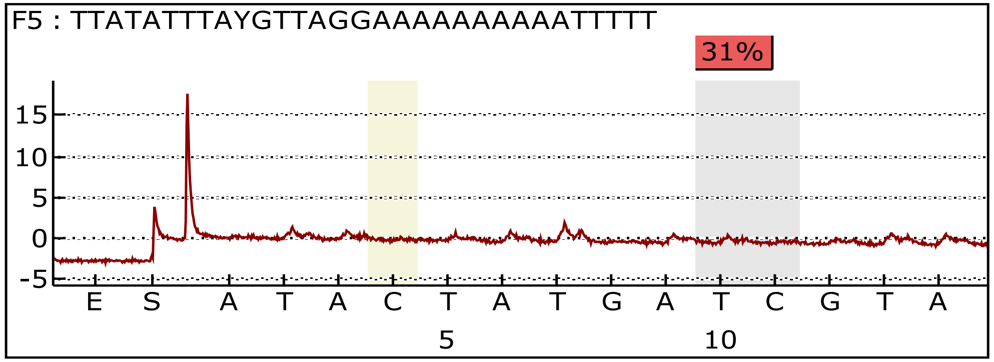

Assay Name: CZ-1
Sample ID: 148
Note:

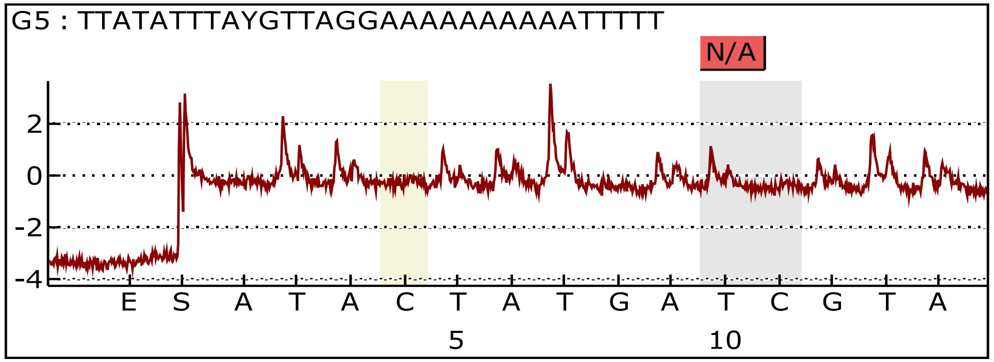


Assay Name: CZ-1
Sample ID: 158
Note:

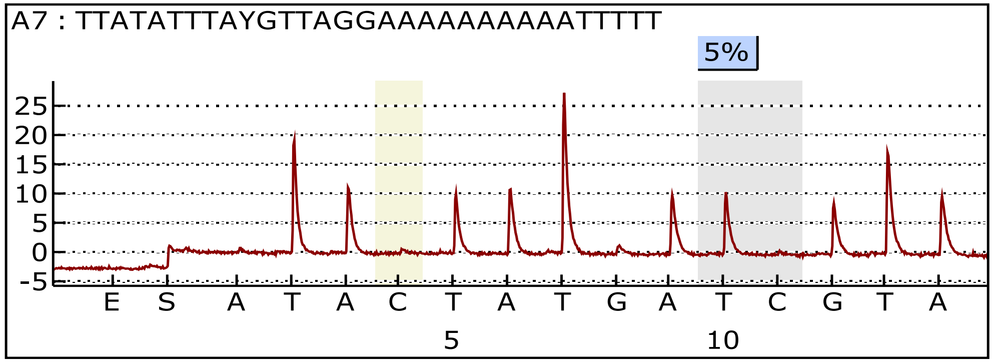


Assay Name: CZ-1
Sample ID: 155
Note:

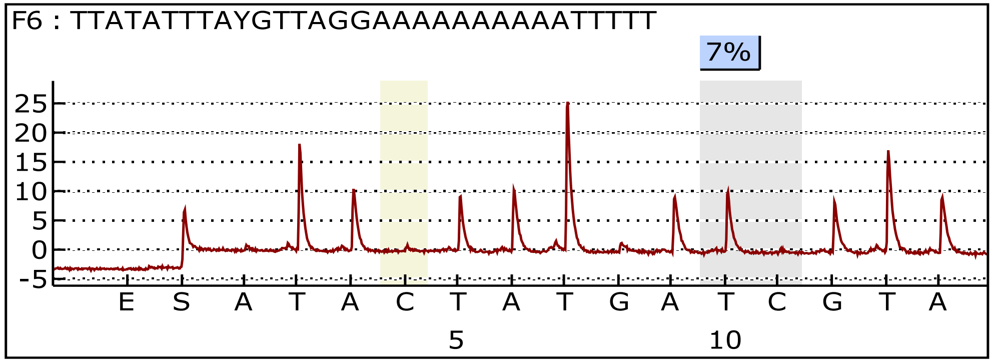


Assay Name: CZ-1
Sample ID: 159
Note:

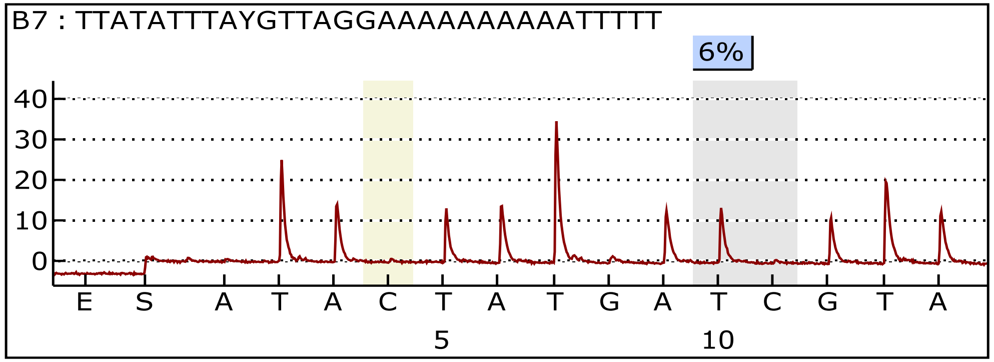


Assay Name: CZ-1
Sample ID: 156
Note:

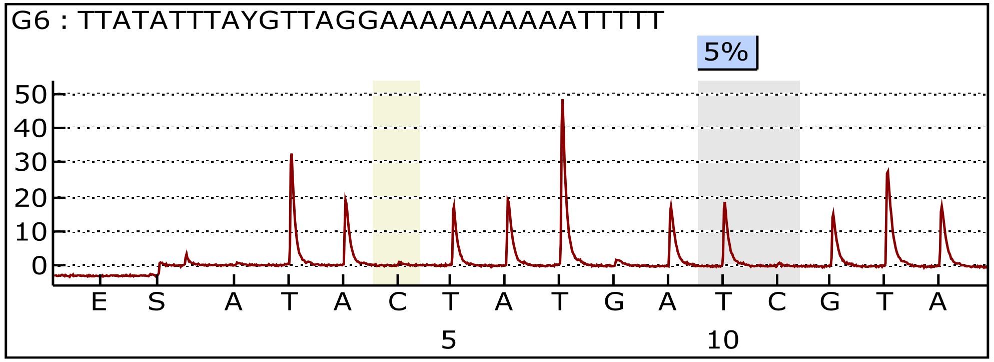


Assay Name: CZ-1
Sample ID: 160
Note:

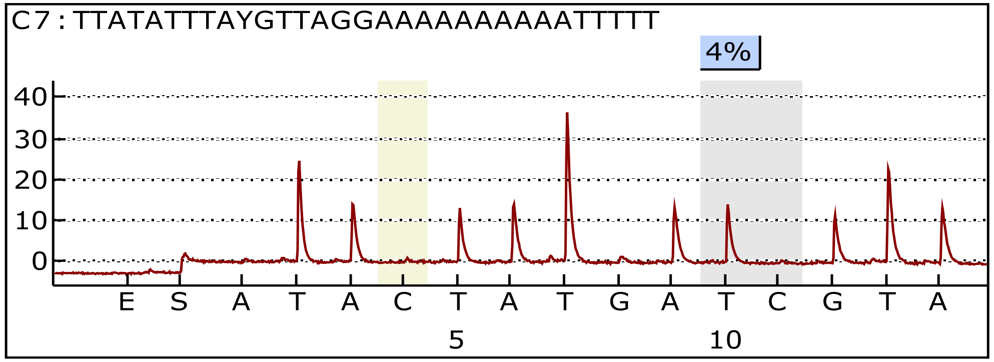

Assay Name: CZ-1
Sample ID: 162
Note:

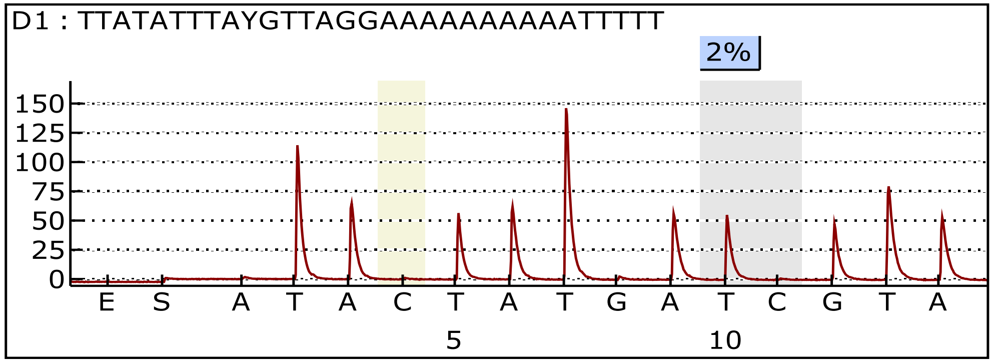


Assay Name: CZ-1
Sample ID: 164
Note:

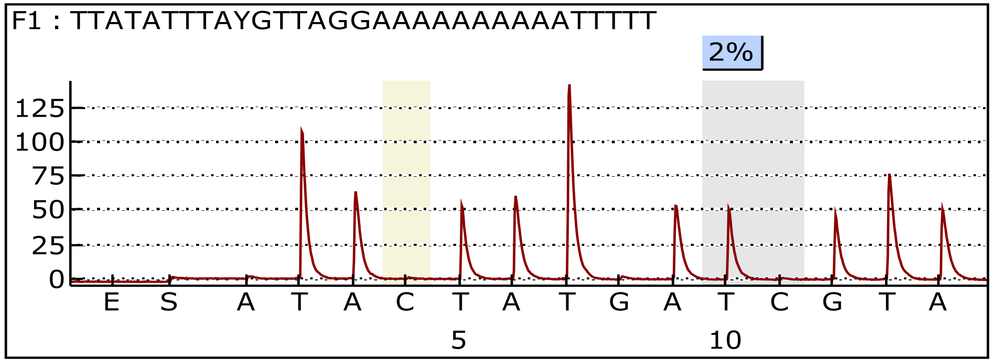


Assay Name: CZ-1
Sample ID: 168
Note:

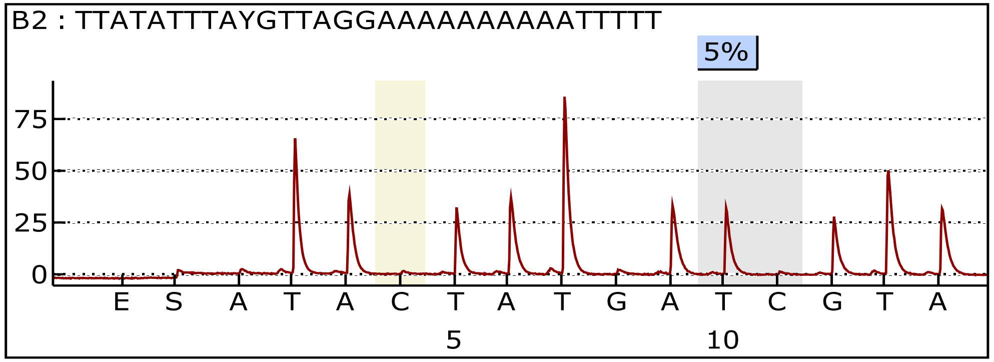


Assay Name: CZ-1
Sample ID: 169
Note:

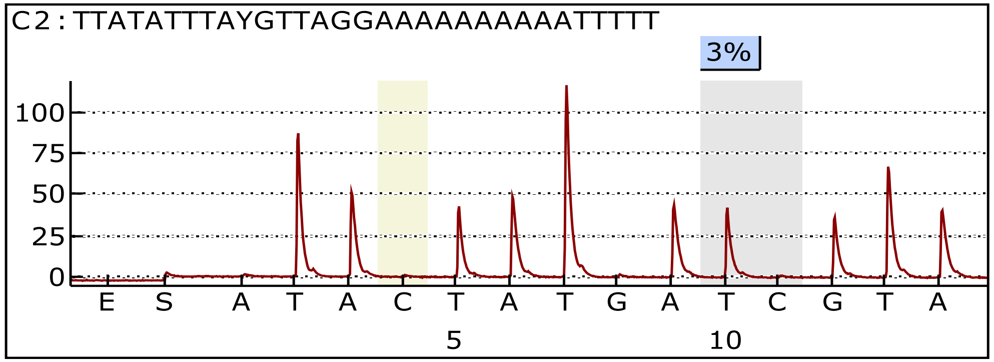


Assay Name: CZ-1
Sample ID: 172
Note:

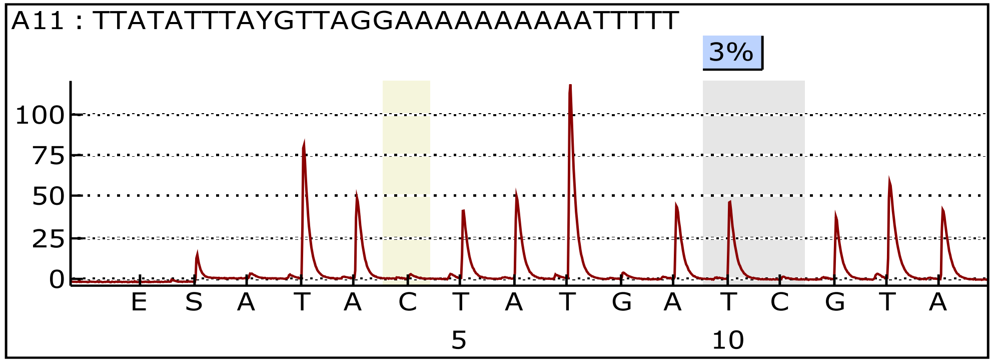


Assay Name: CZ-1
Sample ID: 173
Note:

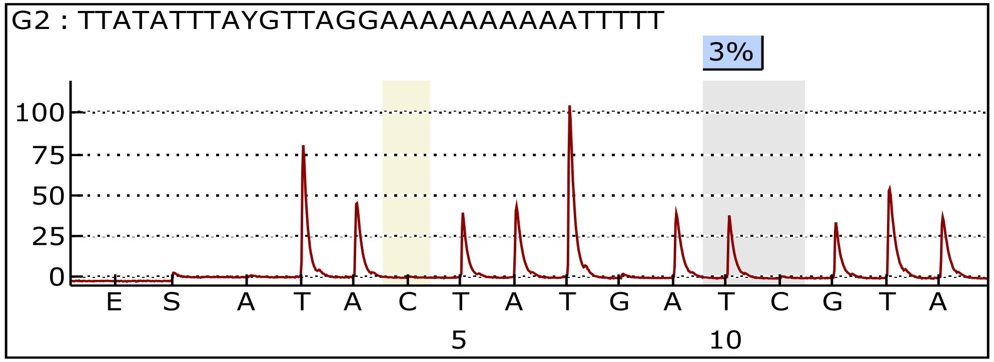

Assay Name: CZ-1
Sample ID: 177
Note:

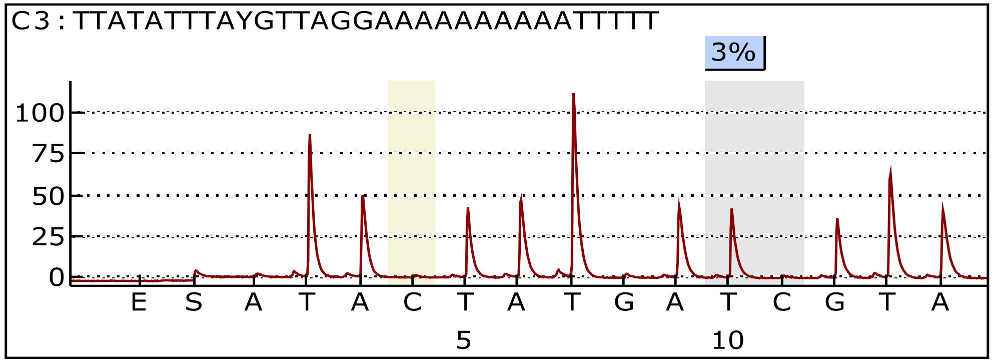


Assay Name: CZ-1
Sample ID: 186
Note:

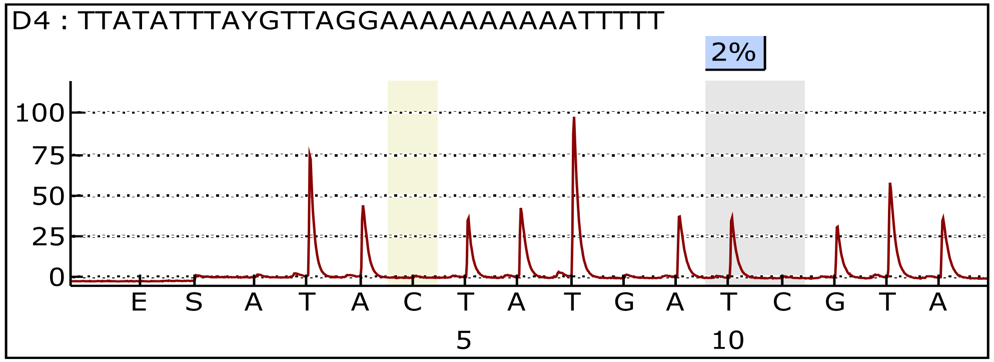


Assay Name: CZ-1
Sample ID: 181
Note:

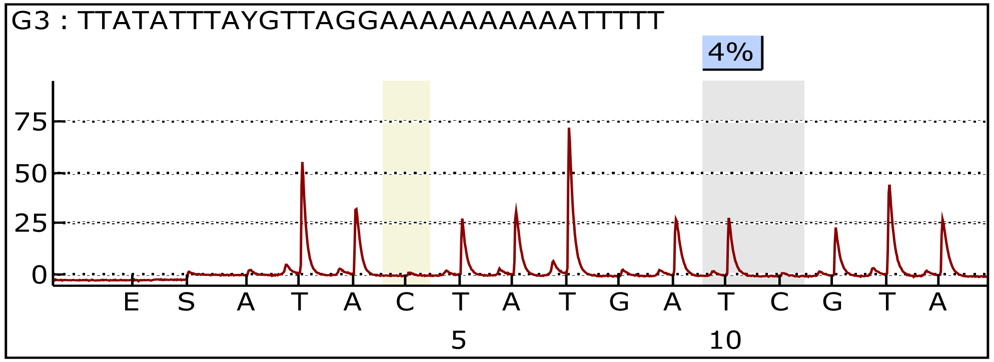


Assay Name: CZ-1
Sample ID: 190
Note:

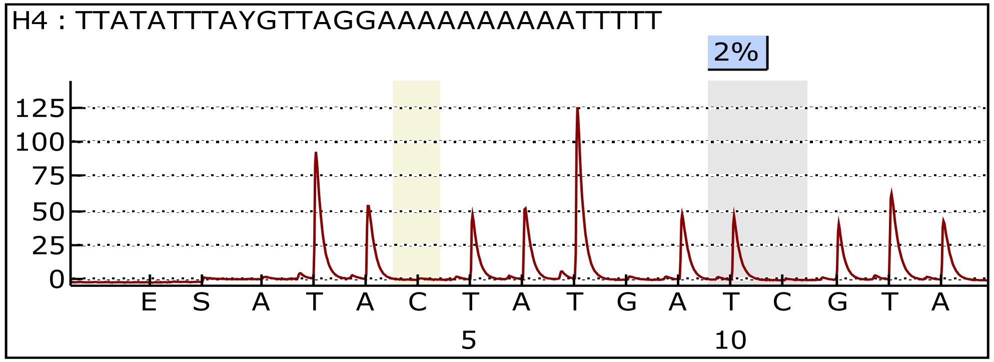


Assay Name: CZ-1
Sample ID: 184
Note:

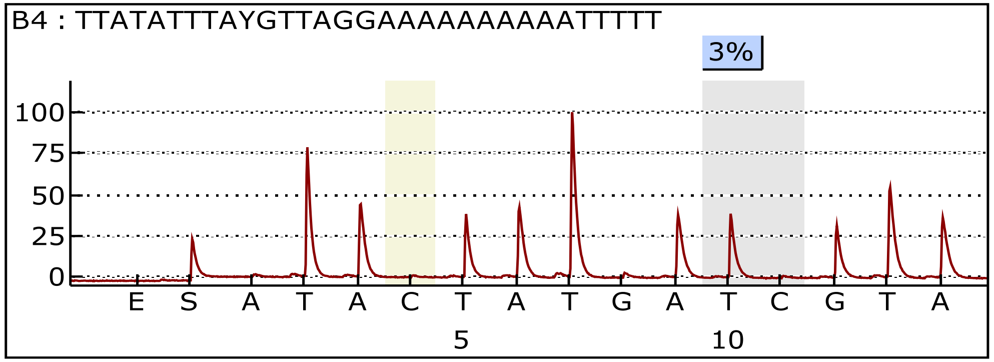


Assay Name: CZ-1
Sample ID: 192
Note:

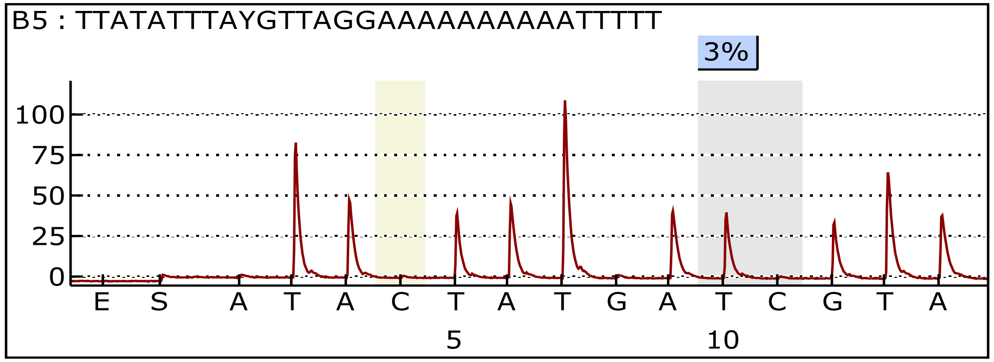

**Assay Name: CZ-1**
**Sample ID: 196**
**Note:**

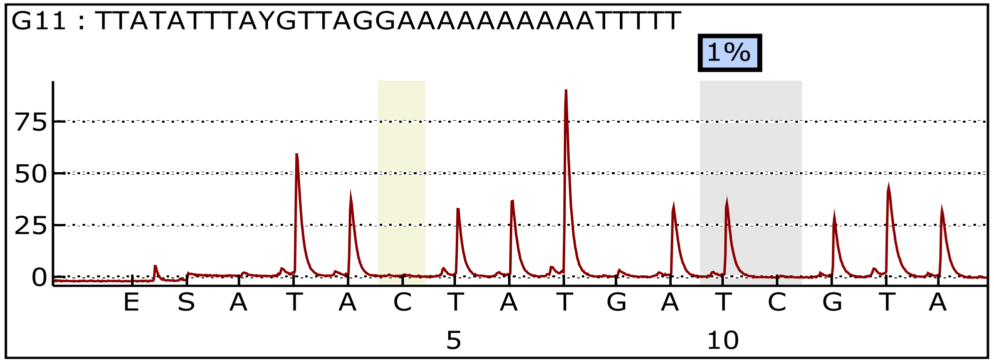


Assay Name: CZ-1
Sample ID: 199
Note:

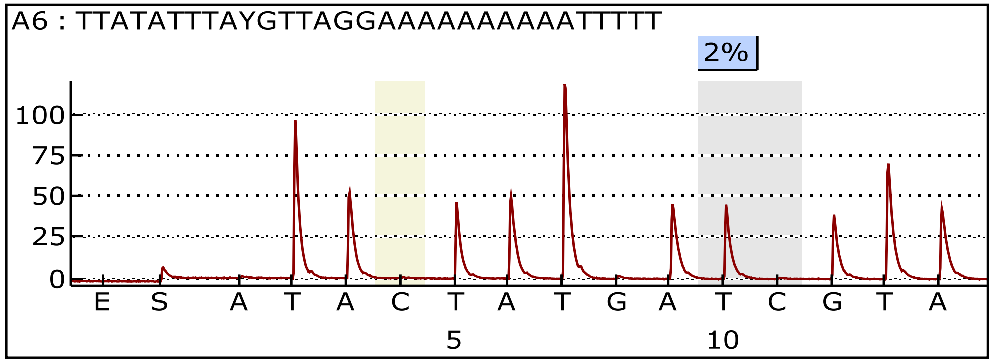


Assay Name: CZ-1
Sample ID: 197
Note:

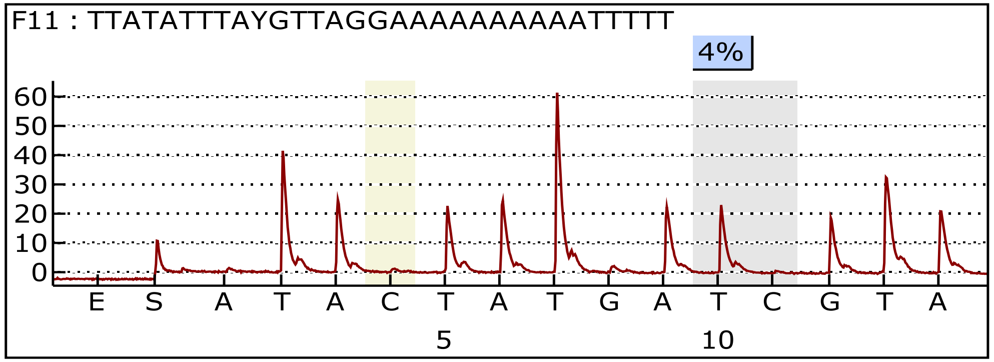


Assay Name: CZ-1
Sample ID: 201
Note:

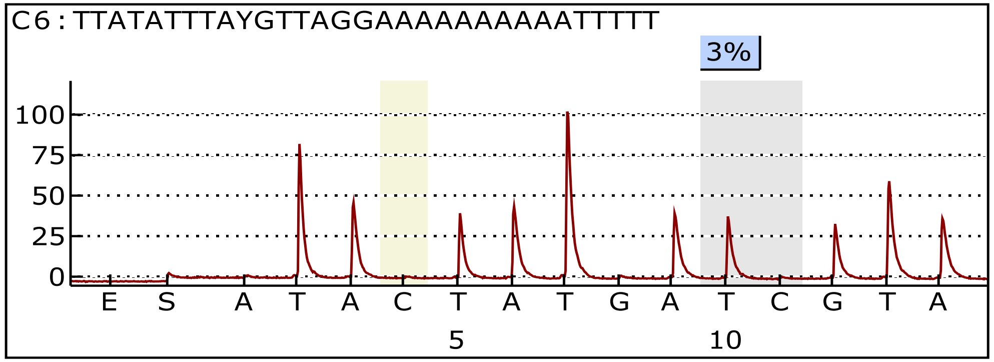


Assay Name: CZ-1
Sample ID: 198
Note:

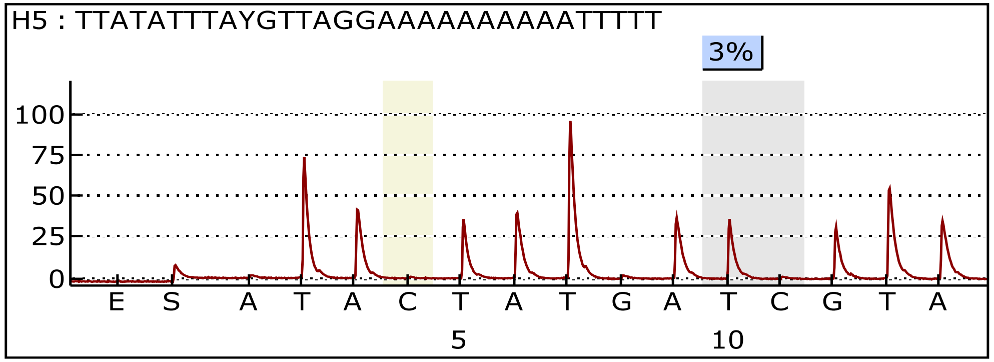

# Cg27539833

Assay Name: CZ-3
Sample ID: 5
Note:

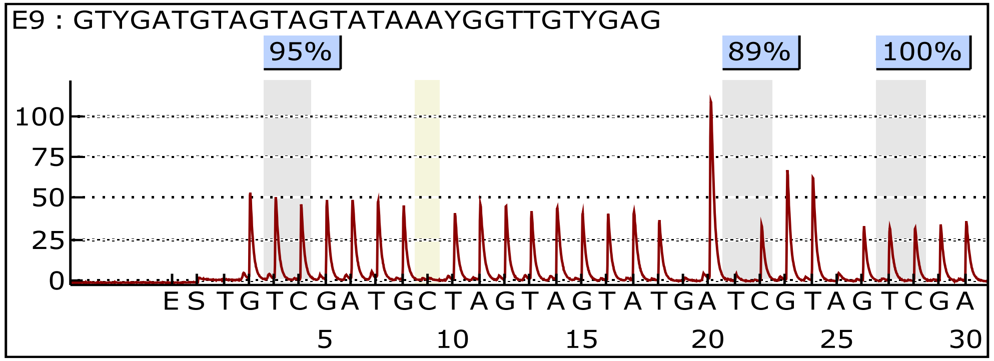


Assay Name: CZ-3
Sample ID: 8
Note:

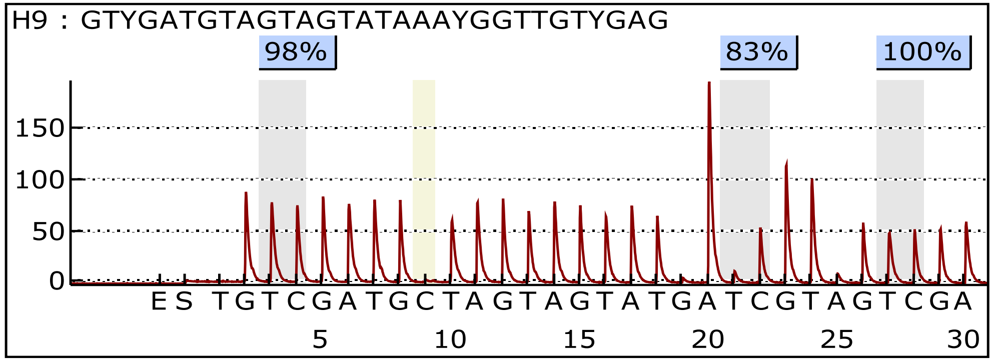


Assay Name: CZ-3
Sample ID: 6
Note:

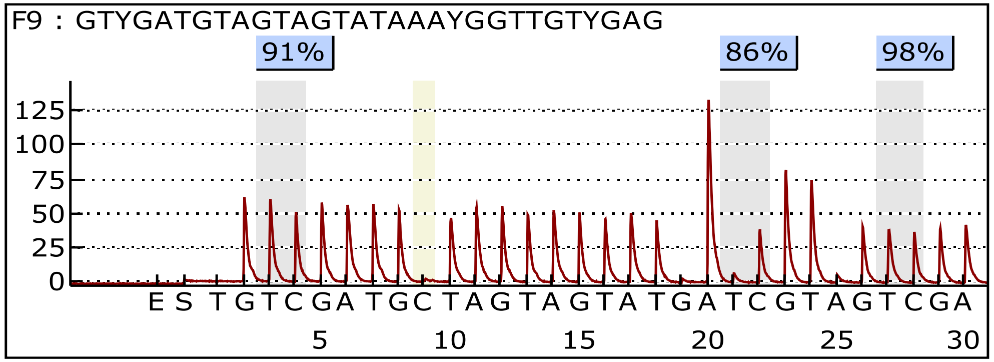


Assay Name: CZ-3
Sample ID: 9
Note:

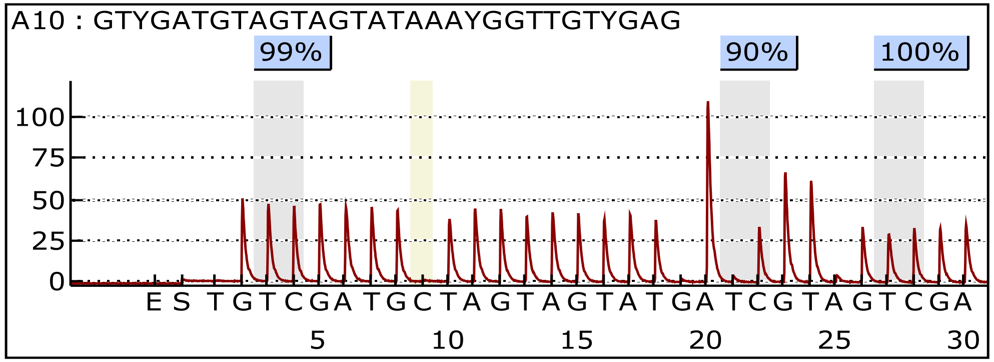


Assay Name: CZ-3
Sample ID: 7
Note:

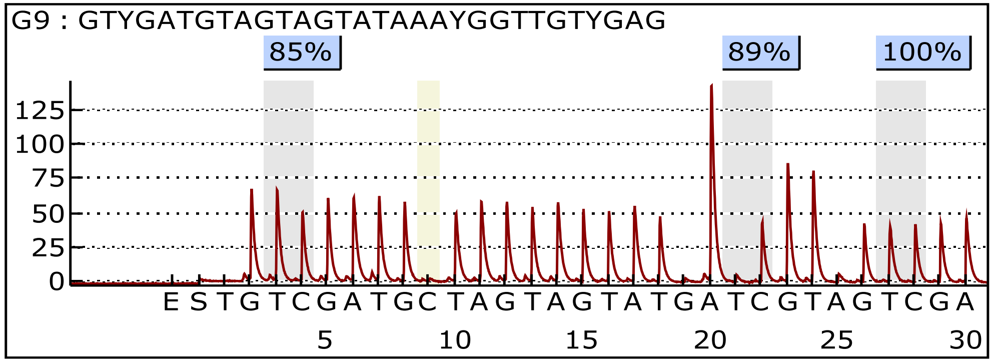


Assay Name: CZ-3
Sample ID: 11
Note:

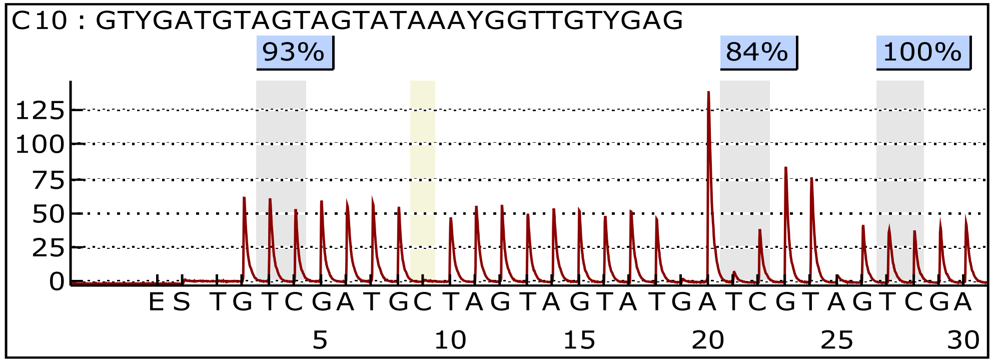

Assay Name: CZ-3
Sample ID: 12
Note:

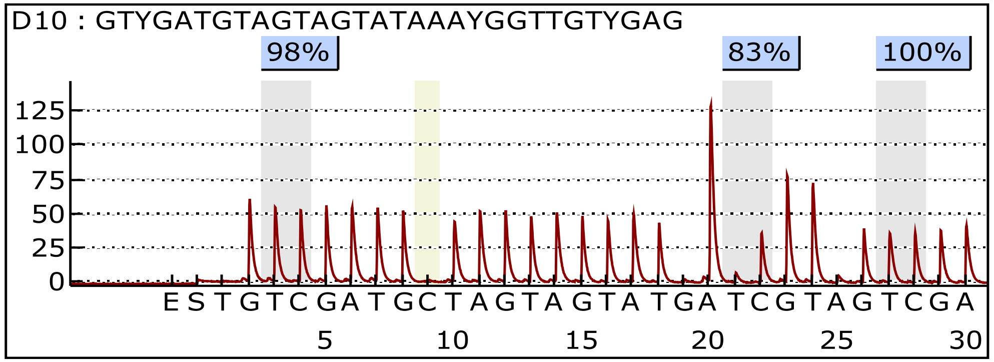


Assay Name: CZ-3
Sample ID: 13
Note:

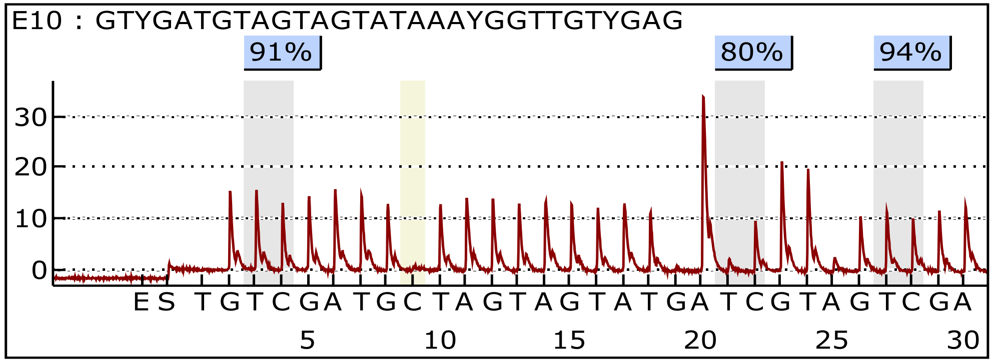


Assay Name: CZ-3
Sample ID: 17
Note:

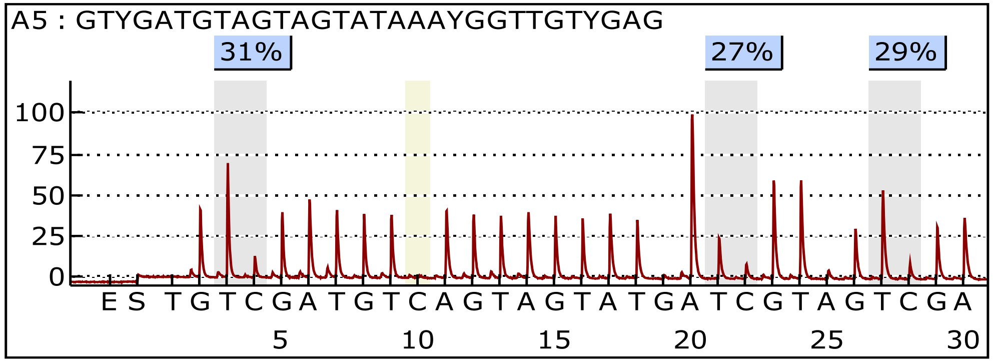


Assay Name: CZ-3
Sample ID: 18
Note:

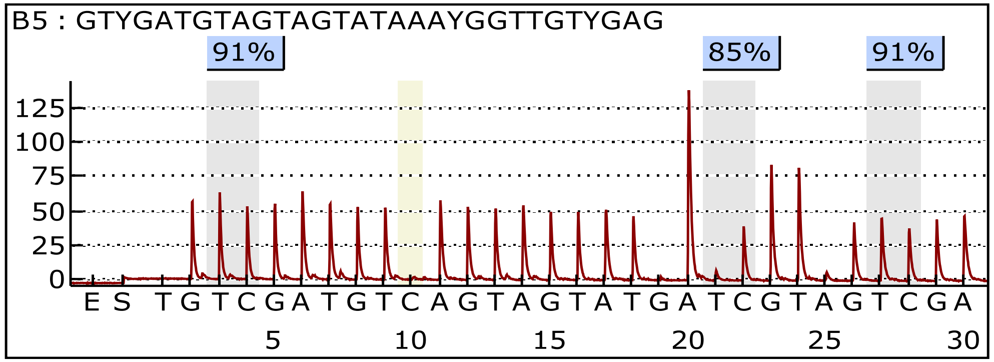


Assay Name: CZ-3
Sample ID: 19
Note:

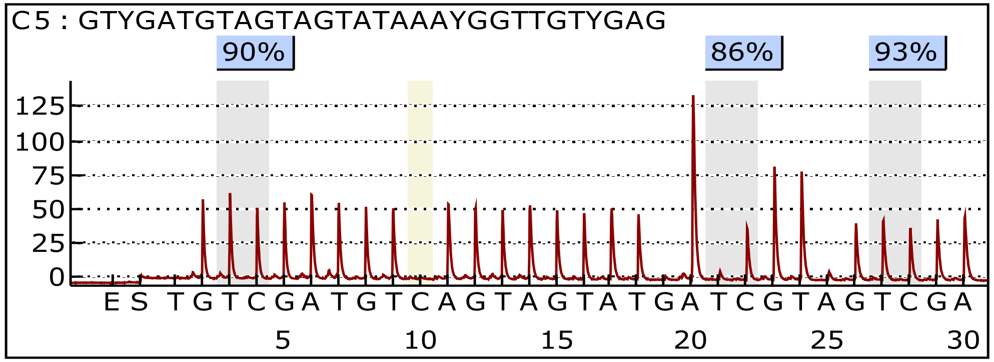


Assay Name: CZ-3
Sample ID: 20
Note:

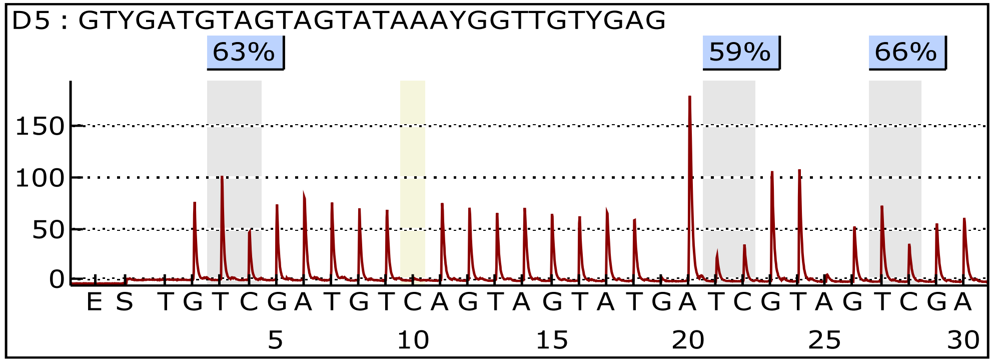

Assay Name: CZ-3
Sample ID: 21
Note:

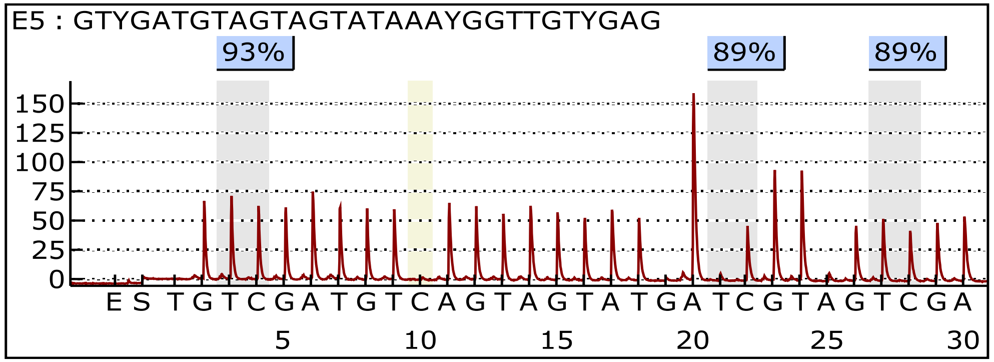


Assay Name: CZ-3
Sample ID: 22
Note:

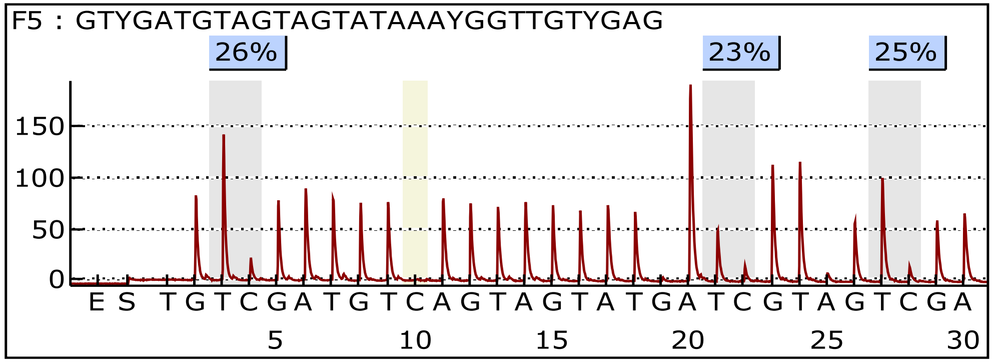


Assay Name: CZ-3
Sample ID: 23
Note:

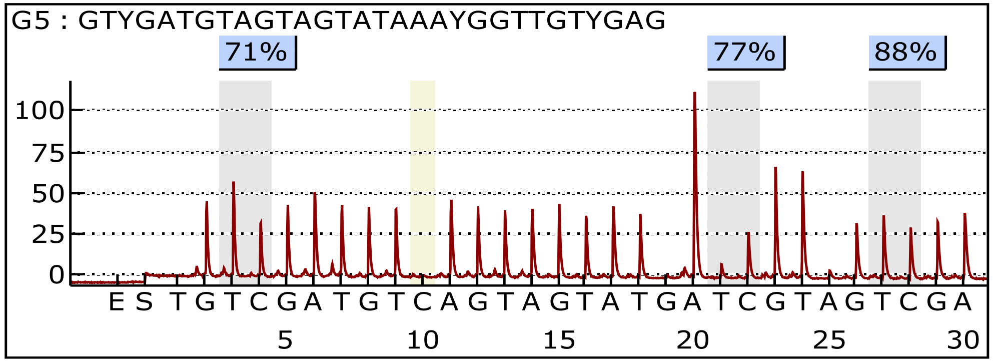


Assay Name: CZ-3
Sample ID: 24
Note:

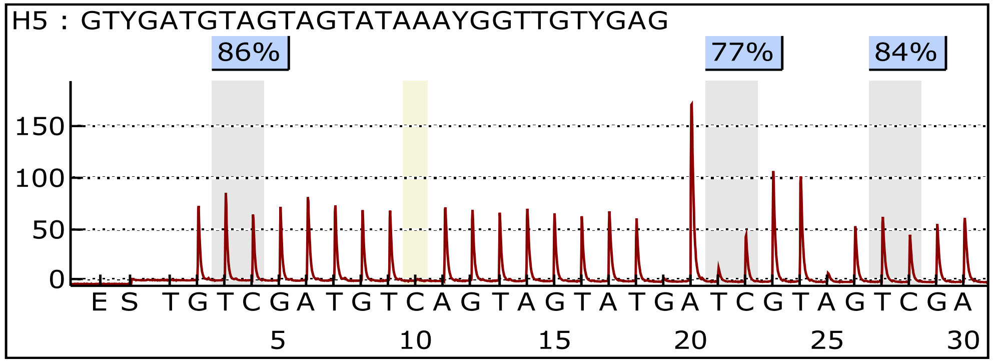


Assay Name: CZ-3
Sample ID: 56
Note:

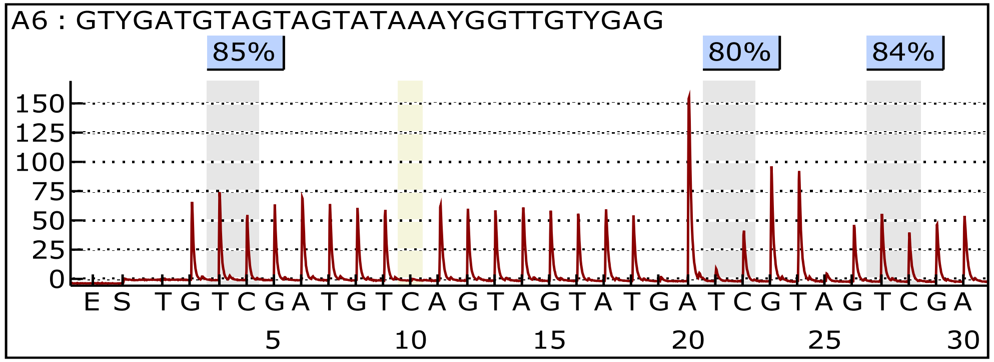


Assay Name: CZ-3
Sample ID: 57
Note:

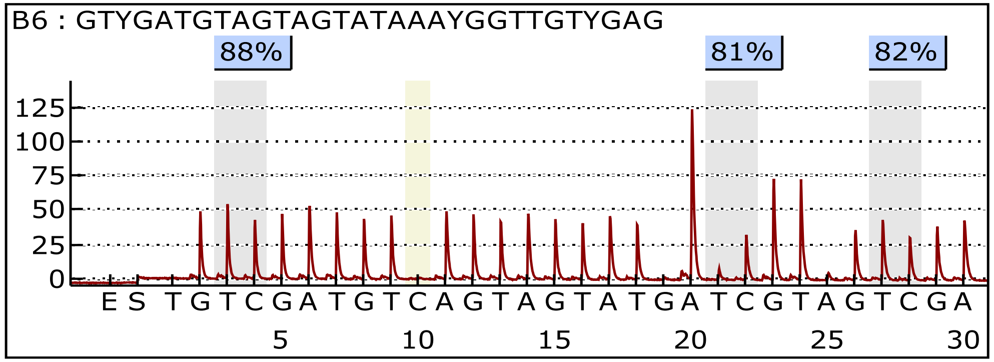

Assay Name: CZ-3
Sample ID: 58
Note:

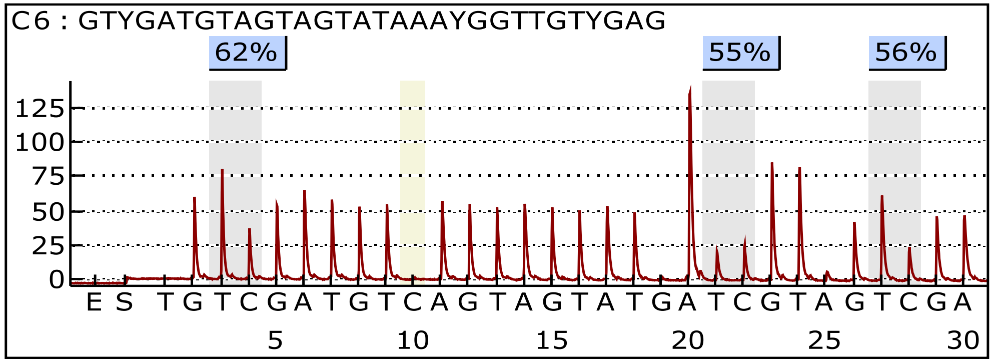


Assay Name: CZ-3
Sample ID: 59
Note:

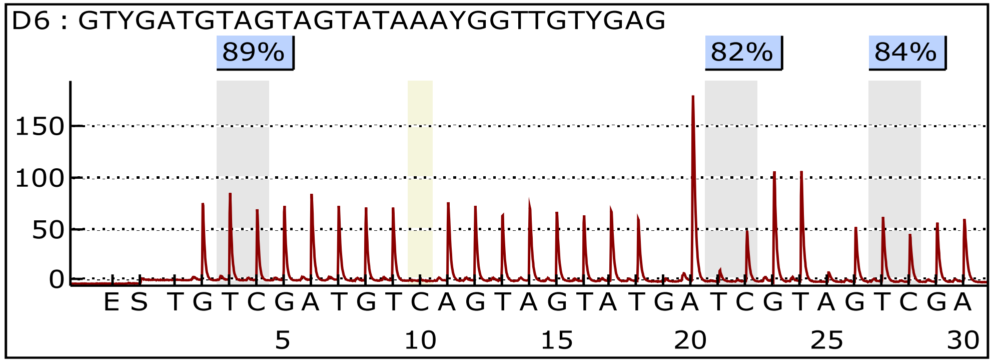


Assay Name: CZ-3
Sample ID: 60
Note:

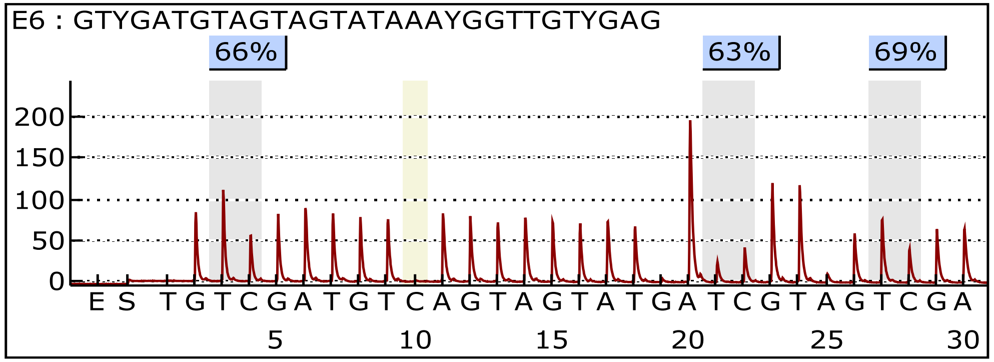


Assay Name: CZ-3
Sample ID: 61
Note:

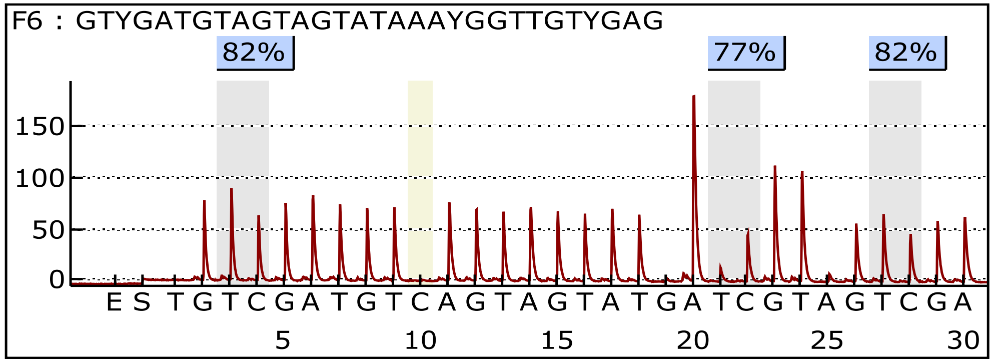


Assay Name: CZ-3
Sample ID: 62
Note:

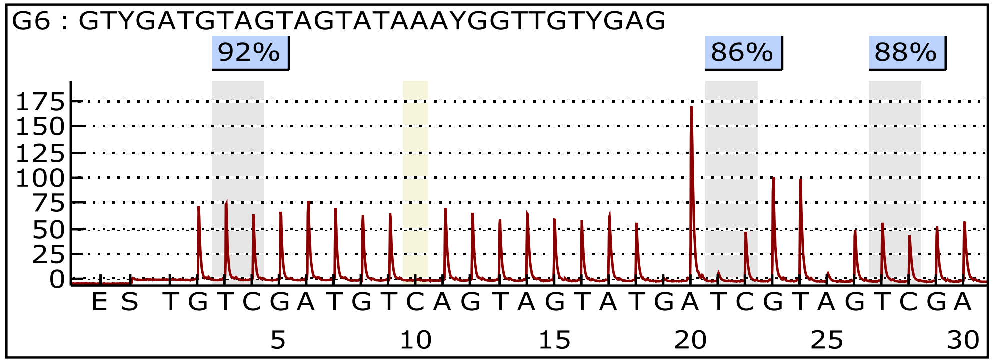


Assay Name: CZ-3
Sample ID: 63
Note:

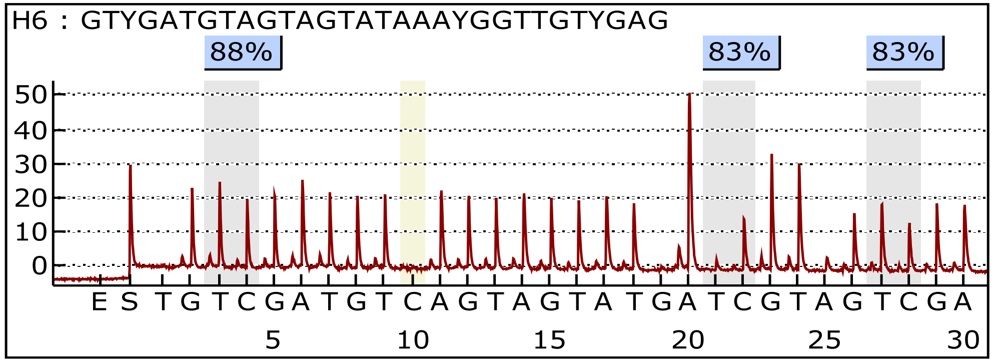

Assay Name: CZ-3
Sample ID: 64
Note:

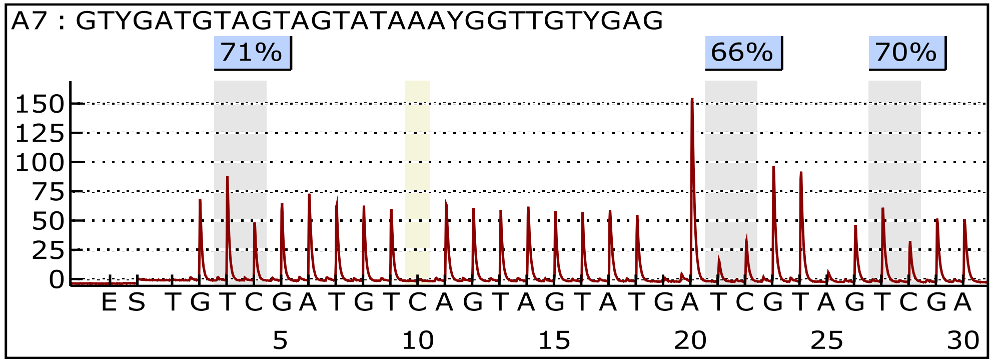


Assay Name: CZ-3
Sample ID: 65
Note:

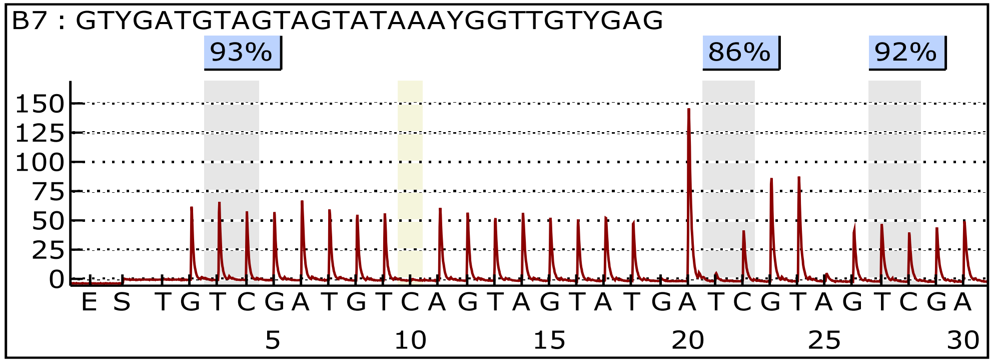


Assay Name: CZ-3
Sample ID: 66
Note:

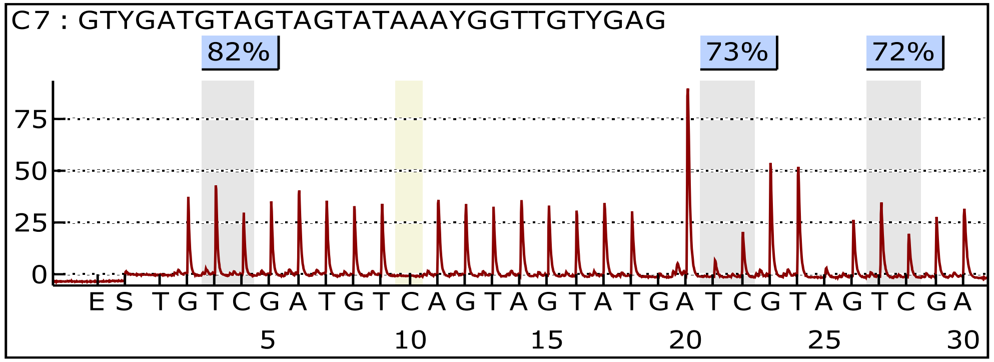


Assay Name: CZ-3
Sample ID: 67
Note:

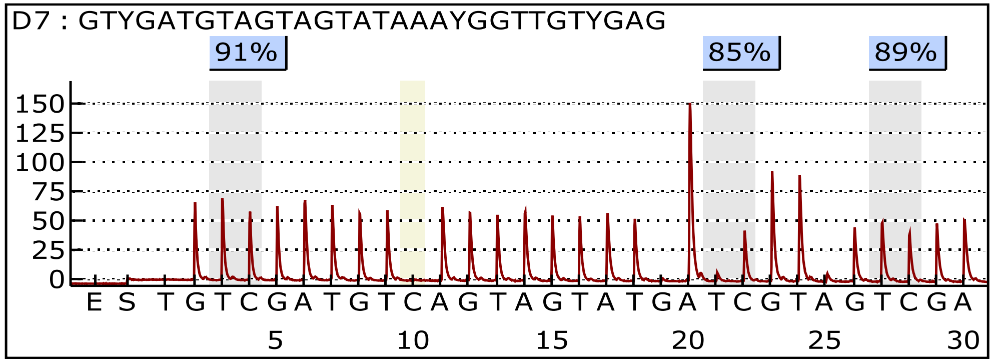


Assay Name: CZ-3
Sample ID: 68
Note:

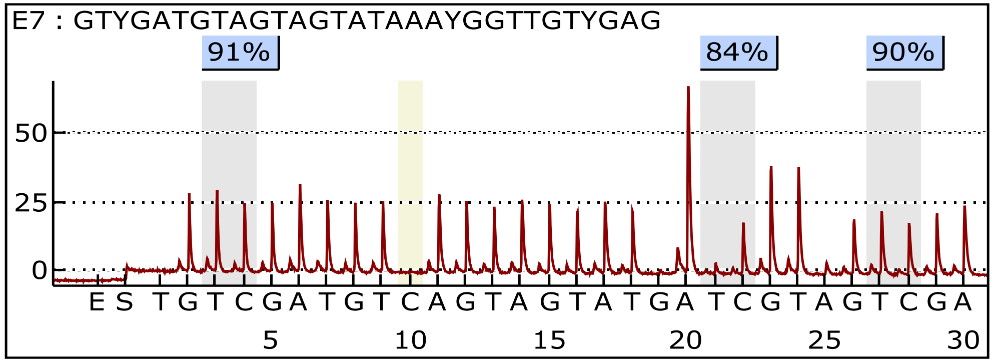


Assay Name: CZ-3
Sample ID: 69
Note:

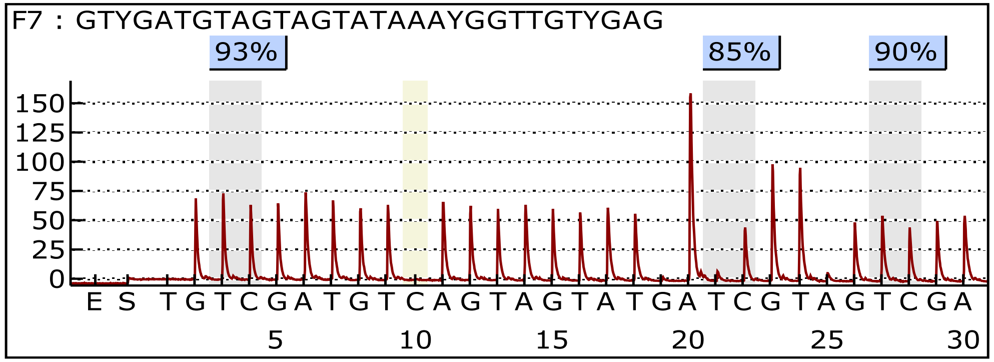

Assay Name: CZ-3
Sample ID: 70
Note:

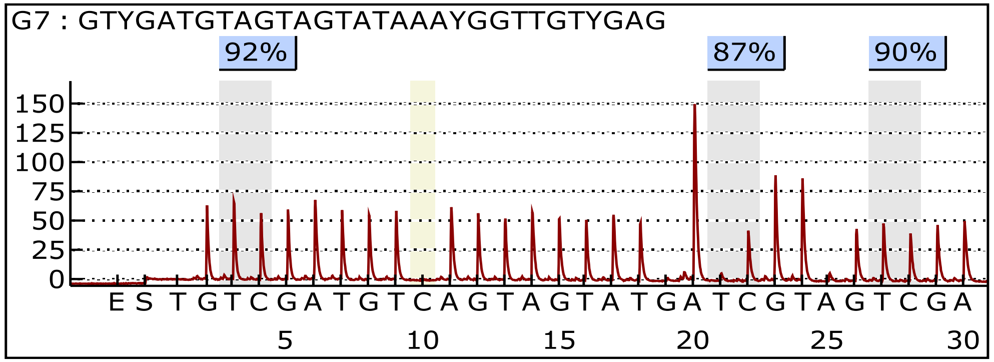


Assay Name: CZ-3
Sample ID: 71
Note:

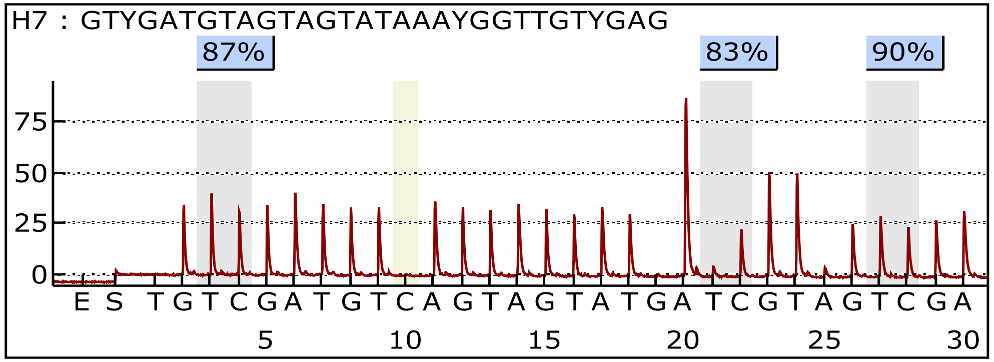


Assay Name: CZ-3
Sample ID: 72
Note:

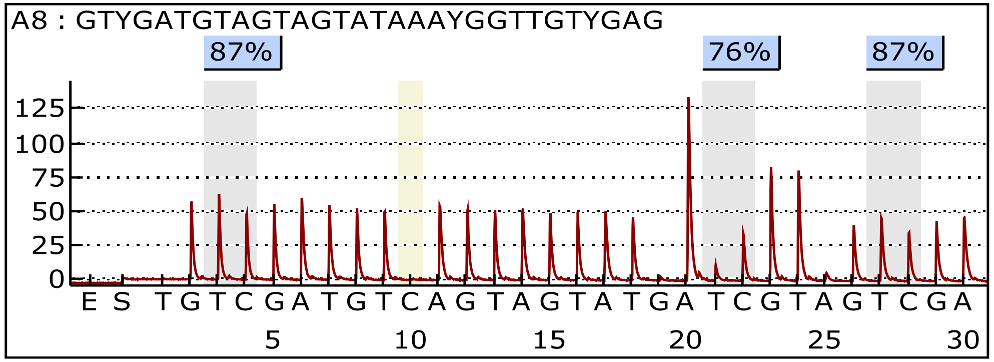


Assay Name: CZ-3
Sample ID: 73
Note:

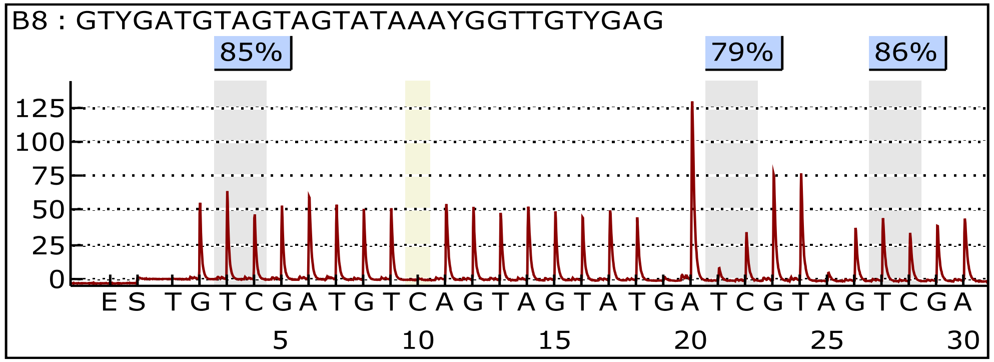


Assay Name: CZ-3
Sample ID: 74
Note:

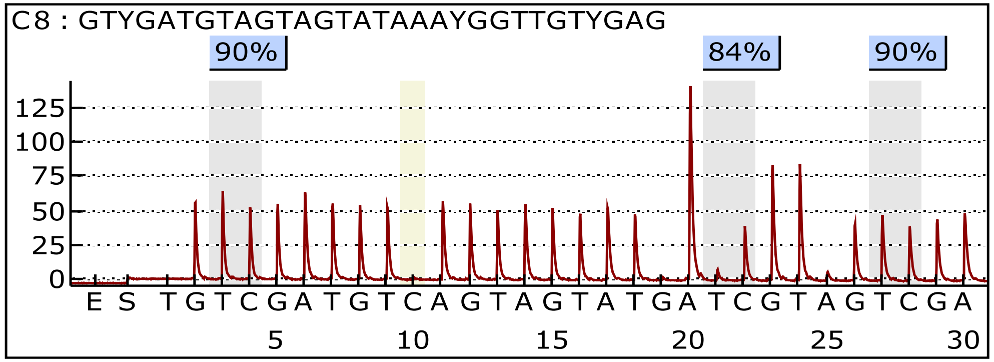


Assay Name: CZ-3
Sample ID: 75
Note:

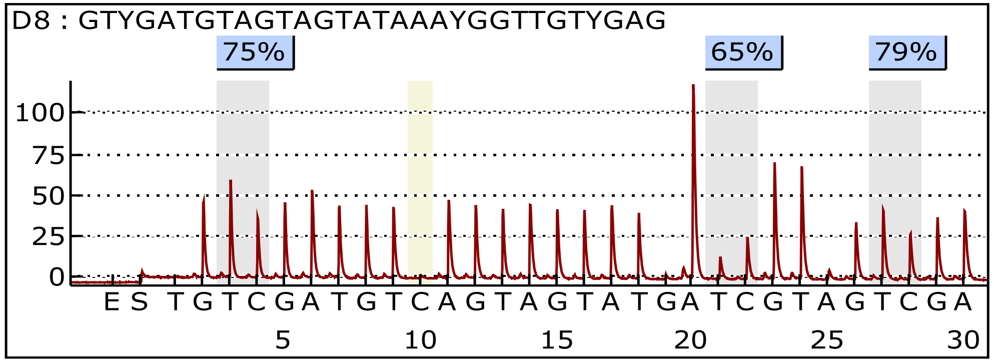

Assay Name: CZ-3
Sample ID: 76
Note:

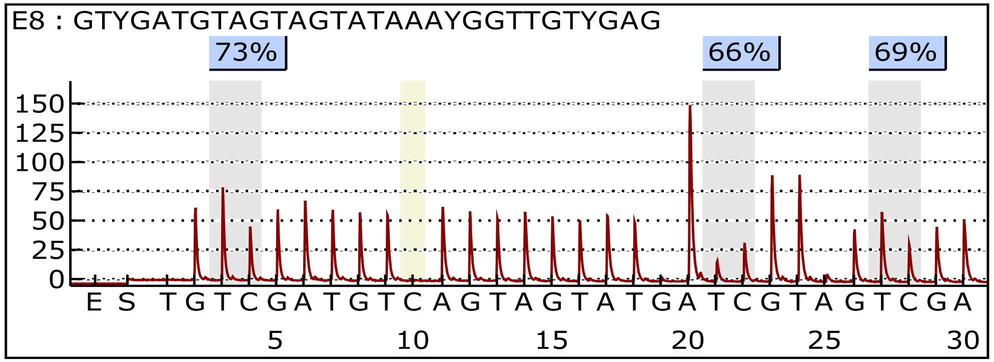


Assay Name: CZ-3
Sample ID: 77
Note:

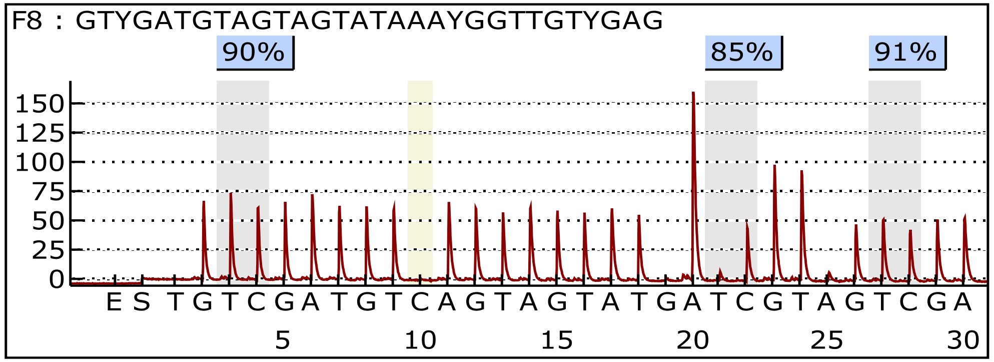


Assay Name: CZ-3
Sample ID: 1
Note:

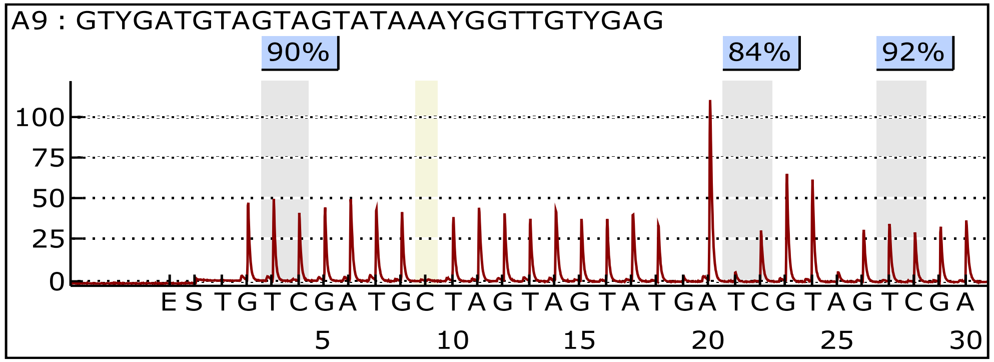


Assay Name: CZ-3
Sample ID: 2
Note:

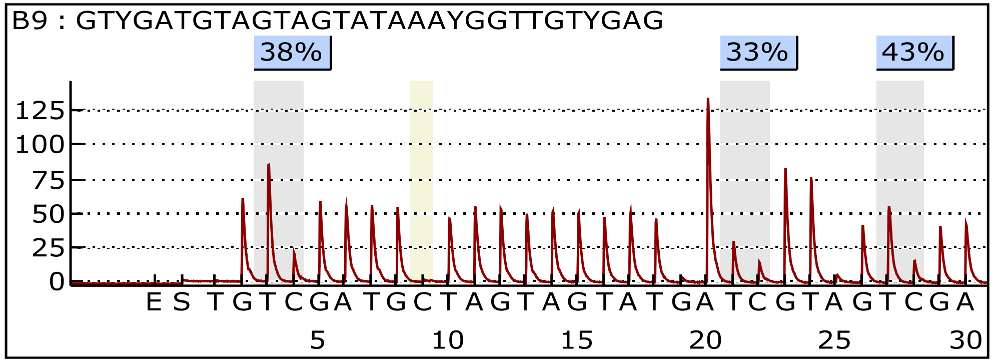


Assay Name: CZ-3
Sample ID: 3
Note:

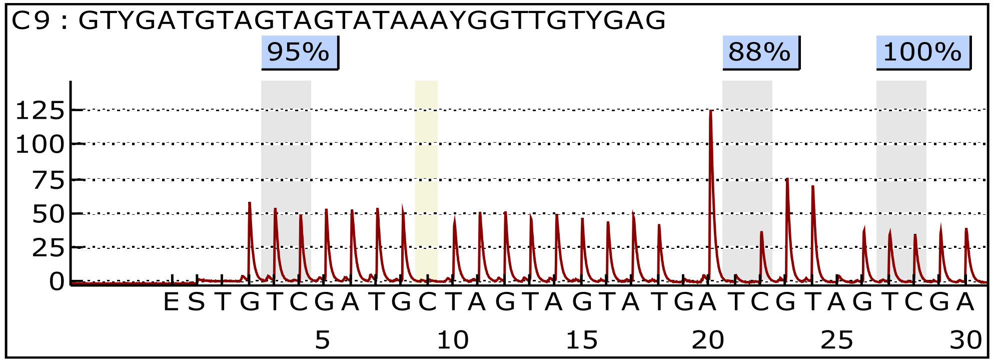

Assay Name: CZ-3
Sample ID: 4
Note:

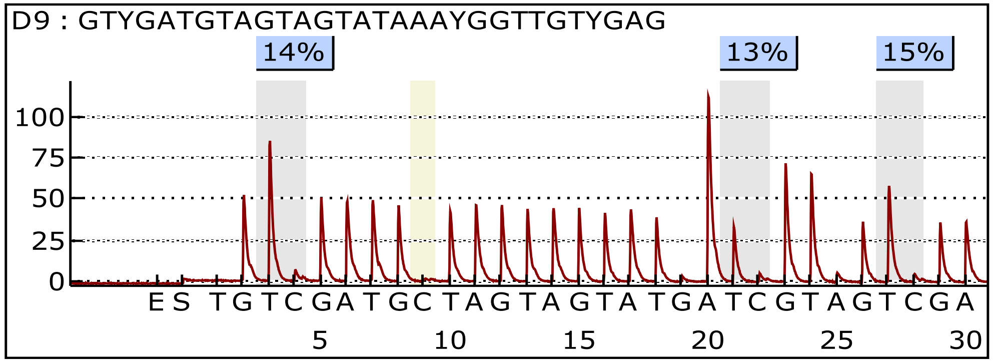


Assay Name: CZ-3
Sample ID: 15
Note:

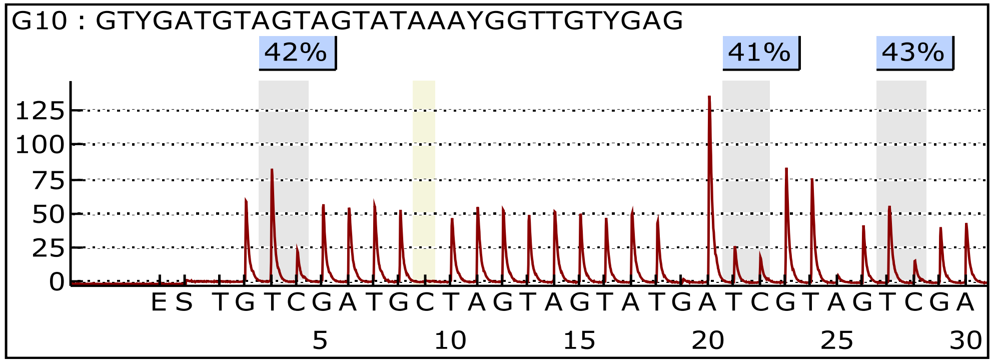


Assay Name: CZ-3
Sample ID: 10
Note:

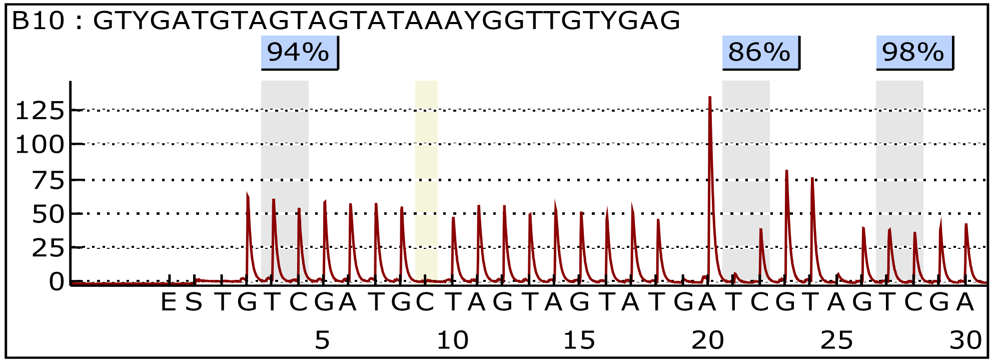


Assay Name: CZ-3
Sample ID: 16
Note:

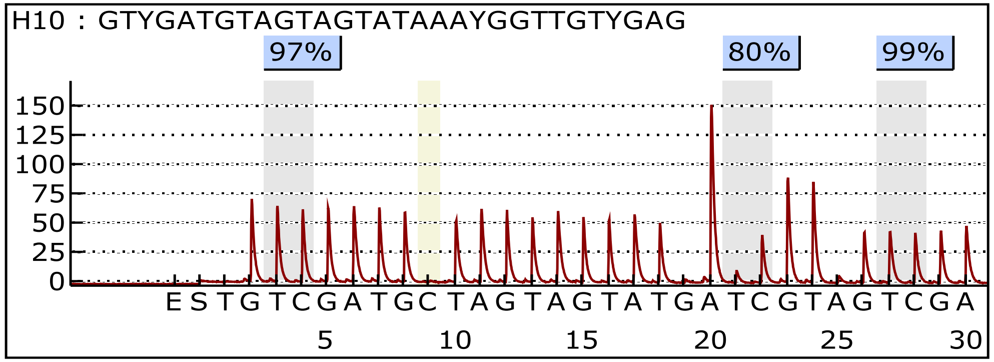


Assay Name: CZ-3
Sample ID: 14
Note:

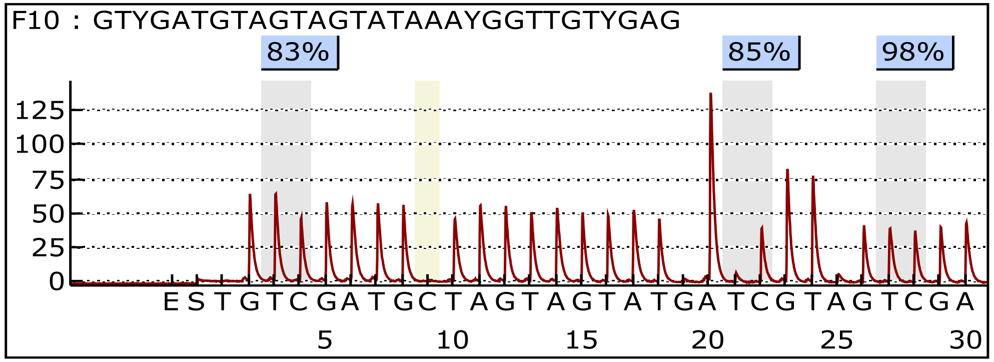


Assay Name: CZ-3
Sample ID: 25
Note:

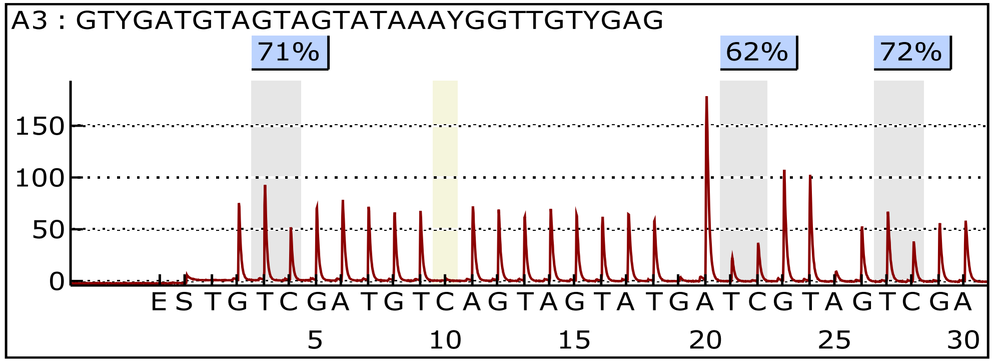

Assay Name: CZ-3
Sample ID: 26
Note:

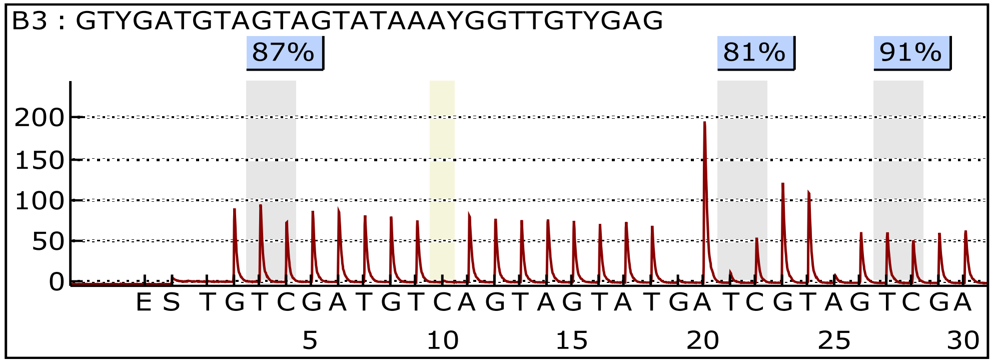


Assay Name: CZ-3
Sample ID: 27
Note:

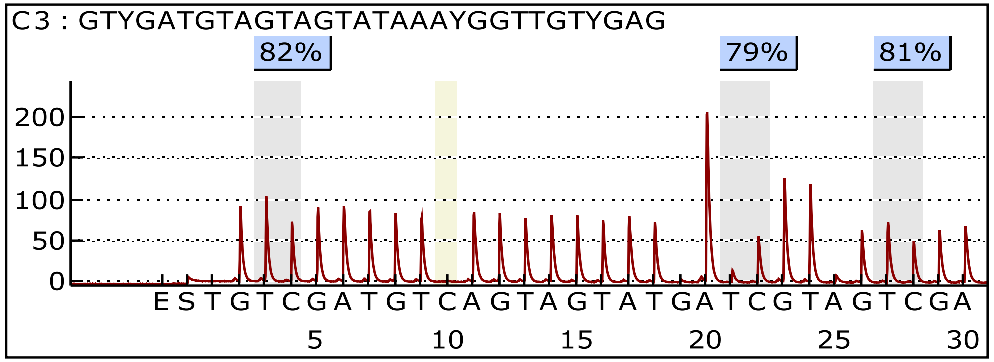


Assay Name: CZ-3
Sample ID: 28
Note:

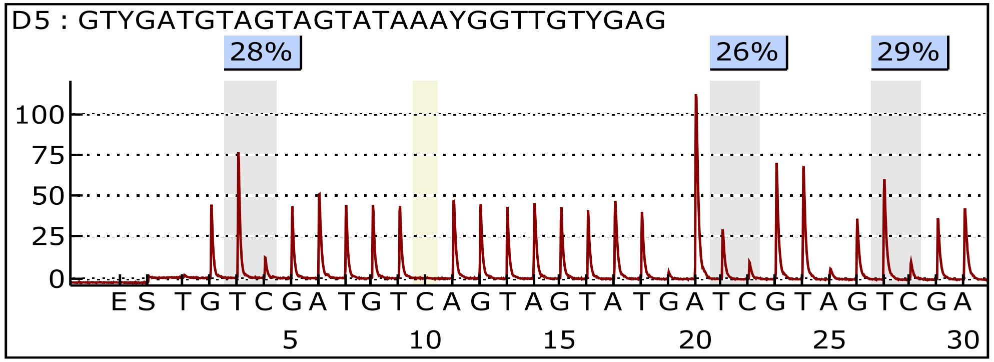


Assay Name: CZ-3
Sample ID: 29
Note:

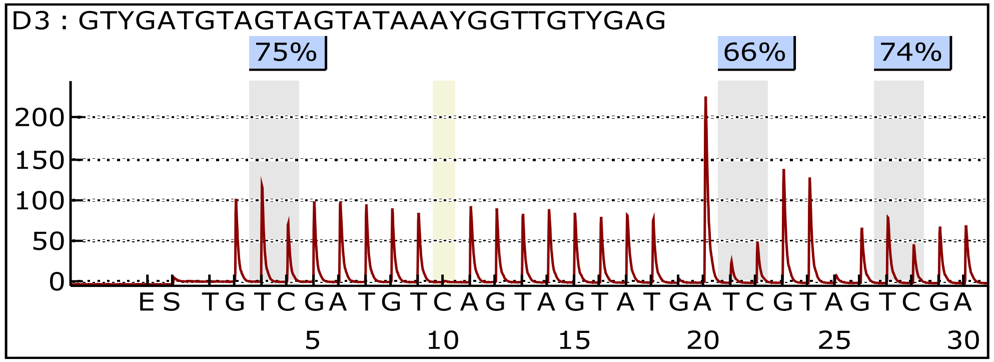


Assay Name: CZ-3
Sample ID: 30
Note:

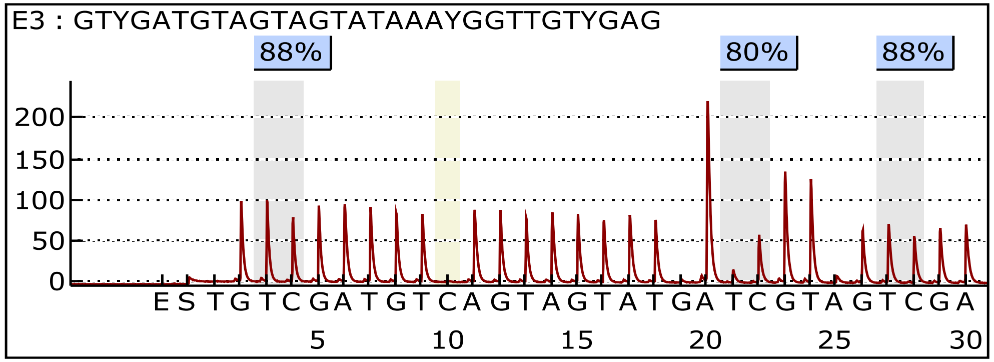


Assay Name: CZ-3
Sample ID: 31
Note:

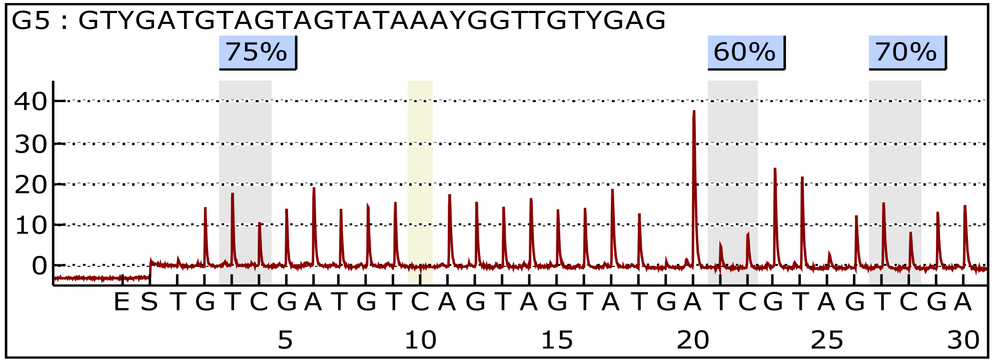

Assay Name: CZ-3
Sample ID: 32
Note:

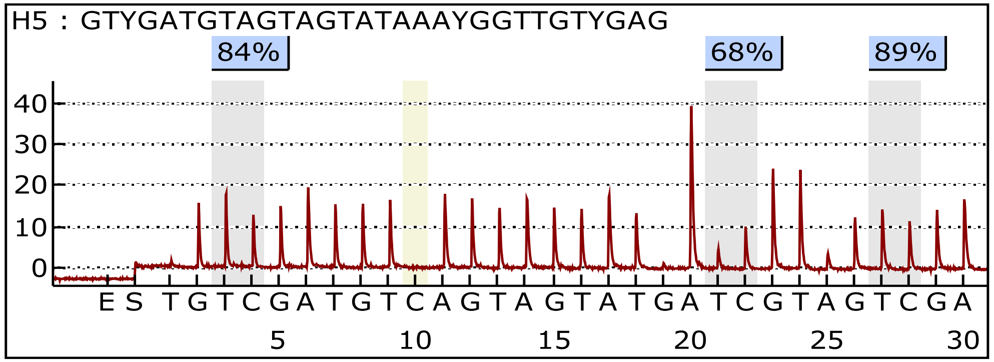


Assay Name: CZ-3
Sample ID: 36
Note:

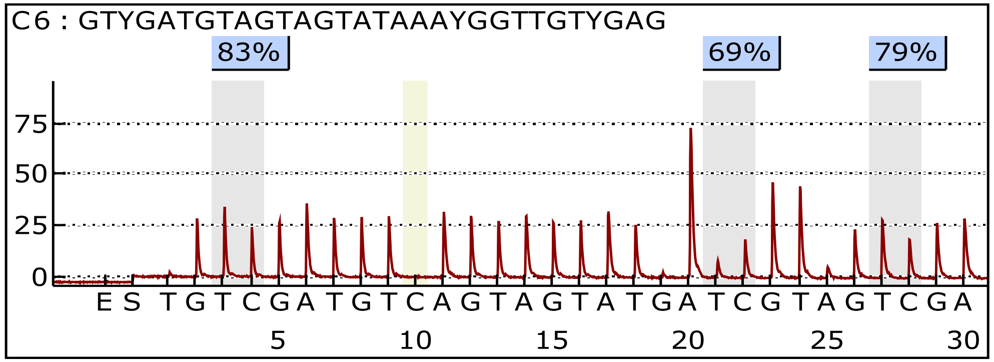


Assay Name: CZ-3
Sample ID: 34
Note:

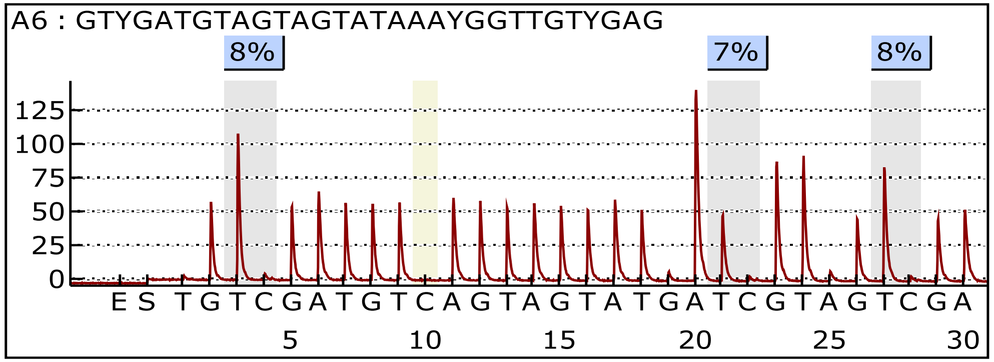


Assay Name: CZ-3
Sample ID: 37
Note:

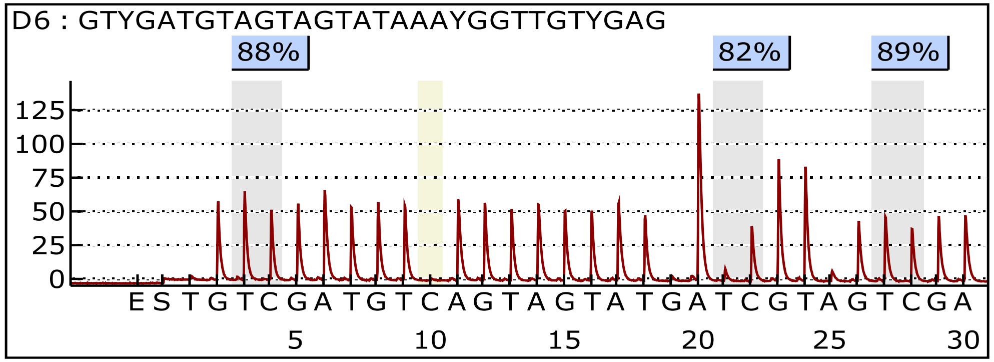


Assay Name: CZ-3
Sample ID: 35
Note:

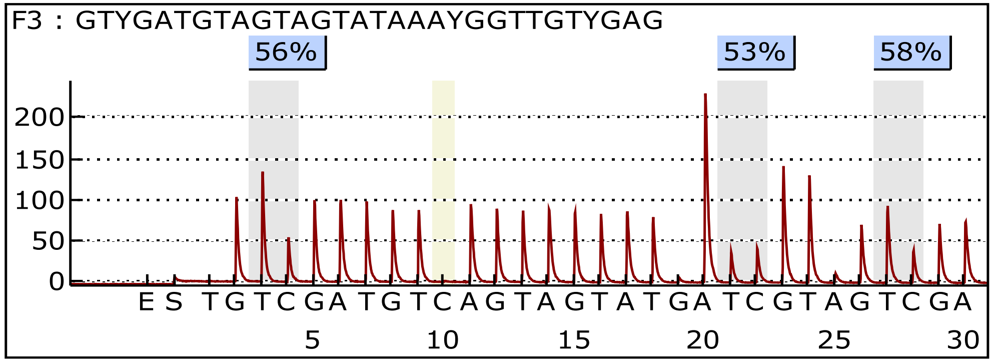


Assay Name: CZ-3
Sample ID: 38
Note:

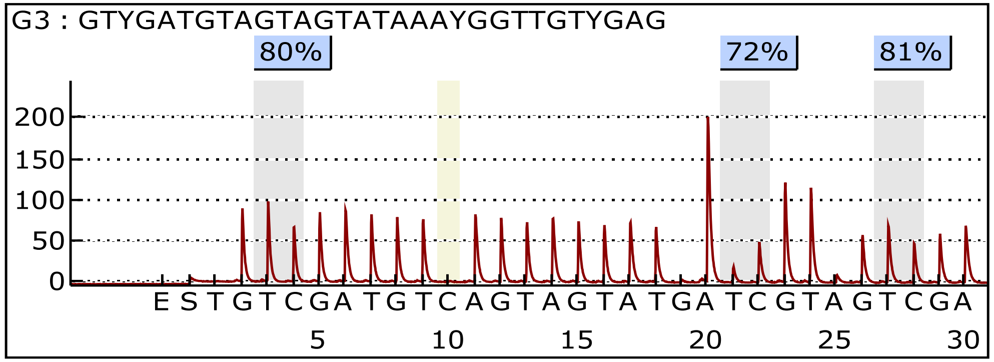

Assay Name: CZ-3
Sample ID: 39
Note:

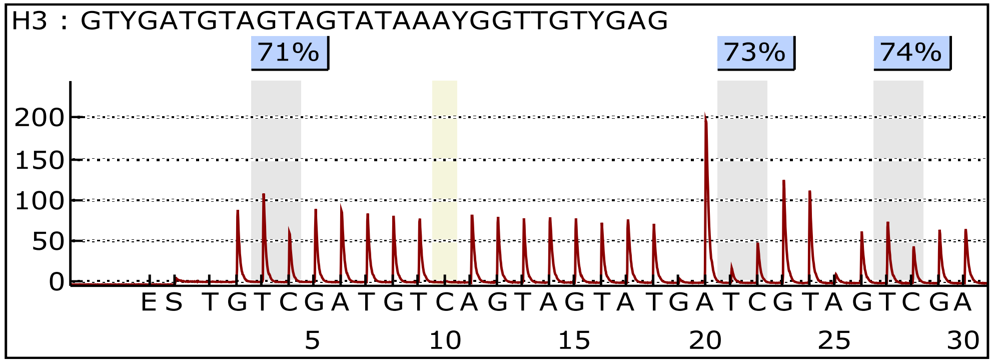


Assay Name: CZ-3
Sample ID: 40
Note:

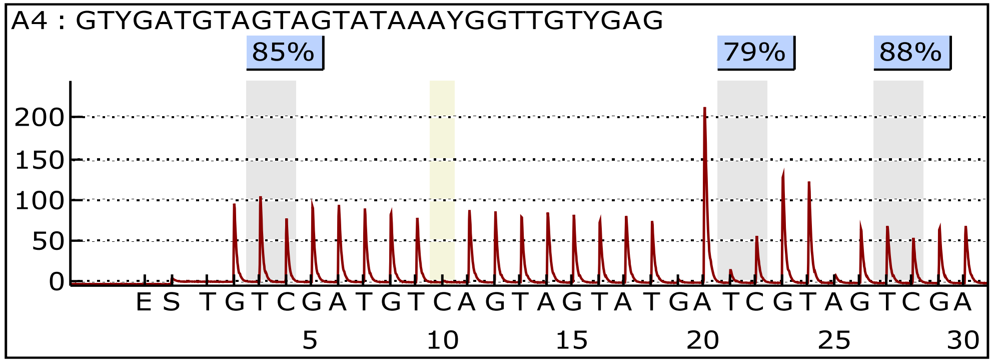


Assay Name: CZ-3
Sample ID: 41
Note:

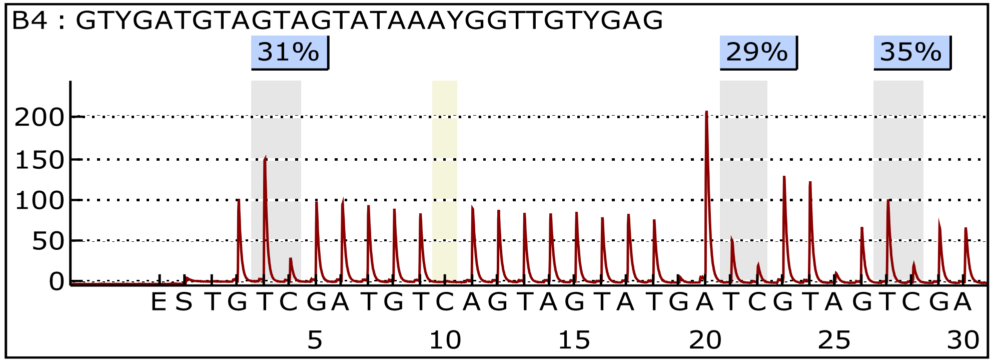


Assay Name: CZ-3
Sample ID: 42
Note:

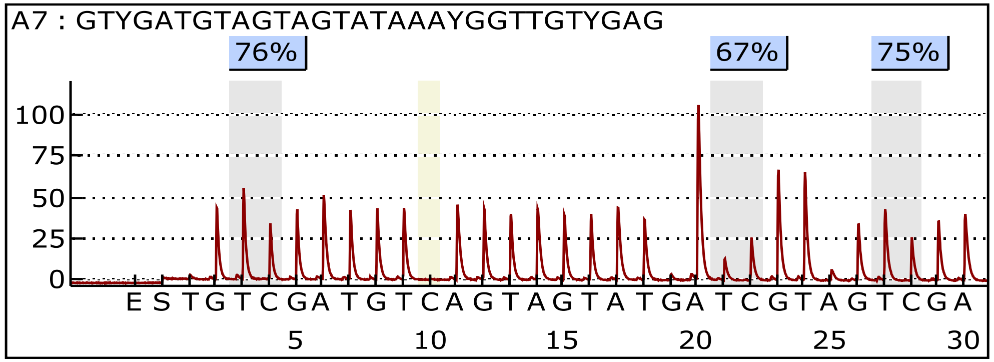


Assay Name: CZ-3
Sample ID: 43
Note:

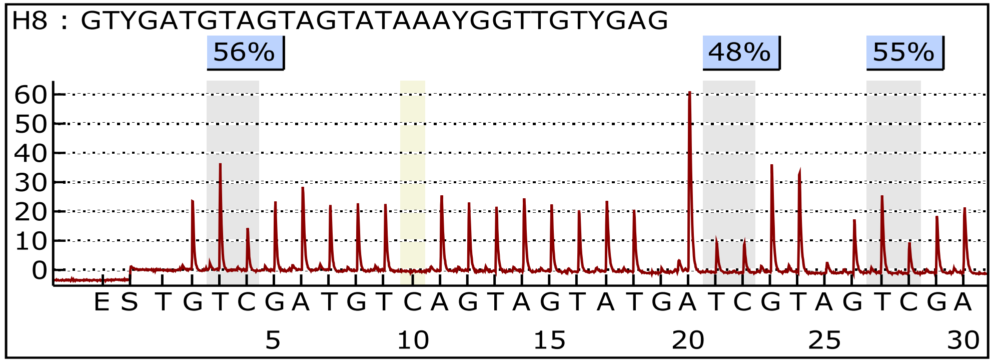


Assay Name: CZ-3
Sample ID: 44
Note:

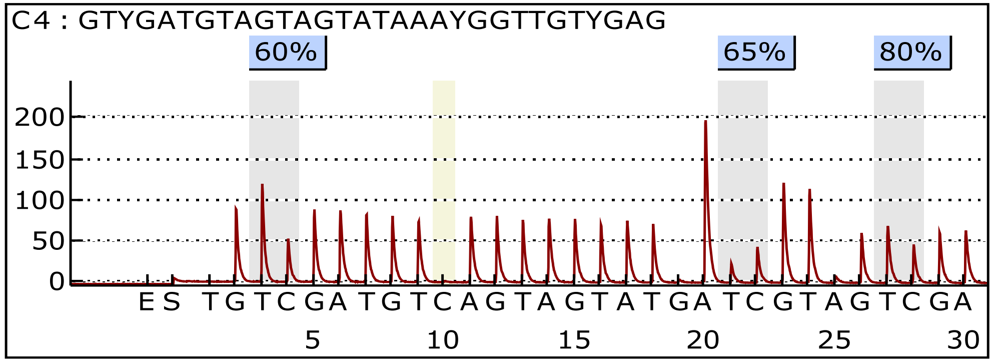

Assay Name: CZ-3
Sample ID: 45
Note:

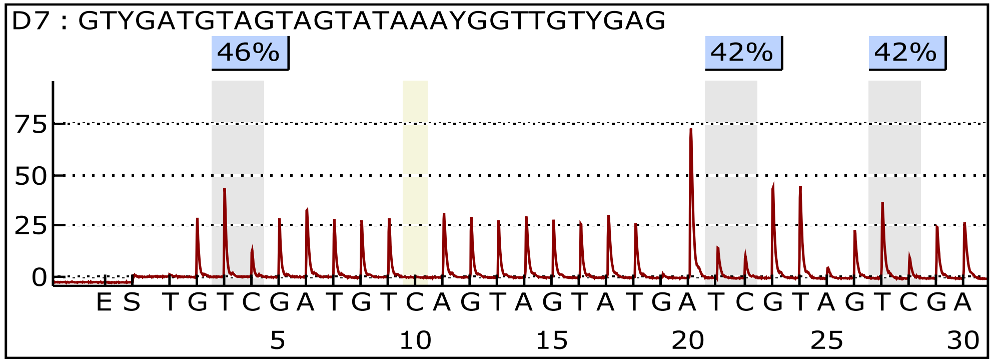


Assay Name: CZ-3
Sample ID: 46
Note:

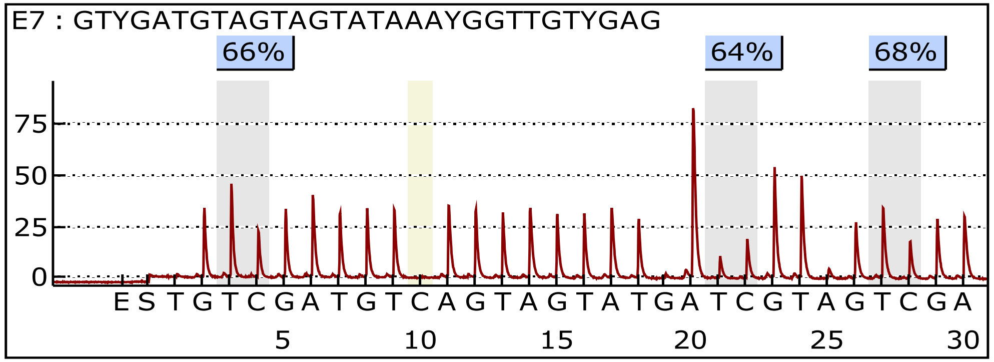


Assay Name: CZ-3
Sample ID: 47
Note:

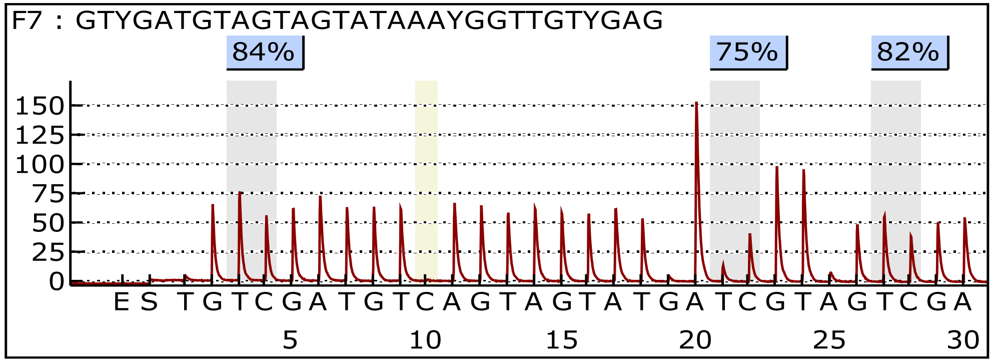


Assay Name: CZ-3
Sample ID: 48
Note:

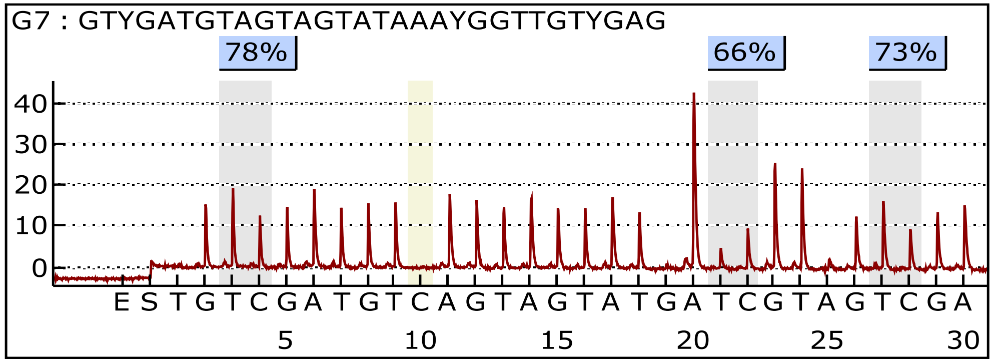


Assay Name: CZ-3
Sample ID: 49
Note:

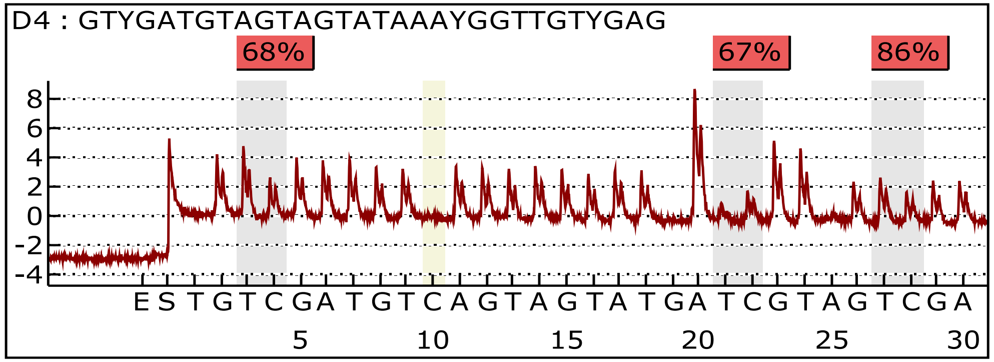


Assay Name: CZ-3
Sample ID: 50
Note:

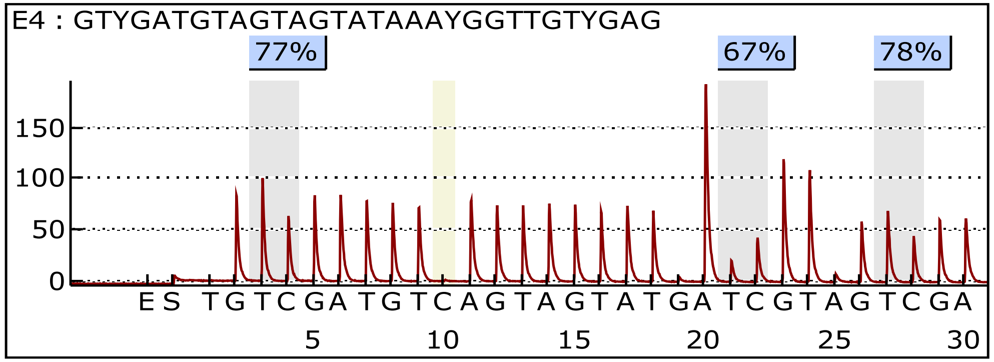

Assay Name: CZ-3
Sample ID: 51
Note:

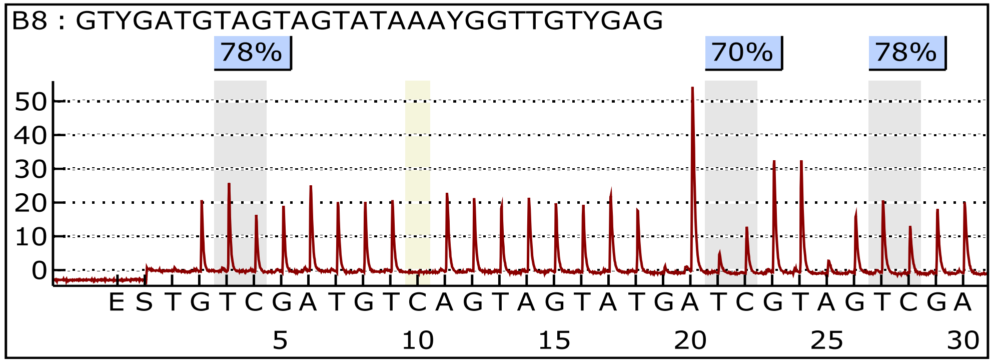


Assay Name: CZ-3
Sample ID: 52
Note:

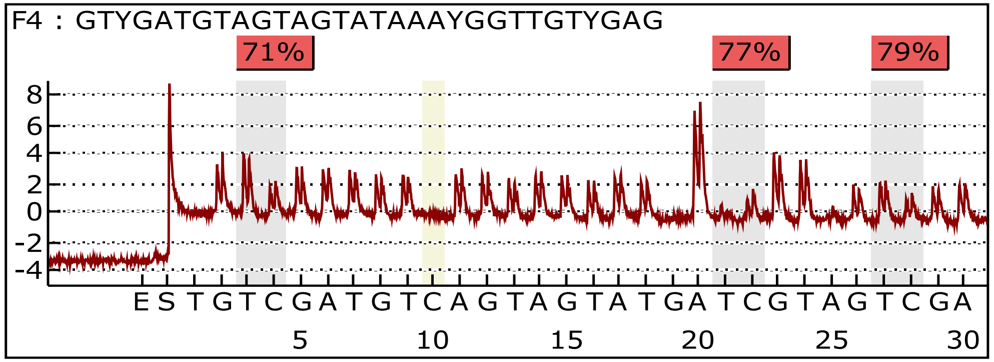


Assay Name: CZ-3
Sample ID: 53
Note:

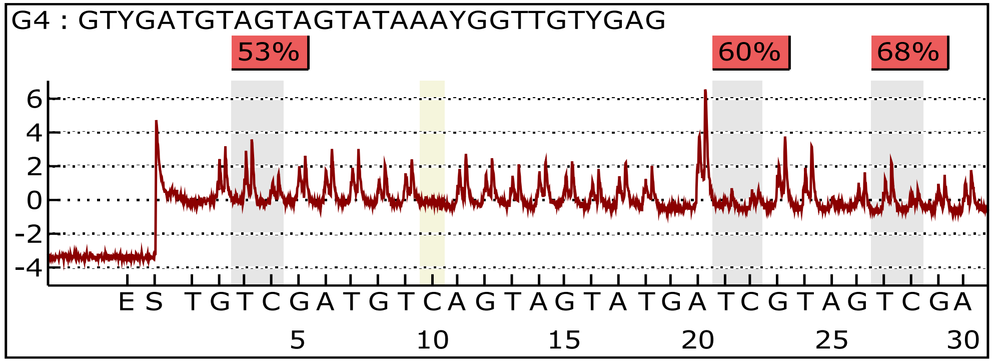


Assay Name: CZ-3
Sample ID: 54
Note:

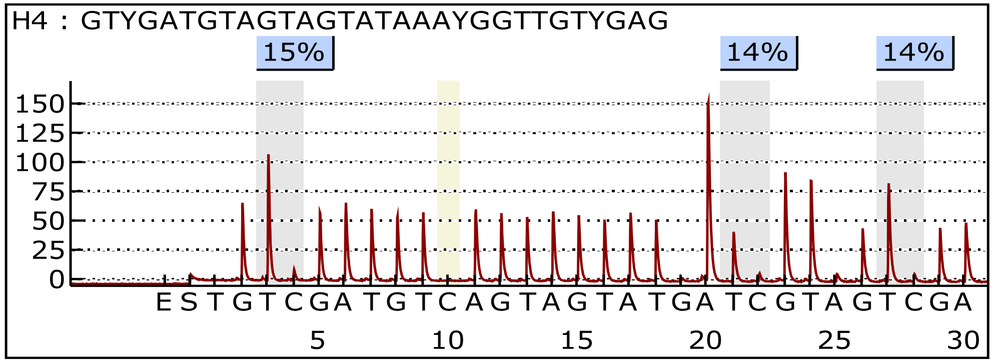


Assay Name: CZ-3
Sample ID: 55
Note:

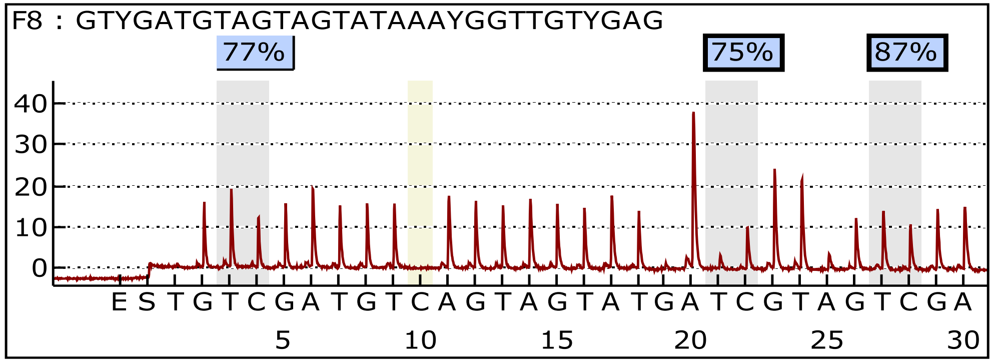

Supplement: Supplementary file 7 [file oncotarget-06-40611-s007.pdf]
